# Thermally Induced Solid-Phase Quasi-Intramolecular Redox Reactions of [Hexakis(urea-*O*)iron(III)] Permanganate: An Easy Reaction Route to Prepare Potential (Fe,Mn)O_*x*_ Catalysts for CO_2_ Hydrogenation

**DOI:** 10.1021/acs.inorgchem.2c02265

**Published:** 2022-08-31

**Authors:** Kende Attila Béres, Zoltán Homonnay, Libor Kvitek, Zsolt Dürvanger, Martina Kubikova, Veronika Harmat, Fanni Szilágyi, Zsuzsanna Czégény, Péter Németh, Laura Bereczki, Vladimir M. Petruševski, Mátyás Pápai, Attila Farkas, László Kótai

**Affiliations:** † Institute of Materials and Environmental Chemistry, Research Centre for Natural Sciences, Magyar Tudósok krt. 2, H-1117 Budapest, Hungary; ‡ György Hevesy PhD School of Chemistry, Institute of Chemistry, ELTE Eötvös Loránd University, Pázmány Péter s. 1/A, H-1117 Budapest, Hungary; § Faculty of Science, Department of Physical Chemistry, Palacky University Olomouc, 17. Listopadu 12, Olomouc 77146, Czech Republic; ∥ Structural Chemistry and Biology Laboratory, Institute of Chemistry, ELTE Eötvös Loránd University, Pázmány Péter s. 1/A, H-1117 Budapest, Hungary; ⊥ ELKH-ELTE Protein eModelling Research Group, Pázmány Péter s. 1/A, H-1117 Budapest, Hungary; # Bay Zoltan Ltd. for Applied Research, Production Division (BAY-PROD), 1 Kondorfa, H-1116 Budapest, Hungary; ∇ Institute for Geological and Geochemical Research, Research Centre for Astronomy and Earth Sciences, ELKH, Budaörsi street 45, H-1112 Budapest, Hungary; ○ Faculty of Natural Sciences and Mathematics, Ss. Cyril and Methodius University, Skopje MK-1000, North Macedonia; ◆ Wigner Research Centre for Physics, H-1525 Budapest, P.O. Box 49, Hungary; ¶ Department of Organic Chemistry, Budapest University of Technology and Economics, Műegyetem rakpart 3, H-1111 Budapest, Hungary; & Deuton-X Ltd., Selmeci u. 89, H-2030, Érd, Hungary

## Abstract

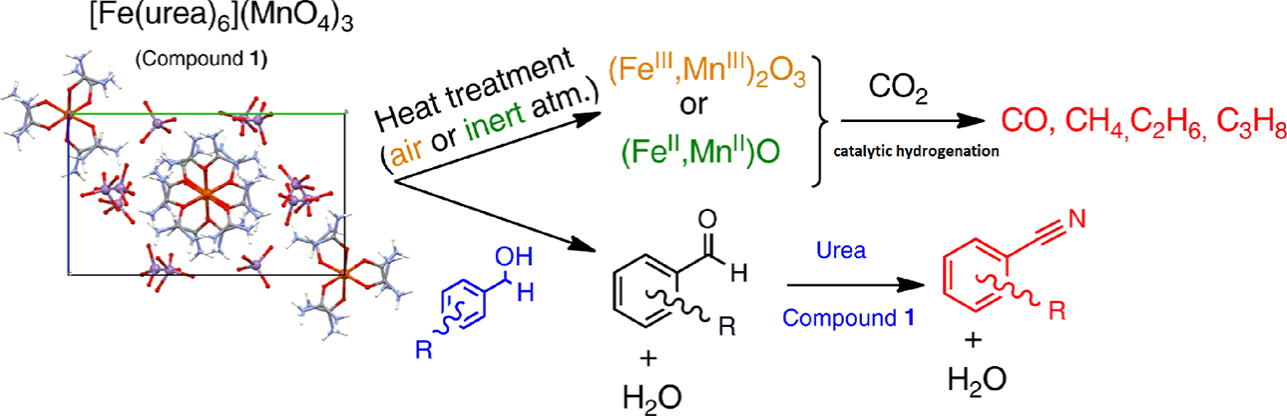

Research on new reaction routes and precursors to prepare catalysts for CO_2_ hydrogenation has enormous importance. Here, we report on the preparation of the permanganate salt of the urea-coordinated iron(III), [hexakis(urea-*O*)iron(III)]permanganate ([Fe(urea-O)_6_](MnO_4_)_3_) via an affordable synthesis route and preliminarily demonstrate the catalytic activity of its (Fe,Mn)O_*x*_ thermal decomposition products in CO_2_ hydrogenation. [Fe(urea-O)_6_](MnO_4_)_3_ contains O-coordinated urea ligands in octahedral propeller-like arrangement around the Fe^3+^ cation. There are extended hydrogen bond interactions between the permanganate ions and the hydrogen atoms of the urea ligands. These hydrogen bonds serve as reaction centers and have unique roles in the solid-phase quasi-intramolecular redox reaction of the urea ligand and the permanganate anion below the temperature of ligand loss of the complex cation. The decomposition mechanism of the urea ligand (ammonia elimination with the formation of isocyanuric acid and biuret) has been clarified. In an inert atmosphere, the final thermal decomposition product was manganese-containing wuestite, (Fe,Mn)O, at 800 °C, whereas in ambient air, two types of bixbyite (Fe,Mn)_2_O_3_ as well as jacobsite (Fe,Mn)^T-4^(Fe,Mn)^OC-6^_2_O_4_), with overall Fe to Mn stoichiometry of 1:3, were formed. These final products were obtained regardless of the different atmospheres applied during thermal treatments up to 350 °C. Disordered bixbyite formed first with inhomogeneous Fe and Mn distribution and double-size supercell and then transformed gradually into common bixbyite with regular structure (and with 1:3 Fe to Mn ratio) upon increasing the temperature and heating time. The (Fe,Mn)O_*x*_ intermediates formed under various conditions showed catalytic effect in the CO_2_ hydrogenation reaction with <57.6% CO_2_ conversions and <39.3% hydrocarbon yields. As a mild solid-phase oxidant, hexakis(urea-*O*)iron(III) permanganate, was found to be selective in the transformation of (un)substituted benzylic alcohols into benzaldehydes and benzonitriles.

## Abstract 2

[Fe(urea-*O*)_6_](MnO_4_)_3_ is a selective solid-phase oxidant of benzylic alcohols into benzaldehydes and precursor in the preparation of (Fe,Mn)O_*x*_ catalysts for CO_2_ hydrogenation into hydrocarbons. The urea ligands are in octahedral propeller-like arrangement around the Fe^3+^ cation, and there are hydrogen bonds between the permanganate anions and the urea ligands. A solid-phase quasi-intramolecular redox reaction of the urea and the permanganate resulted in (Fe,Mn)O, (Fe,Mn)_2_O_3_, and (Fe,Mn)^T-4^(Fe,Mn)^OC-6^_2_O_4_ with an overall Fe to Mn stoichiometry of 1:3.

## Introduction

The heat-induced quasi-intramolecular redox reactions of [ML_*n*_](XO_4_)_*m*_ complexes containing reducing ligands and oxidizing anions (where M = Cu, Zn, Cd, Co; L = NH_3_ or pyridine, *n* = 2–6, and *m* = 0.5–3, and X = Mn, Cl, S, or Mo) in a solid phase ensure a convenient way to prepare simple or mixed-transition metal oxides with nanometer-sized particles.^1−12^ These oxides, including nanosized iron oxide composites,^13−17^ are active catalysts in various industrially important processes as their structures contain defects and their metallic components have variable valence states.^18−20^

Iron–manganese oxides are active catalysts in important technological processes such as Fischer–Tropsch synthesis^21−23^ or the transformation of CO_2_ by hydrogenation into valuable chemicals and fuels.^24−26^ Since iron(III) favors an octahedral oxygen-coordination environment and does not form very stable NH_3_ or pyridine complexes, we selected urea as an O-coordinating reducing ligand to prepare a compound containing a reducing ligand and oxidizing anion, namely [hexakis(urea-*O*)iron(III)] permanganate, [Fe(H_2_NCONH_2_)_6_](MnO_4_)_3_ (compound **1**). Since iron(III)-coordinated urea can easily be oxidized by nitrate ions in [Fe(urea-O)_6_](NO_3_)_3_ with the formation of iron(III) oxide nanoparticles,^27,28^ we expected and found that the permanganate ion could oxidize the urea ligand in compound **1** with the formation of (Fe,Mn)O_*x*_ mixed oxides with Fe:Mn = 1:3 overall stoichiometry. However, the process and mechanisms of the reactions are unknown. In addition, the formation of (Fe,Mn)O_*x*_ mixed oxides could be relevant for preparing promising catalysts for technological applications. In this work, we studied the structure, oxidation abilities, and decomposition reaction of compound **1** and the catalytic activity of the mixed oxides formed by the decomposition of compound **1** in the hydrogenation of CO_2_ into hydrocarbons. Here, we demonstrate an easy way to prepare (Fe,Mn)O_*x*_ catalysts and draw attention to opening a reaction route to prepare catalysts, which increase CO_2_ conversion into hydrocarbons, especially to reach high hydrocarbon selectivity.

## Results and Discussion

### Preparation and Properties of Compound **1**

Divalent iron is oxidized easily with permanganates in aqueous solutions; therefore, Kótai et al. were only able to prepare a permanganate salt of trivalent iron in the reaction of FeOOH and in situ-prepared permanganic acid from Mn_2_O_7_ in a two-phase H_2_O/CCl_4_ system.^29^ The only known permanganate compound of complexed iron is [hexakis(urea-*O*)iron(III)] permanganate (compound **1**) prepared by Barbieri^30^ in 1913 as a blackish crystalline water-soluble material in the reaction of [hexakis(urea-*O*)iron(III)] nitrate and an excess of saturated sodium permanganate. No yield or any properties of the product were given. In a similar reaction, at room temperature, we used 3 equiv of 40% aq NaMnO_4_ and a saturated solution of iron(III) nitrate and 6 equiv of urea, and a purplish black precipitate of compound **1** was separated immediately:

Fe(NO_3_)_3_·9H_2_O + 6H_2_NCONH_2_ + 3NaMnO_4_ = [Fe(H_2_NCONH_2_)_6_](MnO_4_)_3_ + 3NaNO_3_ + 9H_2_O

The yield was 62%. The powder XRD data are given in ESI Tables S1 and S2 and ESI Figure S1. We refined the PXRD data and got a possible trigonal cell with *a =* 18.1241 Å, *b =* 18.1241, and Å *c =* 13.5493 Å and a possible monoclinic cell with *a* = 13.8479, *b* = 18.1275, and *c* = 11.4037; β = 64.122° cell constants. The single-crystal measurements on compound **1** and refinement in the trigonal system resulted in a large cell with doubled *a* and *b* cell dimensions (*a =* 36.2116 Å, *b =* 36.2116 Å, and *c =* 13.6365 Å); in other words, further refinement (due to pseudosymmetries and the disorder of the permanganate ions) resulted in a monoclinic cell (*P*2_1_/*c*, *a* = 13.7008(7) Å, *b* = 18.0084(5) Å, *c* = 11.4125(4)Å, β = 112.988(5)°, *V* = 2592.2(2)Å^3^, *Z* = 4, *d*_calcd_. = 1.981 g cm^–3^ (ESI Table S3)), where the cell volume and the *Z* value are in accordance with the pycnometric density value found to be 1.94 g cm^–3^ at 25 °C. The low-temperature DSC results (heating cycle) showed that there was no polymorph phase transition in the temperature range between 133 and 313 K of compound **1** (ESI Figure S2).

Compound **1** is slightly soluble in water at 25 °C (0.324 g/100 mL), and due to the relatively strong acidic character of permanganic acid,^29,31^ its saturated aqueous solution pH was 1.93. It is not soluble in benzene, toluene, chloroform, or carbon tetrachloride at all. Compound **1** does not dissolve in bromoform, but in a longer time, a reaction starts, indicated by gentle warming of the reaction mixture.

The solid compound **1** decomposed by standing in air at room temperature for several days with the formation of an X-ray amorphous decomposition product. The nature of amorphous (Fe,Mn)O_*x*_ phases containing iron was followed by Mössbauer spectroscopy (ESI Figure S3 and ESI Table S4), which showed that iron is in a trivalent state, and the decomposition involved a reduction of the permanganate ions and oxidation of the urea ligands in 5 days.

During the decomposition process, the amount of compound **1** continuously decreases (blue subspectrum), and correspondingly, the amount of iron(III) compounds formed during the decomposition reaction (green subspectrum) increases (ESI Figure S3). The dark purple color of the material gradually disappeared, and a brown color became more and more intense with time. According to its Mössbauer parameters, this intermediate degradation product contains iron(III), probably in an octahedral oxygen environment stabilized with the oxidation products of urea. The isomer shift and the quadrupole splitting correspond to ferrihydrite, although it could not be confirmed by XRD due to the amorphous character of the degradation product. The same decomposition was observed in the dark, so decomposition is not photochemical but can be attributed to the slow reaction between the permanganate ions and urea as a reducing ligand. Kótai et al. observed a similar degradation process involving the permanganate ion-mediated oxidation of the cation during storage in the case of other permanganate salts containing reducing cations.^29,32,33^ This decomposition reaction can be considered analogous to the decomposition of compound **1** in an aqueous solution, where it is much faster than in the solid state. Due to this, a large number of experiments were performed with no success of growing single crystals of compound **1** from aqueous solutions with gradual cooling and/or evaporation of the solvent from the saturated solutions between 0 and 25 °C. The decomposition reaction of compound **1** was faster in its aqueous solution than the growth rate of single crystals. Thus, the room-temperature saturated solution was quickly cooled to its freezing point in a deep fridge, and the primarily formed ice crystals were removed (these did not contain the dissolved salt) mechanically to make an oversaturated solution, which was left to crystallize in the cold. In this way, single crystals of appropriate quality and size were obtained. The calculated powder pattern of the complex is shown in ESI Figure S4.

#### Structural Features of Compound **1**

**Figure 1:**
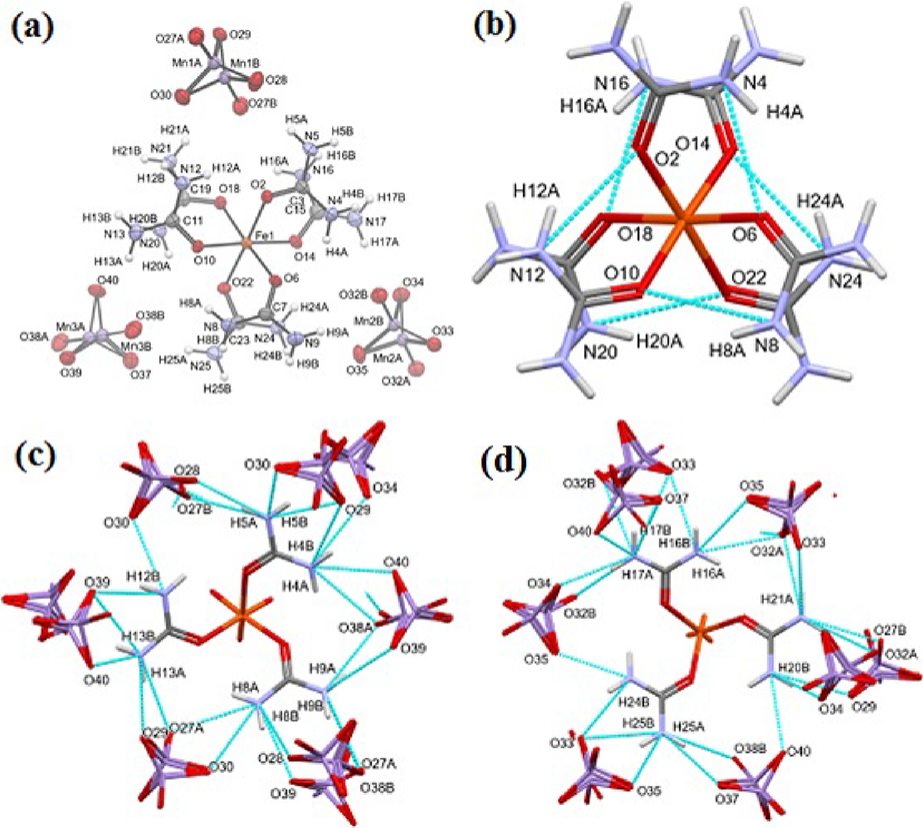
(a) Asymmetric unit of [hexakis(urea-*O*)iron(III)] tripermanganate (compound **1**). (Disordered atoms of the permanganate ions are labeled with “A” and “B”, respectively). (b–d) Hydrogen bonds in the structure. (b) Intramolecular hydrogen bonds of the [hexakis(urea-*O*)iron(III)] ion in compound **1**. (c, d) Intermolecular hydrogen bonds formed with the permanganate ions shown for the upper and lower half of the [hexakis(urea-*O*)iron(III)] ion in compound **1**.

The monoclinic dark purple needle-like single crystals decomposed rather quickly during long measurements, so numerous single-crystal X-ray diffraction measurements had to be performed. The final results (data collected from one single crystal) are summarized in ESI Table S3 and Tables S5–S11. The asymmetric unit of compound **1** contains a [hexakis(urea-*O*)iron(III)] complex cation and three permanganate anions in disordered orientations referred to as “A” and “B” (Figure 1a). The structure shows significant pseudosymmetry: the [hexakis(urea-*O*)iron(III)] complex cation, as well as the atomic positions of the disordered permanganate ions, fit *R*3̅*c* space group symmetry. However, the permanganate ions filling the channels between the [hexakis(urea-*O*)iron(III)] complex ions can only fit in one orientation (either “up” or “down”, respectively referred to as “A” and “B” orientations), which breaks the symmetry. There is a measurable difference in the occupation of the alternate orientations of the permanganate ions (94.19 and 5.81% for “A” and “B” orientations, respectively).

The urea molecules are placed around the iron(III) ion with an octahedral coordination geometry in propeller-like orientations. Thus, the complex shows helical chirality (ESI Figure S5). The permanganate salt of the [hexakis(urea-*O*)iron(III)] complex contains both enantiomers as it is crystallized in a centrosymmetric space group. The conformation of the complex is stabilized by internal hydrogen bonds (Figure 1b).

The permanganate anions form several hydrogen bonds between NH_2_ groups of the same urea molecules and make hydrogen-bonded bridges between urea molecules (Figure 1c,d). The hydrogen bond network also has pseudosymmetry, though not all intermolecular hydrogen bonds are present for all the urea ligands (hydrogen bonds are listed in ESI Table S5).

The shortest distances between the iron atoms in the structure are 7.037 and 6.667 Å, connecting neighboring complexes along unit cell axis. The iron atoms along the other two axes are separated more by the urea molecules, and the channels between them are filled by permanganate ions; their distances are 10.6–10.7 Å (ESI Figure S6).

**Figure 2:**
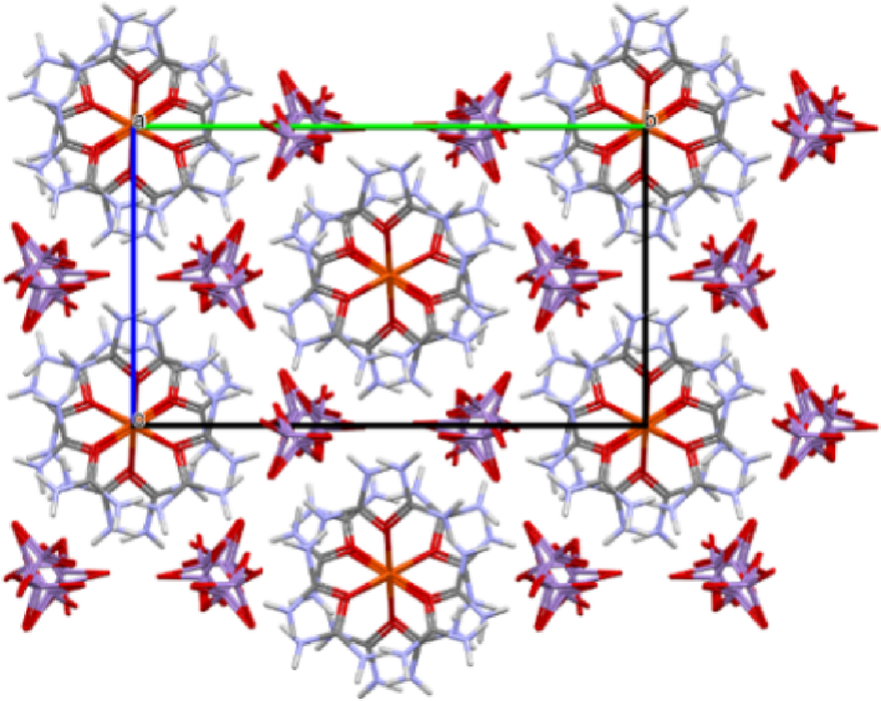
Crystal packing of the complex: view along unit cell axis *a*, which is the pseudo-3̅ axis.

The packing arrangement of the molecules is presented in Figure 2 and ESI Figure S7. The pseudo-3̅ axis is along unit cell axis *a*, which is parallel to these channels, and the urea propellers are always arranged along these axes. The disordered permanganate ions are arranged around further parallel pseudo-3̅ axes. Note that because of the pseudo-3̅ symmetry, the directions of the major component “A” are alternating “up” and “down” in neighboring channels.

The structure contains 0.8% of the potential solvent-accessible void, that is, 20.87 Å^3^ per unit cell (ESI Figure S8). The voids are formed between pairs of [hexakis(urea-*O*)iron(III)] complex ions with Fe–Fe distances of 7.037 Å. The Kitaigorodskii packing coefficient of the structure is 73.5%.

The pseudo-*R*3̅*c* symmetry (with cell volume of 3888.3 Å^3^) of the structure makes it similar to a group of hexacoordinated urea complexes found in the Cambridge Structural Database^34^ [Sc(urea-*O*)_6_]I_3_ (compound **2**),^35^ [Ti(urea-*O*)_6_]I_3_ (compound **3**),^36^ [Ti(urea-*O*)_6_](ClO_4_)_3_ (compound **4**),^37^ [Al(urea-*O*)_6_](ClO_4_)_3_ (compound **5**),^38^ [Fe(urea-*O*)_6_]I_2_ (compound **6**),^39^ ([Mn(urea-*O*)_6_](ClO_4_)_3_ (compound **7**)^40^ with *R*3̅*c* or *R*3̅ symmetry. In these structures, columns of the complex ions are surrounded with six columns of the anions; the conformation of the complexes is chiral propeller-like (ESI Figure S9).

#### Room-Temperature and Low-Temperature (Liquid N_2_) Vibrational Spectra of Compound **1**

The expected vibrational modes of compound **1** were estimated on the basis of correlation analysis (ESI Figures S10–S13). There are three and six crystallographically different permanganate ions and urea ligands present in the structure of compound **1**, respectively. Accordingly, three series of four normal modes (all are IR- and Raman-active) of permanganate bands are expected (ESI Figure S10), altogether 3 × 18 IR (*A*_u_ and *B*_u_) and 3 × 18 Raman active (*A*_g_ and *B*_g_) bands. The external modes of a permanganate ion, three translational and three rotational modes in *A*_u_ and *B*_u_, as well as in *A*_g_ and *B*_g_, respectively, are active. The number of all external vibrations is tripled (three permanganate ions; ESI Figure S11). As the degree of disorder is not very large (less than 6% of the occupancy), the disordered atoms (atoms labeled “B”) were not included in the calculation of either the internal or the external modes (if they were, that would once again double the number of modes, albeit the intensity would differ, ca. 1/20 of the bands belong to the other disordered positions).

The free urea ligand has eight atoms, which results in 24 degrees of freedom. There are six nongenuine motions (three translations and three rotations); thus, 18 internal modes of vibration (seven stretches, six in-plane bends, five out-of-plane bends) are to be considered.^41^ In compound **1**, the urea ligands (*C*_1_ site group and *C*_2h_ factor group) have 6 × 18 *A*_u_, 6 × 18 *B*_u_, 6 × 18 *A*_g_, and 6 × 18 *B*_g_ modes. Thus, all the vibrational modes are IR- and Raman-active. Altogether, 2 × 6 external modes (*T*_x_, *T*_y_, *T*_z_, *R*_x_, *R*_y_, and *R*_z_; three librations and three hindered rotations, respectively) can be expected (ESI Figure S12). Three (two *B*_u_ and one *A*_u_) of all hindered translations are due to lattice acoustic modes in the IR spectrum (ESI Figure S13). All vibrational modes are expected to appear as heavily overlapped bands (both due to crystallographically different moieties and correlation field splitting) in the spectra.

The details of spectroscopical analysis are given in the Supporting Information. The assignments of the vibrational modes are given in ESI Table S12. The room-temperature and liquid N_2_-temperature IR and Raman spectra are given in ESI Figures S14 and S15, respectively. The far-IR spectra are given in ESI Figure S16.

In compound **1**, the oxygen atom of urea acts as a donor atom, and due to the “pile-up” and “spill-over” effects, the C=O bond becomes longer and the C–N bonds become shorter than in the gaseous urea. The corresponding ν_CO_ and ν_CN_ stretching modes will shift to lower and higher wavenumbers, respectively. Due to the hydrogen bonding of the NH_2_ group, the N–H bonds become longer. The δ_as_(N–H) is coupled with ν_as_(C–N) and ρ_s_(NH_2_), whereas ν_as_(C–N) is coupled with δ_as_(N–H) and δ(C=O)).^41^ The ν(C=O) modes were expected between 1550 and 1500 cm^–1^. The wavenumber of ν(C=O) + δ_s_(N–H) combination bands and ν(C=O) bands give a possibility to determine the δ_s_(N–H) wavenumber positions (ESI Table S12). The band at 1503 cm^–1^ belongs to the ν(C=O) components due to the expected strong shifts of the ν(C=O) in compound **1** as compared to that of gaseous urea.^41^ The estimated maxima of δ_s_(N–H) (IR) is at δ_s_(N–H) ≤ 1682 cm^–1^, close to the δ_s_(N–H) value found in solid urea (1683 cm^–1^). The δ_s_(N–H) can unambiguously be assigned as a sharp band at 1679 and 1673 cm^–1^ in the Raman spectra at room temperature and 100 K, respectively.

#### UV Spectroscopy

The UV spectrum of compound **1** (ESI Figure S17), contains the usual visible CT bands of permanganate ion (^1^A_1_-^1^T_2_ (t_1_-2e)) at 498, 520, 544, and 567 nm, according to the purple color of the complex. In the UV range, we could not detect a band of ^1^A_1_-^1^T_2_ (3t_2_-2e) transition of permanganate at 251 nm. The high-spin oxygen-coordinated Fe^III^ complex as compound **1** contains an Fe^III^-ion with a ^6^S ground term. The ^6^S term cannot be split by a crystal field, and the transitions are spin and Laporte-forbidden transitions. Thus, the expected ^4^T_1g_(G) ← ^6^S, ^4^T_2g_(G) ← ^6^S, and the ^4^A_g_,^4^E_g_(G) ← ^6^S transitions of the [Fe(urea-*O*)_6_]^3+^ cation became too weak to detect. Since the urea ligands are bound to iron with various strengths, and as a consequence, there are various bond orders in the C=O group, in addition to regular n−π* and π–π* and LMCT bands, and residual bands appearing in the UV–vis spectra belong to these transitions.

#### Room-Temperature and Low-Temperature (Liquid N_2_) Mössbauer Spectra of Compound **1**

The Mössbauer spectra of compound **1** show only broad singlets assigned to only one iron site. This line shape is quite common for this type of complexes^42−44^ and can be attributed to the magnetic relaxation of spin–spin or spin–lattice origins. Considering that the shortest Fe–Fe distance in compound **1** is rather large (6.67 Å), spin–spin relaxation can certainly explain the line shape.

The spectra (ESI Figure S18) were evaluated by the Blume–Tjon two-state magnetic relaxation model offered by the Mösswinn code.^45^ The Mössbauer parameters given by this model can be separated into three groups. First, the basic parameters: the isomer shift (δ), the amplitude of the relaxing hyperfine magnetic field (*H*), and the internal line width (Γ). Second, instead of quadrupole splitting, intrinsic parameters directly characteristic to the electron density distribution around the nucleus, namely, the component *V*_zz_ of the electric field gradient tensor (EFG), where the direction *z* refers to the principal axis system of **H**, and the asymmetry parameter of the EFG (η) are calculated. Finally, the most specific Mössbauer parameter of this model is the jump-up rate of the relaxing magnetic field (forced to be equal to the jump-down rate because of two-state relaxation). This parameter is actually the base 10 logarithm of the jump-up frequency (*W*) from which the spin relaxation time may be calculated as π/*W*.

Depending on the initial values of the iteration, fitting the experimental Mössbauer data with the above model led to a very broad distribution of fitted parameters, in some cases, even to unphysical results. Thus, applying an approximation (e.g., disregarding the influence of the anions), values of the isomer shift (δ), η, and *V_zz_* obtained by density functional theory (DFT) calculations on the [hexakis(urea-*O*)iron(III)] cation were used, and some of them were fixed in the Mössbauer spectrum evaluations. The DFT-optimized structure parameters of the [hexakis(urea-*O*)iron(III)] cation are shown in ESI Table S13. The DFT calculation yielded δ = 0.573 mm/s, η = 0.232, and *V_zz_* = −0.762 × 10^21^ V m^–2^). Fixing all the three parameters resulted in unacceptable fits.

The experimental spectra were recorded at 298 and 80 K (parameters listed in ESI Table S14), and the evaluations resulted in isomer shifts of 0.412 and 0.501 mm s^–1^, respectively. In these evaluations, the DFT-calculated *V*_zz_ and η values were fixed assuming that these parameters are less sensitive to the effect of the permanganate anions located farther away from the central Fe^3+^ ions than the urea ligands. Furthermore, the EFG can be considered temperature independent in the 80–300 K range, while the isomer shift is affected by the second-order Doppler shift, unknown for this compound. The fitted isomer shifts confirm that compound **1** is a high-spin iron(III) complex. The experimental isomer shifts, obtained this way, and the DFT-calculated one (referring to 0 K) show reasonable agreement, taking into account the estimated second-order Doppler shift as well as the accuracy of the DFT calculations (previously, 0.05 mm/s mean that absolute error was found with respect to temperature-corrected experimental isomer shifts for a comprehensive data set of 66 iron complexes^46^).

The isomer shifts are smaller than those previously published for the urea complex containing Cl^–^ (0.58 and 0.60 mm s^–1^).^43^ This difference could be due to the hydrogen bonds (proved by the single-crystal X-ray structure) between the complex cation and the permanganate anion that influence the 3d density on the Fe^3+^ central ion. Nevertheless, one may keep in mind that determination of the isomer shift poses similar challenges in the quoted literature than in the present study.

The small η and *V*_zz_ (0.232 and −0.762 × 10^21^ V m^–2^) values can be attributed to the symmetrical arrangement of the urea ligands around the iron(III) center. Because of this, the electron distribution must be close to spherical around the central metal ion, and the anions and the non-cubic lattice seem to have little influence. The line widths are 0.626 and 0.875 mm s^–1^ at 295 and 80 K, respectively, and this is also a consequence of the relaxation.^47^

#### Selective Oxidations of Organic Substances with Compound **1**

There are numerous unexpected and selective organic oxidation reactions of pyridine, bipyridine, and ammonia-coordinated copper and silver permanganate complexes,^4,6,33^ but no information is available about complex permanganate salts of iron or other central atoms with urea as ligand. Therefore, using some simple target compounds, we tested the oxidation ability of compound **1** toward alcohols and diphenyl sulfide used as typical substrates in testing complex permanganate oxidants.^33^ The oxidation of substituted benzyl alcohols RC_6_H_4_CH_2_OH, R = H, 2-I, 2-NO_2_, 2-MeO, and 4-NO_2_) leads to benzaldehyde without benzoic acid formation at room temperature. The presence of *ortho*-substituents decreases the conversion of benzyl alcohols (Table 1). Especially, the electron-withdrawing nitro group has a strong negative influence on the formation of benzaldehyde (13% conversion was reached) at room temperature for 2-O_2_NC_6_H_4_CH_2_OH. After 4 h of reflux, however, the conversion of benzyl alcohol was completed, but the main product was not the expected benzaldehyde or benzoic acid^33^ but benzonitrile. The nitro group (electron-withdrawing) substitution of the phenyl ring in its *para*-position, however, increases the reactivity of benzyl alcohol.

**Table 1:** Oxidation of Benzyl Alcohols (R-C_6_H_4_CH_2_OH) into Benzaldehydes and Benzonitriles with Compound **1** in Benzene as Solvent

			conversion of benzyl alcohols		
RC_6_H_4_CH_2_OH	time (h)	temperature	RC_6_H_4_C(*O*)H	RC_6_H_4_CN	unconverted
R = H	2	25 °C	76		24
	2	reflux	93	7	
	4	reflux	68	32	
	4*	reflux	60	36	2
	11	reflux	21	77	
R = 2-I	2	25 °C	29		71
	2	reflux	79	3	18
	4	reflux	64	14	22
R = 2-NO_2_	2	25 °C	13		87
	4	reflux	37	63	
R = 2-MeO	2	25 °C	60		40
	2	reflux	62	11	24
	4	reflux	57	32	11
R = 4-NO_2_	2	25 °C	100	0	
	4	reflux		100	

**Scheme 1:**
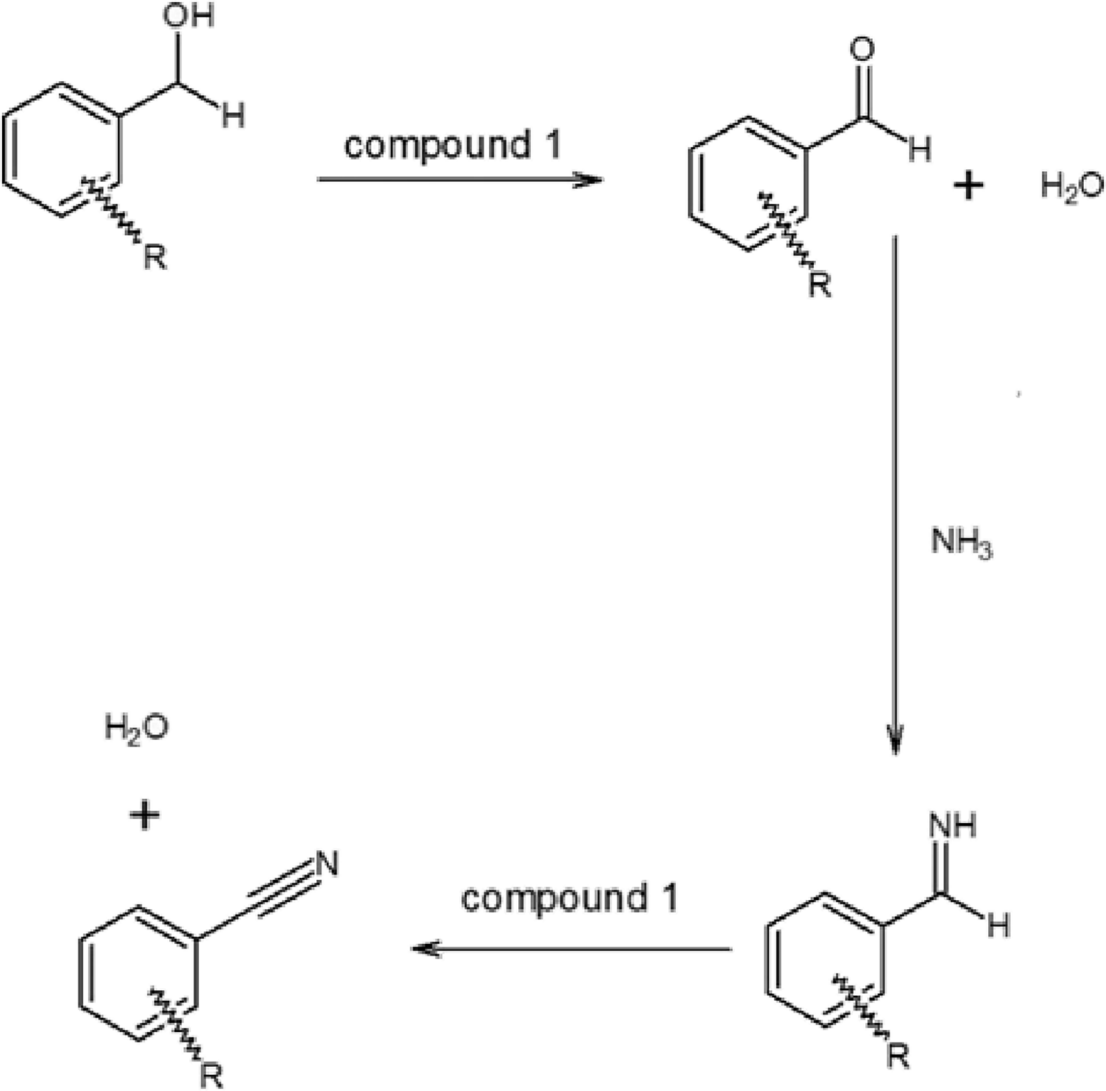
Plausible Mechanism of the Oxidation of Benzyl Alcohols with Compound **1** into Benzonitriles

It is known that benzonitrile can be formed in the oxidation of benzyl alcohol with ammonium permanganate,^48^ where the amount of ammonia needed for the ammoxidation reaction is rather limited to yield nitrile due to the stoichiometry (1:1) of NH_4_MnO_4_. In our case, the urea content of compound **1** is the source of ammonia. Thus, formally, 2 × 2 = 4 moles of ammonia per permanganate ion can be taken into consideration, supposing the complete hydrolysis of all urea. It is more than enough to complete the ammoxidation reaction, as was observed in the case of *p*-nitrobenzyl alcohol. The water source for urea hydrolysis is the oxidation of benzyl alcohol when 1 mol of water produces 1 mol of ammonia from urea, which, in principle, is enough for nitrile formation from 1 mol of benzaldehyde. It is obvious that the oxidation of benzyl alcohol is a key step in water formation and urea hydrolysis, but the oxidation of aldimine also results in the formation of water. Thus, the nitrile can be formed from benzaldehyde and ammonia, and the nitrile yield may not be higher than that arising from the conversion of benzyl alcohol into benzaldehyde. The more nitrile forms, the less benzaldehyde remains. In order to confirm the role of water in the reaction, we oxidized unsubstituted benzyl alcohol with compound **1** (Table 1), when 76% conversion was found at room temperature. At 2, 4, and 11 h of reflux, the conversion of benzyl alcohol became complete (without benzoic acid formation) with the formation of 93, 68, and 21% benzaldehyde and 7, 32, and 77% benzonitrile, respectively (Table 1). This shows that increasing the reaction time increases the yield of benzonitrile. When a small amount of water (250 μL) was added at 4 h reflux, the amount of benzaldehyde decreased and the amount of benzonitrile increased. The presence of water, however, decreased the conversion of benzyl alcohol into aldehyde, and 2% benzoic acid formed. The possible reaction route of this multistage one-pot reaction is summarized in Scheme 1.

Secondary alcohols, e.g., 2-octanol can also be oxidized into an oxo-compound, 2-octanone, at a conversion rate of 15, 52, or 67% at room temperature in 2 h and at reflux temperature in 2 or 4 h, respectively. Diphenyl sulfide is oxidized with compound **1** with less selectivity. Conversion was 48 and 62% in 2 h at room temperature and at reflux temperature, respectively, with increasing diphenyl sulfoxide conversion from 2 to 9% and diphenyl sulfone conversion from 36 to 43% on refluxing. A small amount of diphenyl disulfide and thianthrene were also detected (in ∼1.3% at room temperature and 1.1% at reflux temperature).

#### Heat-Induced Decomposition Reactions of Compound **1**

The thermal decomposition of compound **1** was followed by TG-MS and DSC methods in inert (Ar or N_2_) and oxidative (air or O_2_) atmospheres (ESI Figure S19–S22). The TG-DTG data show multistep decomposition processes, and the exothermic decomposition starts at the same temperature (94 °C) in N_2_ and air atmospheres. This shows that oxygen has no role in the initiation of the decomposition reaction.

The reaction heats in the inert and in an oxygen atmosphere, however, are different (ESI Figures S21 and S22). This indicates that aerial oxygen is involved in completing the first stage of the oxidation step, which cannot be completed in an inert atmosphere due to the oxygen deficit of compound **1** (12 oxygen atoms of three permanganate anions are not enough to complete the oxidation of six urea ligands because depending on the oxygen content of residues containing metal, on the average, less than 2 oxygen atoms can be used to oxidize one urea molecule.

#### Evolution of Metal-Containing Phases in the Decomposition Products

**Figure 3:**
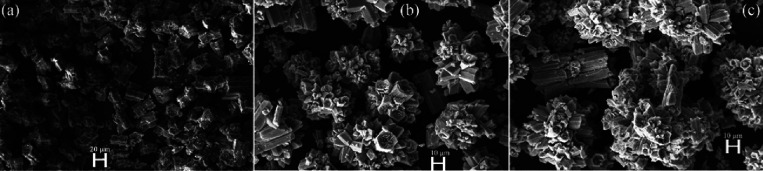
SEM image of (a) compound **1** and its decomposition product formed from compound **1** after heating at (b) 350 °C and (c) 800 °C in air.

The decomposition temperature of compound **1** (*T* = 94 °C) is lower than that of [Fe(urea-*O*)_6_](NO_3_)_3_ (compound **9**, *T*_dec_ = 155 °C)^27^ or of urea itself (*T*_dec_ = 135 °C).^49^ In contrast to the thermal decomposition process of compound **9**, which starts with an endothermic urea ligand loss step (followed by the oxidation of liberated urea with the nitrate ions making the process exothermic^27,28,50,51^), no endothermic urea ligand loss is observed during the thermal decomposition process of compound **1** (ESI Figures S21 and S22). The lower decomposition temperature of compound **1** compared to that of the hexa(urea-*O*)iron(III) complex cation^27^ in its salts with non-reducing anions shows that the initial step of the thermal decomposition process of compound **1** is a solid-phase quasi-intramolecular redox reaction between the coordinated urea ligand and permanganate anions. With scanning electron microscopic measurements, we were able to prove that the redox reaction takes place inside the lattice structure since the morphology of compound **1** did not change during the heat treatment (Figure 3). The microsized crystals kept their trigonal shape even at 800 °C, but they aggregated together and formed rose bush-like microcrystals. After grinding the material that we obtained at 800 °C in air, an inner porous structure appeared (ESI Figure S23).

**Figure 4:**
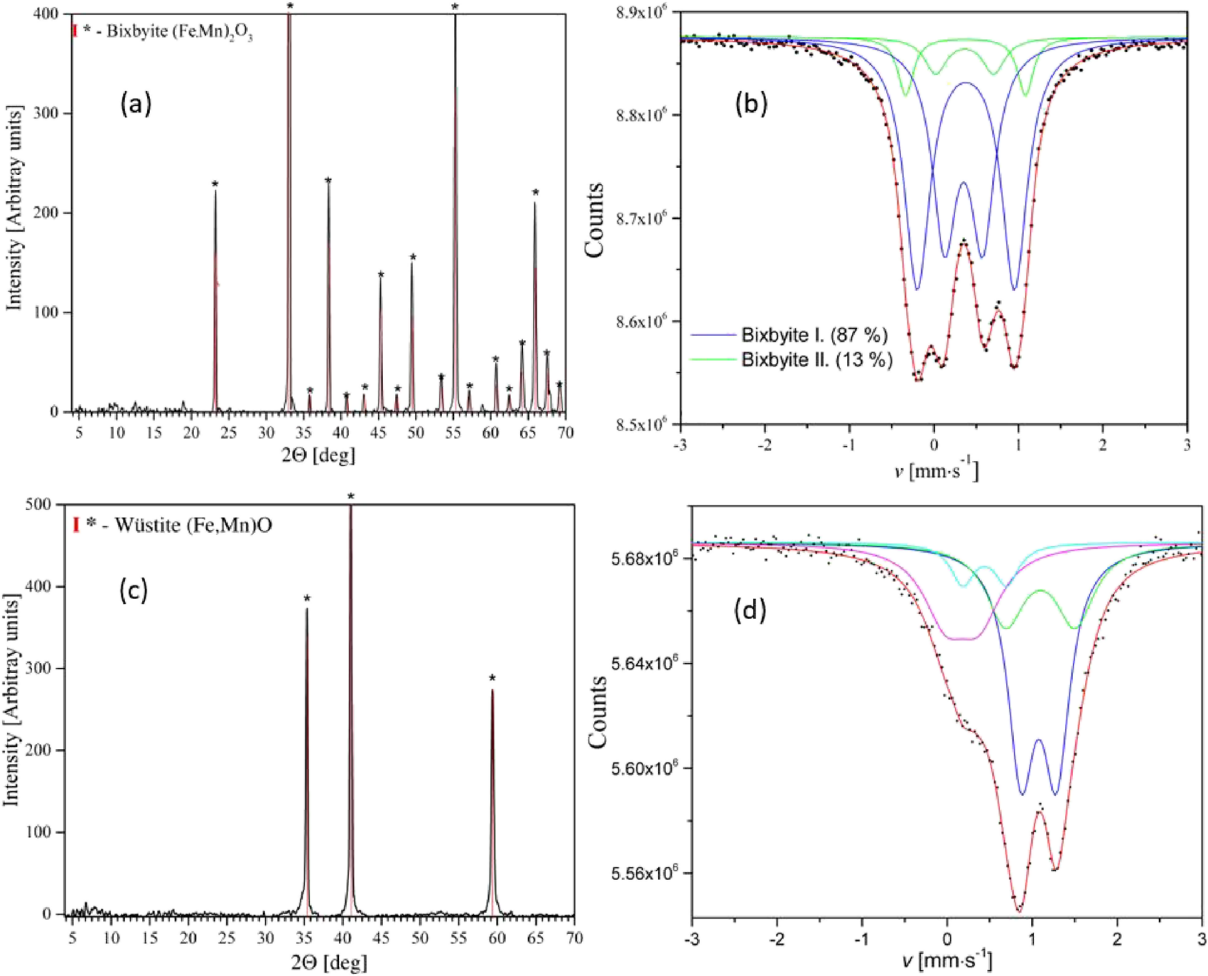
Powder XRD (a, c) and Mössbauer spectra (b, d) of the decomposition products that formed from compound 1 after heating at 800 °C in air (a, b) and in inert atmosphere (c, d).

The overall mass decrease resulting in 41.0 and 37.3% residue in air and in inert atmosphere shows that the final decomposition products that formed at 800 °C are (Fe,Mn)_2_O_3_ and (Fe,Mn)O, respectively, with 1:3 overall Fe to Mn stoichiometry. This was confirmed by XRD and Mössbauer spectroscopy (Figure 4 and ESI Tables S15 and S16).

The air and inert atmosphere decomposition processes resulted in X-ray-amorphous products below 350 and 800 °C, respectively (ESI Figures S24 and S25).

**Figure 5:**
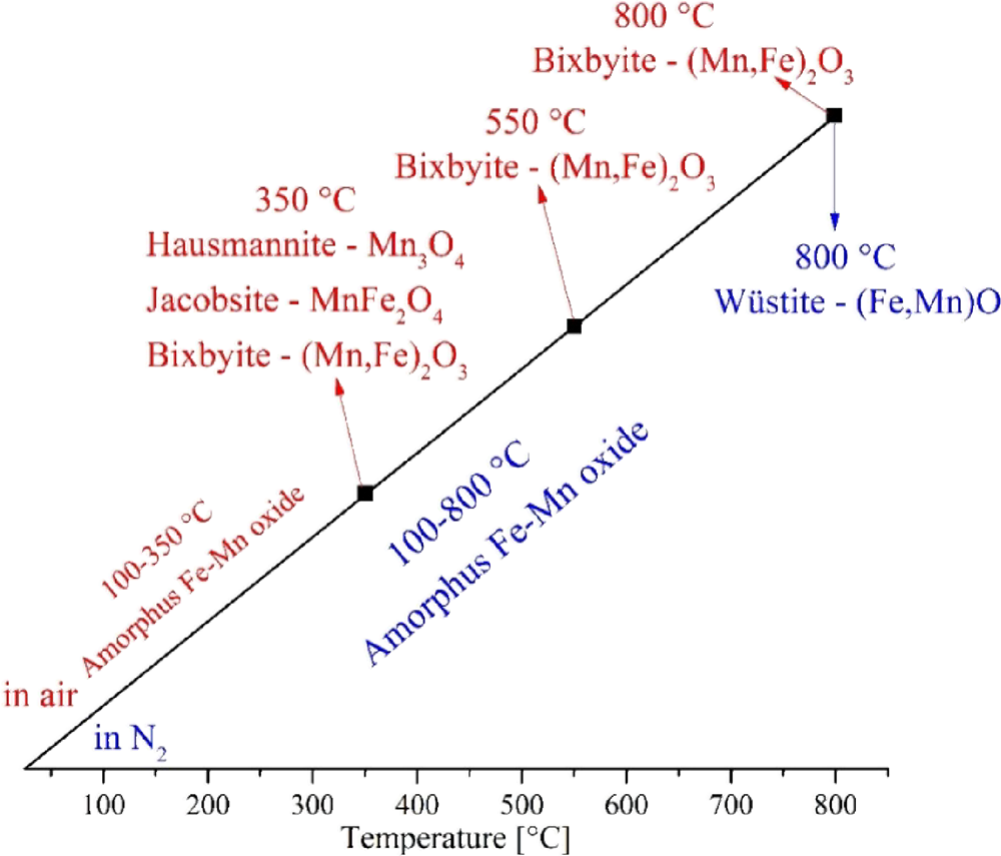
Main phases found during the thermal decomposition of compound **1** in air (red) and in N_2_ (blue).

The decomposition intermediates found upon heating in air at 350 °C were hausmannite (tetragonal spinel, Mn^II^Mn^III^_2_O_4_), non-stoichiometric jacobsite (cubic spinel, Mn^II^Fe^III^_2_O_4_), and bixbyite (Mn^III^,Fe^III^)_2_O_3_ (Figure S26). The main phases found during the thermal decomposition of compound **1** are summarized in Figure 5. When further heated in air, hausmannite and iron-rich jacobsite reacted with oxygen, and their Mn^II^ content was oxidized into Mn^III^ with the formation of bixbyite ((Fe^III^,Mn^III^)_2_O_3_) above 350 °C. The Mössbauer spectra of the intermediate phases formed at 350 °C in air (Figure S26b) showed that the distribution of iron between jacobsite and bixbyite phases is 53 and 47%, respectively (ESI Table S17). No Fe^II^ is present in these samples; thus, the divalent metal in the spinel structure can only be Mn^II^. Due to stoichiometry, not only Fe^III^ but Mn^III^ should also be present in the jacobsite. Fe^III^ ions favor the octahedral, whereas Mn^II^ ions favor the tetrahedral sites of the spinel lattice. The iron distribution between the octahedral and tetrahedral spinel sites was found to be 70 and 30%, respectively (ESI Table S17). In regular jacobsite, the Mössbauer spectrum contains two sextets assigned to tetrahedral and octahedral lattice sites. The isomer shifts of iron at these sites are practically identical, which means that the 3d electrons of iron are highly delocalized. For the same reason, the hyperfine magnetic fields were found to be also high (44 and 49 T) and close to each other.^52,53^ In our sample where the jacobsite formed by the decomposition of compound **1**, we were able to decompose the Mössbauer spectrum (Figure 26b) into three sextets, which correspond to the tetrahedral and octahedral Fe^III^ positions in jacobsite, with isomer shifts of 0.25, 0.37, and 0.39 mm/s and anomalously low hyperfine fields of 24.4, 44.0, and 38.5 T, respectively, and all quadrupole shifts close to zero (ESI Table S17). One can assume that due to the excess of Mn (relative to the stoichiometry of jacobsite), this jacobsite contains a substantial amount of Mn^II^ and Mn^III^ substituted at Fe^III^ sites as well as oxygen vacancies. This breaks the delocalization of the Fe3d electrons, resulting in significantly lower magnetic hyperfine fields and different isomer shifts. The lowest isomer shift can be attributed to a localized tetrahedral Fe^III^, while the other two sextets must be due to octahedral sites with different distortions or different valences of Mn neighbors. Thus, the formula of the jacobsite formed in this experiment can be written as (Mn^II^, M^T-4^(M,Mn^II^)^OC-6^_2_O_4_ (M = Fe^III^, Mn^III^), which differs from the expected Mn^II^Fe^III^_2_O_4_ formula.

**Figure 6:**
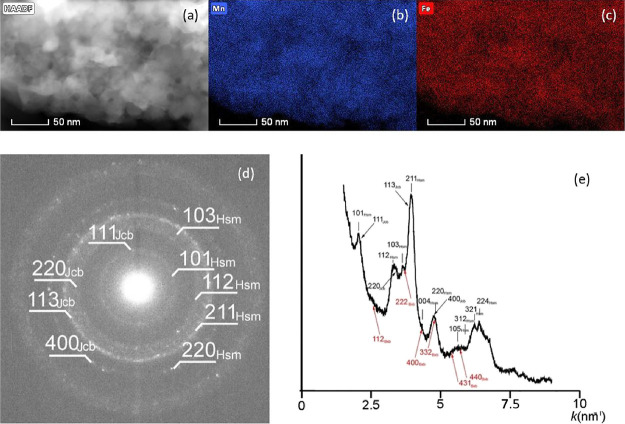
(a) High-angle annular dark field scanning TEM (HAADF-STEM) image and (b) Mn and (c) Fe maps of the sample prepared at 350 °C; (d) SAED pattern (e) and its circularly integrated distribution showing hausmannite (Hsm) and jacobsite (Jcb). Red arrows point to bixbyite (Bxb).

A TEM investigation of the sample prepared at 350 °C in air showed that the size of particles was between 20 and 40 nm and they had uniform distribution of Mn and Fe (Figure 6a,b,c). The selected-area electron diffraction pattern of these nanoparticles revealed rings that spread across several pixels and suggested the occurrence of multiple structures. In particular, distributions calculated from the SAED pattern following the method described by Lábár^54^ and using Process Diffraction software^55^ show broad peaks that correspond to hausmannite, jacobsite, and some bixbyite (Figure 6d,e). Based on the elemental maps (Figure 6a,b,c), the Mn and Fe distributions in the studied grains are fairly uniform.

**Figure 7:**
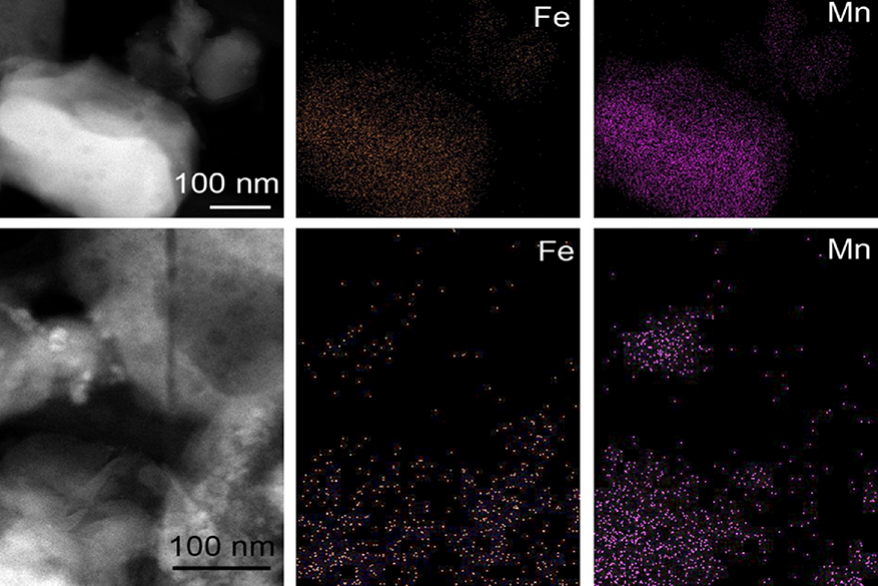
HAADF-STEM images and elemental maps of a sample heat-treated at 800 °C.

The Mössbauer analysis revealed that the oxidation of the jacobsite phase or its reaction with hausmannite during heating in air to 800 °C resulted in two kinds (type I and II) of bixbyite with various Fe^III^ and Mn^III^ distributions.^56,57^ The ratio of bixbyite I and II was found to be 13:87 (Figure 4b and ESI Table S15). A bixbyite phase formed at 350 °C contained 41 and 59% of its iron content in octahedral and tetrahedral environments, respectively (ESI Table S17). This ratio was found to be 43%/57% and 47%/53% for type I and II bixbyite formed at 800 °C, respectively (ESI Table S15). The HAADF STEM images show particles of 200–500 nm, which are either rich in Fe (and poor in Mn) or poor in Fe (and rich in Mn) (Figure 7).

**Figure 8:**
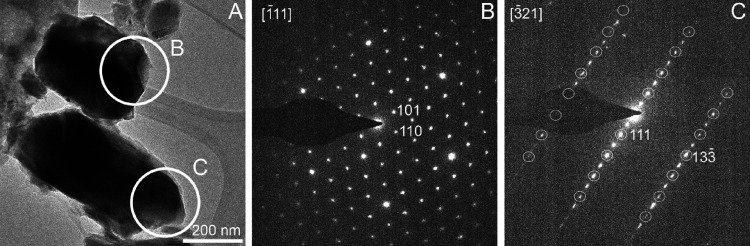
(A) TEM image and (B, C) SAED patterns of two bixbyite types. White circles in (C) mark ordinary (type I) bixbyite reflections.

TEM images and SAED patterns of the chemically different grains confirmed the presence of two bixbyite types (Figure 8). The SAED pattern taken from selected grains resembles the characteristic diffraction features of ordinary bixbyite with *a* = 9.4 A and space group *Ia*3. However, the SAED pattern of some grains shows reflections that indicate a second bixbyite structure type. In particular, only half of the reflections (white circles) of the SAED pattern shown in C can be indexed with the ordinary bixbyite. The extra reflections along only <111> suggest that the second bixbyite type is crystallographically associated with ordinary bixbyite.

Based on these diffractions, one can assume that a surplus arrangement of the bixbyite exists in the [111] direction, resulting in a double-size superlattice with a cell parameter *a* = 2*d[111]. The cell angles may be distorted, resulting in a monoclinic or even triclinic cell arrangement as well. In sample *a*, Fe and Mn are distributed in almost the same way. In sample *b*, there are differences between Fe and Mn content, showing the accumulation of each element in the (Fe,Mn)_2_O_3_ phase in various parts of the grain.

When the sample containing the bixbyite phases was further heated (produced at 800 °C in 0.5 h in air; bixbyite I to bixbyite II ratio = 87:13), the bixbyite I (supercell, heterogeneous) phase was gradually converted into bixbyite II (bixbyite I to II ratio became 44:56). The ratio of iron in tetrahedral to octahedral sites changed from 57:43 and 53:47 to 60:40 and 63:37 for bixbyite type I and II, respectively (ESI Figure S27 and ESI Table S15).

The amorphous sample made in an inert atmosphere at 350 °C was converted into a crystalline phase during storage in air for a week (ESI Figure S28). The in situ powder XRD of this amorphous sample showed that the crystallization temperature is 550 °C (ESI Figure S29). During crystallization, only the bixbyite I phase (distorted) formed, with 27 and 73% octahedral and tetrahedral occupancy of iron(III), respectively (ESI Figure S30 and ESI Table S15). During the transformation of bixbyite I into bixbyite II by heating for different durations at 800 °C, not only the bixbyite I to bixbyite II ratio but also the distribution of iron(III) between the octahedral and the tetrahedral sites changed (ESI Table S15). These results show that the temperature and the time of heat treatment have a crucial effect on the transformation from bixbyite I to bixbyite II. The rearrangement of the phase with inhomogeneity in Fe and Mn distribution causes entropy increasing; thus, the process is kinetically controlled and longer time and higher temperature (increased diffusion rate) help the recrystallization process.

#### Evaluation of Fe^III^ Reduction Processes

**Figure 9:**
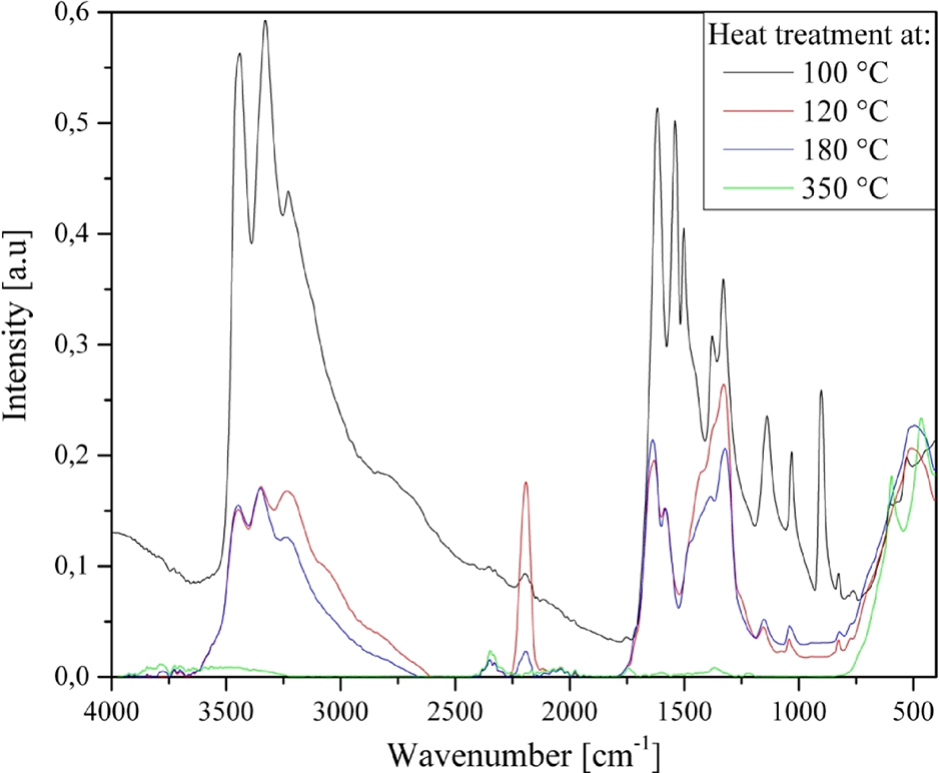
IR spectra of the decomposition residues.

The formation of wuestite containing formally Fe^II^ and Mn^II^ shows the reduction of permanganate and iron(III) into a divalent state (Figure 9c,d and ESI Table S16). We were not able to isolate any crystalline intermediate (ESI Figure S25), but the Mössbauer spectrum of the amorphous material formed at 350 °C only showed the presence of phases containing iron(III) (ESI Figure S31 and ESI Table S18). However, the question arises: as there is no Fe^II^, what is the reducing agent that converts Fe^III^ into Fe^II^ above 350 °C?

**Scheme 2:**
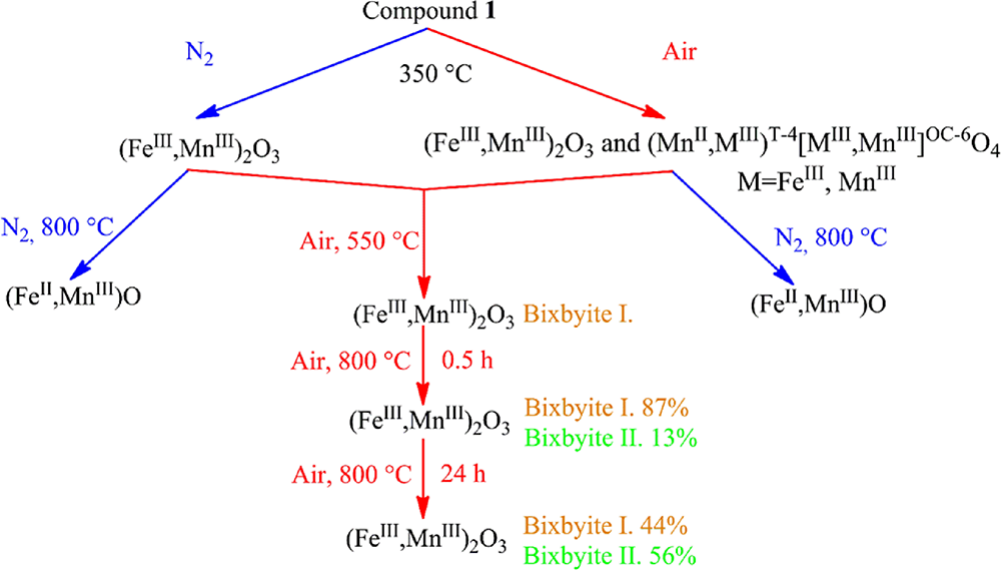
Transformation of the Main Phases Found during the Thermal Decomposition of Compound **1**

Both intermediates formed in air (A) and in N_2_ (B) at 350 °C contain iron(III) compounds only, with an overall Fe to Mn stoichiometry of 1:3. However, some residual organic degradation products (carbon-like) must be present in the sample obtained in N_2_ atmosphere, while in the samples treated in air, these organic residues could be combusted. To reveal more details of the decomposition process, we further heated the samples made in air (A) and in N_2_ (B) separately in N_2_ and in air, respectively. Surprisingly, the phase composition of the end-product depended only on the atmosphere used in this step (air or N_2_) and did not depend on which intermediate (A or B) was used. Namely, if the samples prepared in either air or N_2_ at 350 °C (A or B) were heated in N_2_ to 800 °C, only wuestite (Fe^II^,Mn^II^)O formed, whereas heating them to 800 °C in air produced only bixbyite (Fe^III^,Mn^III^)_2_O_3_. The results are summarized in Scheme 2.

The reduction processes of Fe^III^ and Mn^III^ into Fe^II^ and Mn^II^ occurs between 350 and 800 °C in a N_2_ atmosphere. The reducing partners are the residual organic materials (intermediate A), or the Mn^II^ content of the jacobsite (intermediate B without organic residues). In the case of intermediate A, CO_2_, N_2_/CO (*m*/*z* = 28), and NO form between 400 and 600 °C (ESI Figure S32). The presence of NO, CO_2_, and N_2_/CO gives direct evidence of a redox process involving organic residues. We could not find carbonate or nitrate residues by IR in the intermediate that formed at 350 °C (Figure 9); thus, no basic carbonates or nitrates existed as sources of CO_2_ or NO, respectively.

**Figure 10:**
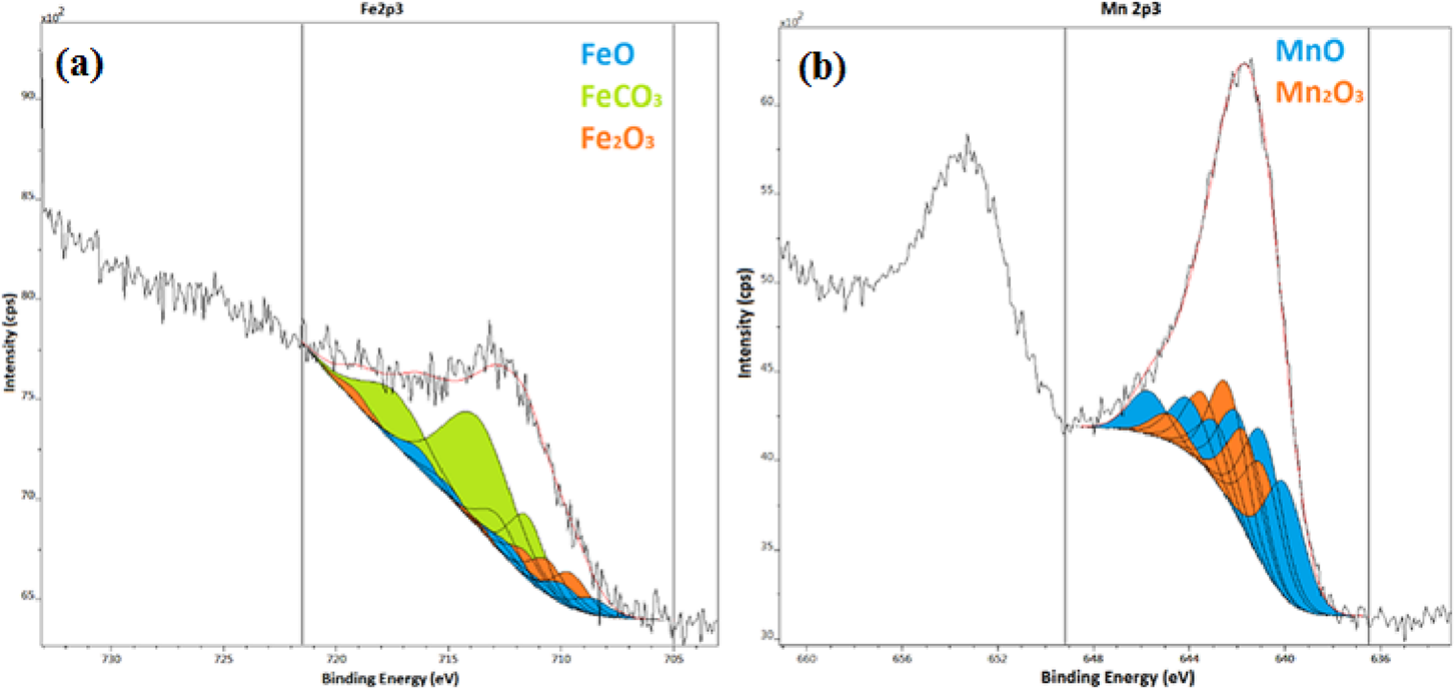
Fitting of the iron and manganese 2p_3/2_ peaks by multiplet lines of (a) FeO or FeCO_3_ and (b) MnO and Mn_2_O_3_, respectively.

We analyzed the Fe and Mn XPS spectra of the sample made in N_2_ atmosphere at 800 °C using only the 2p_3/2_ Fe and 2p_3/2_ Mn lines (Figure 10, ESI Table S19). Figure 10a shows the fitting of the Fe 2p_3/2_ line; multiplet peaks of FeO are denoted by filled-up peaks. Figure 10b shows the fitting of the Mn 2p_3/2_ line; multiplet peaks of MnO are denoted by filled-up peaks. The spectral data were fitted on the basis of the peak shapes with the use of the high-resolution data given in ref (58). In the case of both the iron and manganese spectra, complex multiplet splitting due to the unpaired electrons makes the interpretation of the spectra difficult. In these cases, when a core electron vacancy is formed by photoionization, coupling is possible between the unpaired electron in the core and the unpaired outer shell electron. This effect will create several final states and, as a result, multiplet peaks in the photoelectron spectrum. The Fe 2p_3/2_ and Mn 2p_3/2_ spectral data were fitted on the basis of the peak shapes with the use of the high-resolution data.^58^ The broad peaks of the photoelectron spectra could be fitted only with an envelope peak of certain oxides and iron(II) carbonate. On the basis of multiplet peak fitting, 16% of the surface iron can be estimated to be in the FeO state, 64% was fitted as FeCO_3_, and 20% was fitted as Fe_2_O_3_. Approximately 40% of the manganese was fitted as MnO, and 60% was fitted as Mn_2_O_3_. Since with Mossbauer spectroscopy, about 70% of the iron is in the Fe^II^ form (ESI Table S16), the appearance of iron carbonate and Mn_2_O_3_ may be attributed to the surface carbonation of FeO and aerial oxidation of MnO, respectively.

In the lack of organic residues in the aerial sample made at 350 °C, iron(III) is reduced to iron(II) by the manganese(II) content of jacobsite. Mn^III^_2_O_3_ is stable up to 942 °C;^4^ thus, Mn(II) can only be formed via a redox reaction at 800 °C. In contrast to the transition-metal permanganate complexes containing pyridine and ammonia ligand, which generally resulted in spinel-type (MMn_2_O_4_, M = transition metal) decomposition products,^4,8−10^ the urea as reducing ligand resulted in (Fe,Mn)_2_O_3_ (bixbyite) and (Fe,Mn)O (wuestite) during the decomposition of compound **1** in oxygen-containing and inert atmospheres, respectively.

#### Transformation of Urea Ligands in Compound **1** during Its Decomposition

We performed a TG-MS study to follow the gases evolving as the oxidation products of urea under argon atmosphere, i.e., to follow the *m*/*z* = 28 (N_2_) signals (ESI Figures S32 and S34). The main thermal decomposition process takes place between 94 and 120 °C and consists of three partly coinciding intervals. The initial reaction starts around 94 °C as a simple redox reaction of urea with the formation of H_2_O, CO_2_, CO/N_2_, and NO (ESI Figures S32 and S34). No ammonia is formed. This combustion process is much more intensive in air. The difference between the molar reaction heats in an inert gas (which involves the oxidation reaction by permanganate only) and air (which involves both the permanganate and aerial oxidation reactions) cannot be used to calculate the reaction heat of permanganate oxidation because different reaction products (e.g., NO_2_ from a part of NO) are produced. The ν_as_(Mn-O) permanganate ion peak (∼900 cm^–1^) disappears from the IR spectrum of the decomposition residue until 120 °C (Figure 9). The second decomposition process above 120 °C results in NH_3_, CO_2_, and H_2_O without the formation of NO (ESI Figure S32).

Thus, the second step of the main decomposition reaction consists of transformations of urea ligands with NH_3_ and H_2_O elimination without any redox reaction between the urea and the permanganate decomposition products (Fe/Mn oxides). The possible ammonia elimination reactions are the following:
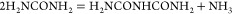
E1
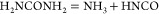
E2

The IR spectra of the decomposition residue formed at 120 °C contain ν(NH) bands and mixed-amide ν(CONH) bands, which did not change when heated to 180 °C, so these materials are stable even at 180 °C but decompose in air between 180 and 350 °C (ESI Figure S33). This is in accordance with the formation of biuret or biuret-like condensation products (eq.E1). A carbon–nitrogen multiply bonded intermediate (coordinated or non-coordinated) band appears at ν = 2192 cm^–1^, which may belong to isocyanic acid that forms according to eq E2.

The water elimination reactions may proceed as follows:
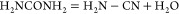
W1
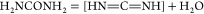
W2It is challenging to distinguish the two isomers (cyanamide and carbodiimide) or their hydrolysis products (cyanic/isocyanic acid) by IR because the C–N multiple bond modes are close to each other or cover the amide band region of the biuret. The masses of parent ions and their possible fragments are strongly coinciding (ESI Figure 33b). The peak at *m*/*z* = 43 may be either cyanic or isocyanic acid, and the weak peak at *m*/*z* = 42 may be either peaks of NCO or H_2_NCN. The hydrolysis reactions provide ammonia and cyanic/isocyanic acids (eqs H1 and H2), or the further hydrolysis of HOCN/HNCO results in NH_3_ and CO_2_. Carp et al.^27^ detected the presence of HNCO by GC-IR among the decomposition products of [Fe(urea-*O*)_6_](NO_3_)_3_ (compound **9**) due to the oxidation of urea by nitrate ions of compound **9**. Thus, the most probable intermediate (forming above 100 °C and decomposing below 180 °C) is a free or coordinated isocyanic acid.
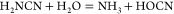
H1
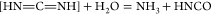
H2

The third part of the main decomposition step in an inert atmosphere shows the presence of NO as an evidence of a further redox reaction. The higher intensity of the *m*/*z* = 17 peak than the intensity of the *m*/*z* = 18 peaks shows that ammonia is present. The intensity of the *m*/*z* = 16 peak (NH_2_^+^ or O^+^) is almost equal to the intensity of the *m*/*z* = 17 signal; thus, the *m*/*z* = 16 signal consists of more than one component (ESI Figure S34).^12^ The presence of *m*/*z* = 44 (CO_2_ or N_2_O), and *m*/*z* = 28 (CO/N_2_), *m*/*z* = 18 (H_2_O), and *m*/*z* = 30 (NO) peaks show an oxidation reaction between the organic components and oxides that formed in the first permanganate-mediated redox reactions.

In an inert atmosphere, the TG-MS peaks found at higher temperatures up to 600 °C contain NO, H_2_O, N_2_, and CO_2_ signals, which coincide with each other and show the presence of redox reactions between the intermediate oxide phases and organic degradation residues. These reactions are responsible for the reduction of Fe^III^ and Mn^III^ to their divalent state.

#### Preliminary Results on the Catalytic Effect of the Thermal Decomposition Products of Compound **1** in CO_2_ Hydrogenation

Five (Fe,Mn) oxide samples prepared in air (100, 120, 180, 350, and 550 °C) and one sample prepared in an inert atmosphere (350 °C) were studied as catalysts in the hydrogenation of CO_2_ at H_2_ to CO_2_ ratio 3:1 at a pressure of 20 bar between 175 and 550 °C for 4 h. The hydrogenation reaction resulted in CO and CH_4_, together with a small amount of ethane. The results are summarized in ESI Table 20.

CO_2_ conversion starts at around 350 °C, and above this temperature, it was found to be between 50 and 60% in all cases (ESI Figure S35). The samples prepared below these temperatures obviously transform chemically even before reaching 350 °C. The highest conversion (57.6%) is reached with the sample made at 350 °C. However, hydrocarbon selectivity was the highest with the sample made at 180 °C (the presence of H_2_ may play a role in the change of valence distribution in species containing Fe and Mn when the sample is heated until 350 °C). An overall hydrocarbon selectivity of 39.3% was reached, and a small amount (0.9%) of propane was also detected. No propane formed with other samples.

These preliminary results show a promising possibility of making catalysts for CO_2_ hydrogenation by manipulating the active centers with activation of samples made at 180 °C in various (reductive) atmospheres. The general isomorphism between the [hexakis(urea)metal(III)] permanganates and perchlorates^29,59−61^ makes it possible to prepare [(Fe,M^III^)(urea)_6_][(XO_4_)_3_ (M^III^ = Cr, Al, X = Cl, Mn) co-crystals and decompose them into Fe_2_O_3_-, Al_2_O_3_-, or Cr_2_O_3_-supported MnO_*x*_ catalysts. Similarly, doping/co-doping with hexakis(urea)rhodium(III) or iridium(III) perchlorate or permanganate complexes can give a series of the above-mentioned M_2_O_3_ oxide-supported Rh, Ir, Mn-Rh or Mn-Ir catalyst candidates for CO_2_ hydrogenation.

### Experimental Section

Chemical-grade iron(III) nitrate nonahydrate, urea, nitric acid, and 40% aq sodium permanganate, ethanol, 70% aq perchloric acid, 2,4-dinitrophenylhydrazine, ethanol, benzene, benzyl alcohol, 2-iodobenzyl alcohol, 2-nitrobenzyl alcohol, 2-methoxybenzyl alcohol, 4-nitrobenzyl alcohol, 2-octanol, and diphenyl sulfide were supplied by Deuton-X Ltd., Érd, Hungary.

Iron(III) nitrate nonahydrate (8.08 g, 0.02 mol) and urea (7.20 g, 0.12 mol) were dissolved in 9 mL of water. The orange-colored solution was mixed with 16.9 mL of 40% aq NaMnO_4_ solution (0.06 mol) under stirring. The solid part was separated by filtration on a G4 glass filter, washed with a copious amount of cold (0 °C) water, and dried overnight in a desiccator filled with P_2_O_5_. The [Fe(urea-*O*)_6_](MnO_4_)_3_ formed as a blackish-purple material (9.55 g) with a yield of 62%. The iron and manganese contents were measured by ICP after dissolution in water acidified by nitric acid, whereas the CHN content was measured after ignition as CO_2_, H_2_O, and N_2_, which showed that compound **1** contains six urea and three manganese units against one iron. The complex is anhydrous. Elemental analysis data calculated for [Fe(urea-*O*)_6_](MnO_4_)_3_) are C = 9.70, H = 2.76, N = 21.64, Fe = 7.60, and Mn = 21.06%; found: C = 9.31, H = 3.11, N = 21.73, Fe = 7.22, and Mn = 21.32%.

Pycnometric density was determined at 25 °C in bromoform:chloroform (1:1.43 mixture, *d* = 1.86 g/mL).

The CHN analysis of compound **1** was performed on a Carlo Erba 1106 instrument.

The iron and manganese content of the studied samples were determined by atomic emission spectroscopy (Spectro Genesis ICP-OES) with the use of a multielement Merck standard solution for calibration.

X-ray powder diffraction patterns were recorded with a Bragg–Brentano parafocusing goniometer (Philips PW-1050) equipped with a Cu anode (40 kV, 35 mA, supplied with a secondary beam graphite monochromator and a proportional counter). Scans were recorded in step mode, and the diffraction patterns were evaluated by full profile fitting technique.

A single crystal of 0.3 × 0.07 × 0.06 mm was selected for data collection and mounted on a loop. X-ray diffraction data were collected on a Rigaku XtaLAB Synergy R diffractometer equipped with a PhotonJet-R rotating anode source (Cu K_α_ radiation), confocal mirrors as a monochromator, and a Hypix-6000HE detector. Data collection and data reduction were carried out using the CrysAlisPro v.1.171.40.68a program.^62^ The crystal was kept at 100.00(10) K during data collection. The resolution range was restricted by diffractometer geometry to 0.8 Å. Crystals showed significant X-ray radiation sensitivity, so the completeness of the data set is 97%. Numerical absorption correction was applied to the data. Using Olex2,^63^ the structure was solved with the SHELXT^64^ structure solution program using intrinsic phasing and refined with the SHELXL^64^ refinement package. The non-hydrogen atomic positions have been refined by anisotropic full-matrix least-squares refinement. Hydrogen atoms were generated using geometric evidence and refined using the riding model with N–H bond lengths fixed to 0.88 Å. Fixed *U*_iso_ parameters for H atoms were applied at 1.2 times of N atoms in all N(H,H) groups. The permanganate anions show alternative orientations: a common occupancy value was refined for all orientations’ *A*′ and for all orientations’ *B*′ as mixed orientations of the three permanganate ions cannot fit in the channels. Restrained distances were applied as follows: within each permanganate ion, Mn–O distances (with σ of 0.02) and O–O distances, which involve the disordered atoms (with σ of 0.04). *U*_aniso_ constraints for the alternate atomic positions were applied, respectively. The number of restraints is 93. Residual electron density peak and hole of 1.63 and −0.92 eÅ^–3^ around metal atoms remained in the structure, which is acceptable for inorganic crystals. The Mercury^65^ program was used for structure analysis and preparation of the figures. Validation was carried out using PLATON.^66^ Please note that the validation report contains level B alerts indicating possible higher symmetry caused by the pseudosymmetry present in the crystal structure. This pseudosymmetry is discussed in the Results and Discussion section. Crystallographic data were deposited in the Cambridge Structural Database;^34^ CCDC 2181272 contains the supplementary crystallographic data for this paper. These data can be obtained free of charge from The Cambridge Crystallographic Data Centre via www.ccdc.cam.ac.uk/structures. Crystal data and details of the structure determination and refinement are listed in ESI Table S3, hydrogen bonds in ESI Table S5, atomic coordinates and equivalent isotropic displacement parameters are listed in ESI Table S6, hydrogen coordinates and equivalent isotropic displacement parameters are listed in ESI Table S7, anisotropic displacement parameters are listed in ESI Table S8, bond lengths and angles are listed in ESI Tables S9 and S10, respectively, and torsion angles are listed in ESI Table S11.

The FT-IR and far-IR spectra of samples were recorded in attenuated total reflection (ATR) mode with a BioRad-Digilab FTS-30-FIR and a Bruker Alpha IR spectrometer for the 4000–400 and 400–100 cm^–1^ ranges, respectively, at room temperature.

The Raman measurements of compound **1** were performed at 123 and 298 K on a Horiba Jobin-Yvon LabRAMtype microspectrometer supplied with an external (532 nm) Nd-laser source operated at ∼40 mW) and an Olympus BX-40 optical microscope (Linkam THMS600, with a temperature-controllable microscope stage). The laser beam (20× objective) was focused and a D1 intensity filter was applied to decrease the laser power in order to prevent thermal degradation of the sample. A confocal hole of 1000 μm and a 1800 groove mm^–1^ grating monochromator were used for light dispersion. The spectral range (100–4000 cm^–1^) was measured with a 3 cm^–1^ resolution and 120 s exposure time.

The UV–VIS diffuse reflectance spectra of the studied samples were measured at room temperature with a Jasco V-670 UV–vis spectrophotometer equipped with a NV-470 integrating sphere. BaSO_4_ was used as standard.

The low-temperature DSC measurements were performed with a Setaram DSC92 calorimeter, equipped with a liquid nitrogen cryostat; the temperature range was −140 to +45 °C, and the heating rate was 5 °C/min. The high-temperature DSC measurements (between 25 and 800 °C) were performed by means of a modified PerkinElmer DSC-3 calorimeter. Sample masses were varied between 3 and 6 mg, and the heating rate was 5 °C min^–1^ under a continuous argon flow (20 cm^3^ min^–1^). The aluminum pans were unsealed.

Thermogravimetry mass spectrometry (TG/MS) measurements were performed with a modified PerkinElmer TGS-2 instrument coupled to a HiQuad quadrupole mass spectrometer. In order to avoid explosion, approximately 1 mg of sample was heated slowly in a platinum sample pan in each measurement. Decomposition was followed in argon or air as carrier gas (flow rate = 140 cm^3^ min^–1^), from ambient temperature to 500 °C at a 5 °C min^–1^ heating rate. Selected ions between *m*/*z* = 2–88 were monitored in selected ion monitoring (SIM) mode. From the ion intensity curve *m*/*z* = 17, the ion intensity of *m*/*z* = 18 (water) was subtracted in proportion to the MS fragmentation of water, so the ion curve *m*/*z* = 17 shows the formation of ammonia. The ion intensity curve *m*/*z* = 28 was modified similarly, taking into account the fragmentation of CO_2_ (*m*/*z* = 44).

The X-ray photoelectron spectroscopy (XPS) analyses of the sample performed in inert atmosphere at 800 °C were measured on a Kratos XSAM 800 instrument (Mg K_α1,2_ excitation, 1253.6 eV, fixed analyzer transmission mode, room temperature). The analysis chamber pressure was selected to be <10^–7^ Pa. The spectra were recorded at 40 eV pass energy and charge-corrected with the use of C 1s (C–H, C–C, and adventitious carbon) and with BE set to 284.5 eV. The measured spectra were processed with Vision 2 software. The Shirley background and Lorentzian–Gaussian shape of photoemission lines (30% of Lorentzian contribution) were used for the evaluation of the photoemission lines. Surface compositions were calculated with XPS Multiquant software.^67^ The surface compositions were corrected to adventitious carbon content.

^57^Fe Mössbauer spectroscopy measurements were performed at room temperature and at *T* = 80 K with a conventional Mössbauer spectrometer (WissEl, Starnberg, Germany) operating in constant acceleration mode with a ^57^Co source in a Rh matrix. For low-temperature measurements, the samples were kept in a cryostat (SVT-400-MOSS, Janis, Woburn, MA, USA) filled with liquid nitrogen. The random orientation of the powder samples was provided by mixing with polyethylene powder. The Mössbauer spectra were evaluated by standard computer-based statistical analysis methods that included fitting the experimental data by a sum of Lorentzians or relaxational line shapes using a least-squares minimization procedure with the help of the MossWinn 4.0 program.^45^ The isomer shifts are given relative to α-Fe at room temperature.

GC–MS measurements were performed on a Shimadzu QP2010 SE instrument with the use of a 30 m ZB-WAX PLUS capillary column (injector temperature was 300 °C, the split ratio was 300, and He was used as carrier gas with a flow rate of 0.87 mL min^–1^). The column was heated from 70 to 340 °C with a heating rate of 20 °C min^–1^ and kept for 15.5 min at this temperature. The interface temperature was 325 °C, the ionization energy was selected to be 70 keV, the MS ion source temperature was adjusted to 260 °C, the detector was scanned in the 10–800 *m*/*z* range, and the MS spectra were recorded at 2.3 min after injection.

TEM data were acquired with a 200 keV Talos Thermo Scientific transmission electron microscope. Grains were crushed under ethanol and deposited onto copper grids covered by lacey carbon. We obtained BFTEM and HAADF-STEM images as well as SAED patterns. The elemental composition of the grains was measured with a “Super-X” detector system having four silicon drift detectors built into the microscope column. Radial distributions of intensities were generated from SAED patterns following the method described by Lábár^54^ and using ProcessDiffraction software.^55^

Scanning electron microscopy (SEM) measurements were performed with a Zeiss EVO40 microscope operating at 20 kV.

#### Organic Oxidation Reactions

The oxidation reactions of organic substrates (1.3 mmol) with compound **1** (2.6 mmol) were performed in a 250 mL round-bottom flask supplied with a reflux condenser in 30 mL of benzene as solvent. The reaction mixture was stirred for 2 h at room temperature or, in several cases, refluxed for 2 or 4 h. The organic compounds formed were identified with GC–MS, whereas the isolated yield of compounds was determined with the formation of 2,4-dinitrophenylhydrazone precipitate. The 2,4-dinitrophenylhydrazine reagent was made with the dissolution of 1.2 g of 2,4-dinitrophenylhydrazine) in 50 mL of 30% aq perchloric acid). The reagent was diluted with water (2 times) and added to the filtered benzene solution diluted with ethanol, and after separation of the benzene, the ethanolic suspension was filtered and dried.

#### Catalytic CO_2_ Hydrogenation Experiments

The catalytic reaction (CO_2_ hydrogenation) was studied in the microreactor Microactivity Effi. Catalysts were studied in the temperature range of 175–550 °C (steps of 25 °C, 4 h for each temperature). It means 16 temperatures and 64 h of analysis. Products were analyzed in hourly intervals by CG/MS (connected to the reactor, Agilent 7890B and MS-Agilent 5977B MSD). The pressure was 20 bars, and gas flows were He/H_2_/CO_2_ = 12/6/2 N mL/min.

#### Computational Details

The utilized computational DFT methodology is based on ref (46). The structure of the [hexakis(urea-*O*)iron(III)] high-spin complex cation was fully optimized at the BP86^68,69^/TZVP level with D3 dispersion correction^70^/Becke–Johnson damping.^71^ Environmental effects were taken into account by the conductor-like screening model (COSMO)^72^ with a dielectric constant ε = 32.63 (methanol). The three parameters required for the Mössbauer data analysis, the isomer shift, η, and *V_zz_*, were calculated according to ref (46). Specifically, the isomer shift was obtained by the DFT-calculated electron density at the Fe nucleus ρ(0), utilizing the expression δ = αρ(0) + β, where the α,β calibration constants were determined by a linear fit of experimental δ vs DFT-calculated ρ(0) values. The η and *V_zz_* parameters were calculated by the diagonalization of the traceless electric field gradient matrix. We used the B3LYP^73−75^ exchange-correlation functional for the isomer shift and TPSS^76,77^ for the quadrupole splitting in conjunction with TZVP for all atoms, except Fe, for which we used the CP(PPP) core-polarized basis set.^78,79^ Two-electron integrals were approximated by the resolution of identity (RI-J)^80^ and chain of spheres (COSX) methods.^81^ For Fe, we increased the integral accuracy parameter to 7.0 in order to obtain accurate core properties. All calculations were carried out with the ORCA3.0 quantum chemistry software.^82^

## Conclusions

An optimized preparation route and a new way to grow single crystals of [hexakis(urea-*O*)iron(III)] permanganate (compound **1**) proved to be feasible by freezing out ice from its saturated aqueous solution at ∼0 °C .

The structural features of compound **1**, including the extended hydrogen bond network, have also been determined and characterized by spectroscopic (IR, Raman Mössbauer) and single-crystal X-ray diffraction methods.

Hexakis(urea-*O*)iron(III) permanganate was found to be a selective and mild oxidant in the transformation of (un)substituted benzylic alcohols into benzaldehydes and benzonitriles.

The thermal decomposition of compound **1** revealed a thermally induced solid phase quasi-intramolecular redox reaction between the urea ligands and permanganate ions at 120 °C, below the urea ligand loss temperature of hexaureairon(III) cations. The decomposition mechanism of the residual urea ligands (water and ammonia elimination with the formation of isocyanuric acid and biuret, respectively) has also been clarified.

The final solid thermal decomposition products at 800 °C were manganese-containing wuestite (Fe,Mn)O, as well as two types of bixbyite (Fe,Mn)_2_O_3_ with overall 1:3 Fe to Mn stoichiometries, in inert and air atmospheres, respectively. The evolution of Fe-containing intermediate phases was followed by Mössbauer and XPS spectroscopy and TEM measurements. An amorphous iron(III)-containing intermediate and a mixture of bixbyite, hausmannite, and jacobsite (FeMn_2_O_4_) formed in ambient air. The primarily formed bixbyite with a double-size supercell and inhomogeneous Fe and Mn distribution transformed into common bixbyite. Increasing the heating time and the temperature accelerated the transformation of irregular bixbyite structure into the regular crystalline form.

The iron(III) content of the amorphous material was reduced at around 550 °C and crystallized into wuestite (Fe,Mn)O at 800 °C in an inert atmosphere. The thermal decomposition intermediates of compound **1** formed in either inert or air atmosphere at 350 °C transformed into the same phases depending on the presence or absence of oxygen during further heating. In the inert atmosphere, both intermediates gave wuestite, whereas in air, bixbyite was produced.

Two kinds of bixbyite, a disordered variation with a double-size supercell and the common regular bixbyite, were also obtained. The disordered bixbyite transforms into the regular form upon heating. The distribution of iron(III) between the two kinds of bixbyite as well as the ratio of T-4 and OC-6 occupancy in the intermediates (jacobsite) and products (bixbyites) depending on the temperature and time of the heat treatment were also determined.

The (Fe,Mn)O_*x*_ intermediates formed under various conditions were tested as catalysts in the CO_2_ hydrogenation reaction. Most of them catalyze the reduction by hydrogen in 64 h with <57.6% CO_2_ conversions and <39.3% hydrocarbon yields.

## Supporting Information Available

Powder XRD data and patterns, single-crystal structures, packing information, and numerical structural data of compound **1**; DSC results of compound **1**; Mössbauer spectra and parameters of aerial decomposition products of compound **1**; correlation analysis results and low- and room-temperature IR and Raman spectra and vibrational spectroscopic evaluation of compound **1**; far-IR and UV spectra of compound **1**; DFT-optimized structure and experimental Mössbauer spectra and parameters of compound **1**; thermal analysis (TG-MS and DSC) data and curves of compound **1** in various (inert and air) atmospheres; powder XRD, XPS, and Mössbauer spectra and parameters; SEM pictures of intermediate phases (formed in N_2_ and air) during decomposition of compound **1**; catalytic properties of the thermal decomposition products of compound **1** (PDF)
