# Supplementary material for: Thermally Induced Solid-Phase Quasi-Intramolecular Redox Reactions of [Hexakis(urea-O)iron(III)] Permanganate: An Easy Reaction Route to Prepare Potential (Fe,Mn)Ox Catalysts for CO2 Hydrogenation
Source: Inorg Chem. 2022 Aug 31;61(36):14403–18. doi: 10.1021/acs.inorgchem.2c02265 (PMC9477215; doi:10.1021/acs.inorgchem.2c02265)
Supplement: Supplementary file 1 — ic2c02265_si_001.pdf [file ic2c02265_si_001.pdf]

Supporting Information

Thermally-induced solid-phase quasi-intramolecular redox reactions of [hexakis(urea-O)iron(III)] permanganate – an easy reaction route to prepare potential (Fe,Mn)O_x catalysts for CO_2 hydrogenation

*Kende Attila Béres[1,2], Zoltán Homonnay[2], Libor Kvitek[3], Zsolt Dürvanger[4], Martina Kubikova[3], Veronika Harmat[4,5], Fanni Szilágyi[1,6], Zsuzsanna Czégény[1], Péter Németh[7], Laura Bereczki[1], Vladimir M. Petruševski[8], Mátyás Pápai[9], Attila Farkas[10] and László Kótai[1,11] **

[1]Institute of Materials and Environmental Chemistry, Research Centre for Natural Sciences, Magyar Tudósok krt. 2., H-1117 Budapest, Hungary;

[2]Institute of Chemistry, ELTE Eötvös Loránd University, Pázmány Péter s. 1/A, H-1117 Budapest, Hungary

[3]Palacky University Olomouc, Faculty of Science, Department of Physical Chemistry, 17. Listopadu 12, Olomouc 77146, Czech Republic

[4]Structural Chemistry and Biology Laboratory, Institute of Chemistry, ELTE Eötvös Loránd University, Pázmány Péter s. 1/A, H-1117 Budapest, Hungary

[5]ELKH-ELTE Protein Modelling Research Group, Pázmány Péter s. 1/A, H-1117 Budapest, Hungary

[6]Bay Zoltan Ltd. for Applied Research, Production Division (BAY-PROD), 1 Kondorfa, Budapest 1116, Hungary

[7]Institute for Geological and Geochemical Research, Research Centre for Astronomy and Earth Sciences, ELKH, Budaörsi street 45, 1112 Budapest, Hungary

[8]Faculty of Natural Sciences and Mathematics, Ss. Cyril and Methodius University,

Skopje, MK-1000, North Macedonia

[9]Wigner Research Centre for Physics, H-1525 Budapest, P.O. Box 49, Hungary

[10]Department of Organic Chemistry, Budapest University of Technology and Economics, Műegyetem rakpart 3, 1111 Budapest, Hungary

[11]Deuton-X Ltd., Selmeci u. 89, H-2030, Érd, Hungary

* Corresponding author: kotai.laszlo@ttk.hu

ESI Table S1. The Powder X-ray diffraction data of compound **1** refined to trigonal cell.

18.1241 18.1241 13.5493
90 90 120
sg=R3 unindexed=0 vol=3854.406 gof=40.97 zero=0

h	*k*	*l*	dc	do	do-dc	2Thc	2Tho	2Tho-2Thc
1	0	1	10.2565			8.6144		
1	1	0	9.0620	9.0480	-0.0140	9.7524	9.7675	0.0151
0	2	1	6.7910	6.7809	-0.0102	13.0260	13.0456	0.0196
0	1	2	6.2200	6.2234	0.0034	14.2278	14.2200	-0.0078
1	2	-1	5.4344	5.4287	-0.0057	16.2976	16.3148	0.0172
0	3	0	5.2320	5.2336	0.0016	16.9327	16.9274	-0.0053
2	0	2	5.1282	5.1284	0.0001	17.2779	17.2775	-0.0004
2	2	0	4.5310	4.5284	-0.0027	19.5762	19.5879	0.0117
0	0	3	4.5164			19.6401		
1	2	2	4.4631	4.4647	0.0016	19.8770	19.8698	-0.0072
1	-4	-1	4.1446	4.1437	-0.0009	21.4221	21.4266	0.0046
1	-2	-3	4.0422	4.0411	-0.0012	219713	21.9777	0.0064
4	0	1	3.7691			23.5854		
1	3	-2	3.6623	3.6638	0.0014	24.2834	24.2737	-0.0097
2	3	-1	3.4801	3.4802	0.0002	25.5760	25.5748	-0.0012
4	1	0	3.4251	3.4248	-0.0003	25.9935	25.9962	0.0027
3	0	3	3.4188			26.0424		
0	4	2	3.3955			26.2242		
1	0	4	3.3111	3.3114	0.0003	26.9053	26.9029	-0.0023
2	2	3	3.1987	3.1979	-0.0008	27.8690	27.8762	0.0073
2	-5	-2	3.1796			28.0398		
0	2	4	3.1100			28.6810		
0	5	1	3.0582			29.1777		
3	3	0	3.0207	3.0217	0.0010	29.5481	29.5377	-0.0104
2	1	4	2.9416	2.9417	0.0001	30.3614	30.3607	-0.0007
2	-6	-1	2.8976	2.9000	0.0024	30.8334	30.8078	-0.0257
5	0	2	2.8483	2.8472	-0.0011	31.3815	31.3939	0.0125
1	5	-1	2.7600	2.7601	0.0001	32.4126	32.4115	-0.0012
1	-5	-3	2.7291			32.7895		
4	2	2	2.7172			32.9371		
1	-4	-4	2.6734	2.6730	-0.0004	33.4931	33.4983	0.0052
0	1	5	2.6704			33.5319		
0	6	0	2.6160			34.2500		
1	5	2	2.6027	2.6019	-0.0008	34.4300	34.4409	0.0108
4	0	4	2.5641	2.5659	0.0018	34.9651	34.9397	-0.0253
2	0	5	2.5615			35.0025		

ESI Table S2. The Powder X-ray diffraction data of compound **1** refined to monoclinic cell.

13.8479 18.1275 11.4037
90 64.122 90
sg=Cc unindexed=0 vol=2575.596 gof=43.16 zero=0.0183

h	*k*	*l*	dc	do	do-dc	2Thc	2Tho	2Tho-2Thc
1	1	0	10.2679			8.6048		
1	1	1	9.0700			9.7438		
0	2	0	9.0638	9.0480	-0.0157	9.7505	9.7675	0.0170
0	2	1	6.7928	6.7809	-0.0120	13.0225	13.0456	0.0231
2	0	0	6.2297			14.2056		
1	-1	-1	6.2242	6.2234	-0.0008	14.2181	14.2200	0.0019
2	2	1	5.4407			16.2787		
1	1	2	5.4373			16.2890		
1	3	0	5.4368	5.4287	-0.0081	16.2903	16.3148	0.0245
2	0	2	5.2378			16.9138		
1	3	1	5.2342	5.2336	-0.0005	16.9256	16.9274	0.0018
2	2	0	5.1339			17.2585		
0	0	2	5.1301	5.1284	-0.0017	17.2715	17.2775	0.0059
2	2	2	4.5350			19.5589		
0	4	0	4.5319	4.5284	-0.0035	19.5725	19.5879	0.0154
3	1	1	4.4706			19.8434		
1	-3	-1	4.4651			19.8682		
0	2	2	4.4646	4.4647	0.0002	19.8706	19.8698	-0.0008
3	1	2	4.1506			21.3906		
1	3	2	4.1462			21.4138		
0	4	1	4.1455	4.1437	-0.0018	21.4174	21.4266	0.0093
3	1	0	4.0482			21.9383		
2	-2	-1	4.0457			21.9522		
1	-1	-2	4.0443	4.0411	-0.0032	21.9600	21.9777	0.0177
2	4	1	3.7716			23.5698		
3	3	1	3.6667			24.2541		
2	4	0	3.6648			24.2671		
1	1	3	3.6639	3.6638	-0.0001	24.2729	24.2737	0.0008
3	3	2	3.4838			25.5480		
2	2	3	3.4823			25.5592		
1	5	0	3.4811	3.4802	-0.0009	25.5683	25.5748	0.0065
3	1	3	3.4288			25.9651		
2	4	2	3.4271			25.9781		
1	5	1	3.4261	3.4249	-0.0012	25.9859	25.9955	0.0096
3	3	0	3.4226			26.0129		
1	-3	-2	3.4202			26.0313		
4	0	2	3.4013			26.1789		
0	4	2	3.3964			26.2171		
3	-1	-1	3.3149			26.8739		
2	0	-2	3.3135	3.3114	-0.0022	26.8850	26.9029	0.0179
4	2	1	3.2037			27.8246		
2	-4	-1	3.2007			27.8517		
0	2	3	3.1998	3.1979	-0.0019	27.8592	27.8762	0.0170
4	2	2	3.1845			27.9966		
1	3	3	3.1808			28.0293		
1	-5	-1	3.1806			28.0308		
4	0	0	3.1148			28.6356		
2	-2	-2	3.1121			28.6613		
1	5	2	3.0591			29.1689		
3	3	3	3.0233			29.5216		

0	6	0	3.0213	3.0217	0.0005	29.5424	29.5377	-0.0048
4	2	0	2.9457			30.3177		
3	-3	-1	2.9444			30.3321		
1	-1	-3	2.9429	2.9417	-0.0013	30.3474	30.3607	0.0133
4	2	3	2.9014			30.7928		
2	4	3	2.8991			30.8175		
0	6	1	2.8982	2.8964	-0.0018	30.8271	30.8464	0.0194
3	5	1	2.8505			31.3560		
2	0	4	2.8499	2.8472	-0.0027	31.3635	31.3939	0.0304
3	1	4	2.7622			32.3858		
3	5	2	2.7620			32.3877		
2	6	1	2.7612	2.7601	-0.0011	32.3981	32.4115	0.0134
5	1	2	2.7338			32.7315		
4	4	1	2.7323			32.7495		
3	5	0	2.7312			32.7633		
1	1	4	2.7302			32.7762		
1	-5	-2	2.7300			32.7782		
0	4	3	2.7299			32.7793		
4	4	2	2.7203			32.8980		
2	2	4	2.7186			32.9193		
2	6	0	2.7184			32.9219		
5	1	1	2.6779			33.4346		
2	-4	-2	2.6748			33.4742		
1	-3	-3	2.6744			33.4792		
3	-1	-2	2.6727	2.6730	0.0002	33.5012	33.4983	-0.0029
4	0	4	2.6189			34.2109		
2	6	2	2.6171			34.2353		
5	1	3	2.6068			34.3743		
1	5	3	2.6035			34.4191		
0	6	2	2.6033	2.6019	-0.0014	34.4217	34.4409	0.0191
4	4	0	2.5670			34.9249		
0	0	4	2.5650	2.5659	0.0009	34.9519	34.9397	-0.0122
4	-2	-1	2.5645			34.9599		
4	4	3	2.5374			35.3445		
3	3	4	2.5366			35.3562		
1	7	0	2.5355			35.3732		
4	2	4	2.5160			35.6564		
3	5	3	2.5150			35.6701		
5	3	2	2.5146			35.6765		
1	7	1	2.5140			35.6857		
2	-6	-1	2.5120			35.7148		
1	3	4	2.5118			35.7180		
5	3	1	2.4709			36.3294		
3	-5	-1	2.4690			36.3578		
5	1	0	2.4686			36.3635		
0	2	4	2.4681			36.3716		
3	-3	-2	2.4668	2.4667	-0.0002	36.3914	36.3939	0.0026
2	-2	-3	2.4663			36.3995		

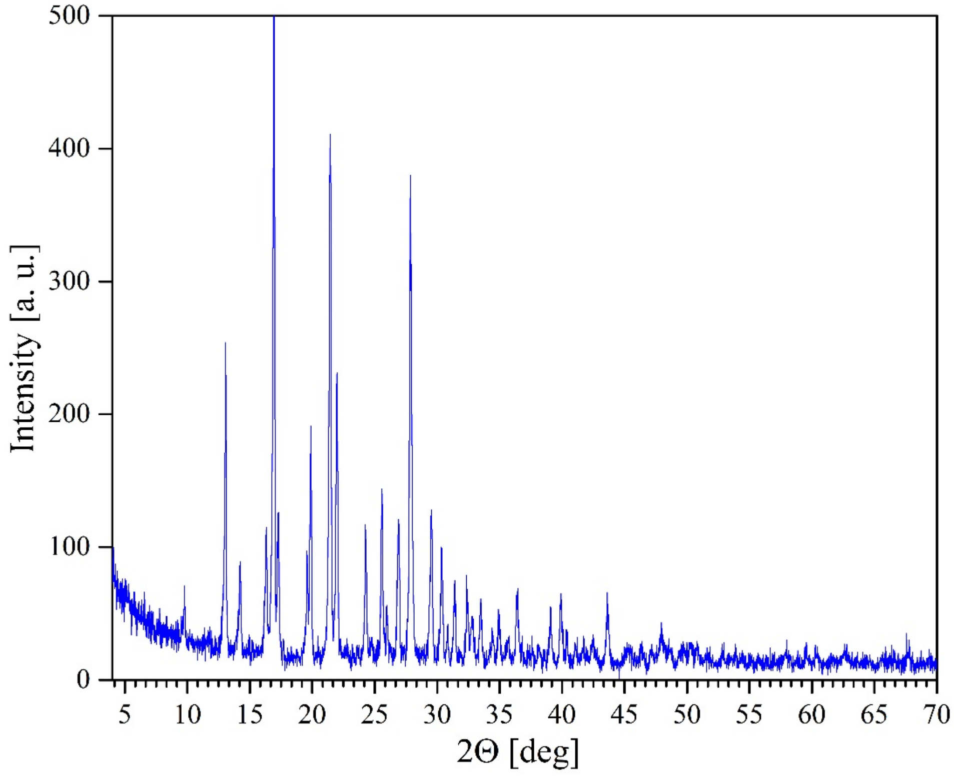

ESI Figure S1. The powder XRD of the compound **1.**

ESI Table S3. Single crystal XRD experiments. Crystal data and structure refinement of compound **1**.

Empirical formula	$C_6H_{24}FeMn_3N_{12}O_{18}$
Formula weight	773.04
Temperature	100.00(10)
Radiation and wavelength	Cu-Kα, λ =1.54184Å
Crystal system	monoclinic
Space group	$P2_1/c$
Unit cell dimensions	*a* =13.7008(7)Å
	b =18.0084(5)Å
	c =11.4125(4)Å
	α = 90°
	β = 112.988(5)°
	γ = 90°
Volume	2592.2(2)$Å^3$
Z	4
Density (calculated)	1.981 Mg/m^3
Absorption coefficient, *μ*	16.959 mm^{-1}
F(000)	1556.0
Crystal colour	dark purple
Crystal description	needle
Crystal size	0.273 × 0.071 × 0.058 mm
Absorption correction	numerical
Max. and min. transmission	1.00000 0.05829
θ range for data collection	$7.008 \leq 2\theta \leq 150.808°$
Index ranges	$-17 \leq h \leq 16$, $-22 \leq k \leq 17$, $-14 \leq l \leq 12$
Reflections collected	23228
Completeness to 2 *θ*	0.952
Independent reflections	5128 [*R*(int) =0.0711]
Reflections $I>2\sigma(I)$	4231
Refinement method	full-matrix least-squares on F^2
Data / restraints / parameters	5128/93/380
Goodness-of-fit on F^2	1.089
Final *R* indices [$I>2\sigma(I)$]	R_1 = 0.0728, wR_2 = 0.1862
R indices (all data)	R_1 = 0.0866, wR_2 = 0.1946
Max. and mean shift/esd	0.000; 0.000
Largest diff. peak and hole	1.63/-0.92 e.$Å^{-3}$

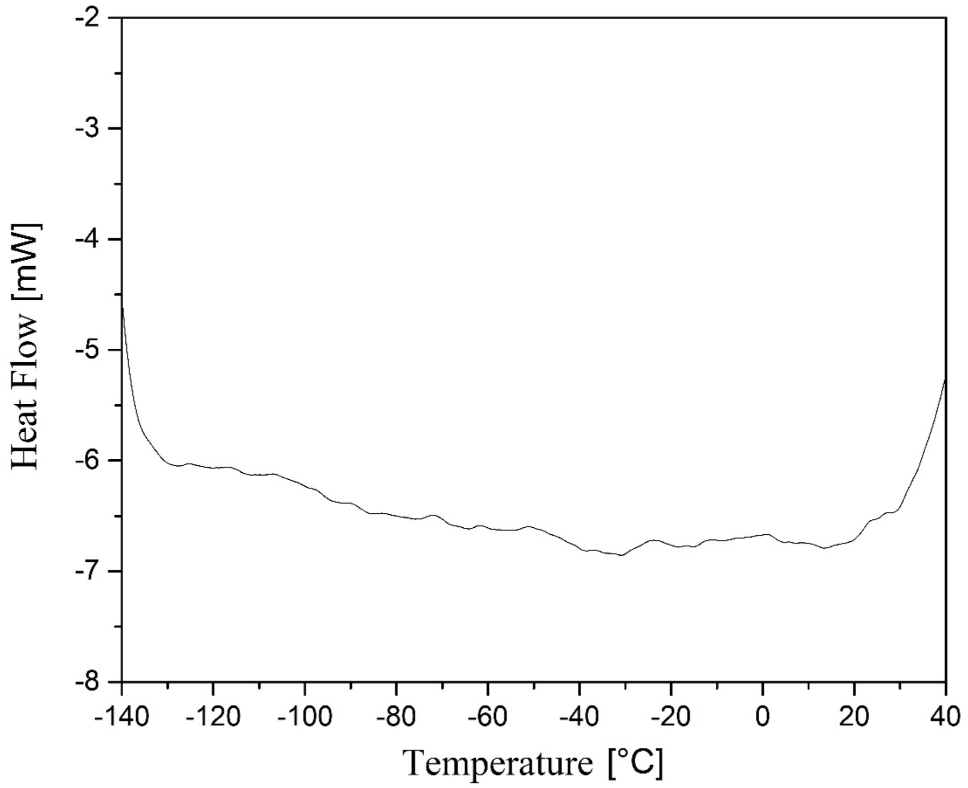

ESI Figure S2. The low-temperature DSC measurement of compound **1**.

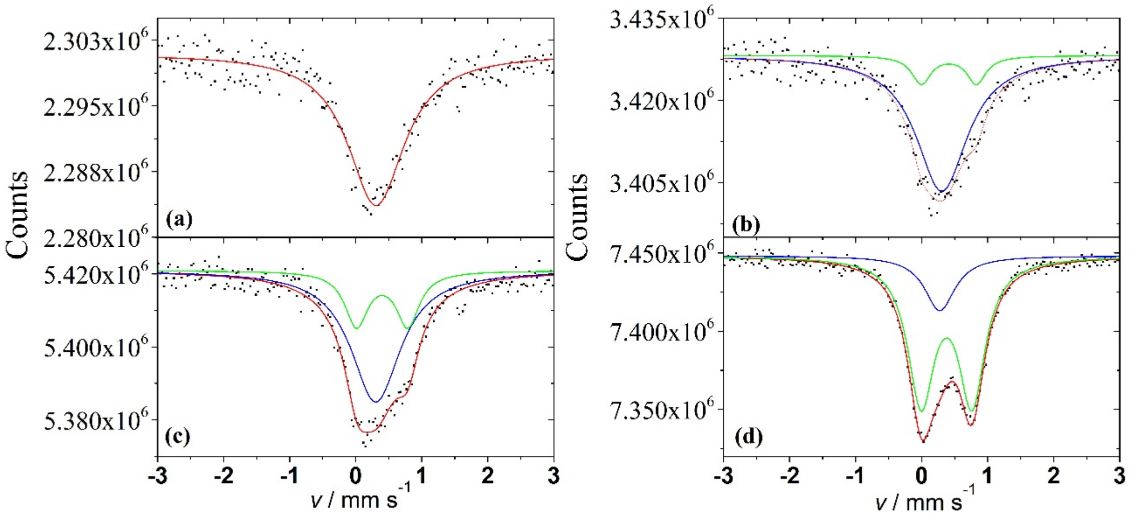

ESI Figure S3. Mössbauer spectra showing the decomposition of compound **1** on standing in air at room temperature for 1, 2, 3 and 5 days, (a, b, c and d, respectively).

ESI Table S4. The Mössbauer parameters of compound **1** and its decomposition products obtained while recording the Mössbauer spectrum for up to 5 days at room temperature.

	Day 1	Day2		Day3		Day 5	
Component	Singlet	Singlet	Doublet	Singlet	Doublet	Singlet	Doublet
Relative area (%)	100	86.71	13.29	72.40	27.60	18.13	81.87
$\boldsymbol{\delta}$ ($mm{\cdot}s^{-1}$)	0.315 (±0.012)	0.306 (±0.011)	0.412 (±0.023)	0.304 (±0.011)	0.400 (±0.012)	0.275 (±0.010)	0.377 (±0.003)
$\boldsymbol{\Delta}$ ($mm{\cdot}s^{-1}$)	---	---	0.834 (±0.054)	---	0.776 (±0.032)	---	0.770 (±0.007)
$\boldsymbol{\Gamma}$ ($mm{\cdot}s^{-1}$)	1.036 (±0.063)	0.948 (±0.066)	0.352 (±0.130)	0.917 (±0.067)	0.424 (±0.078)	0.563 (±0.083)	0.490 (±0.012)

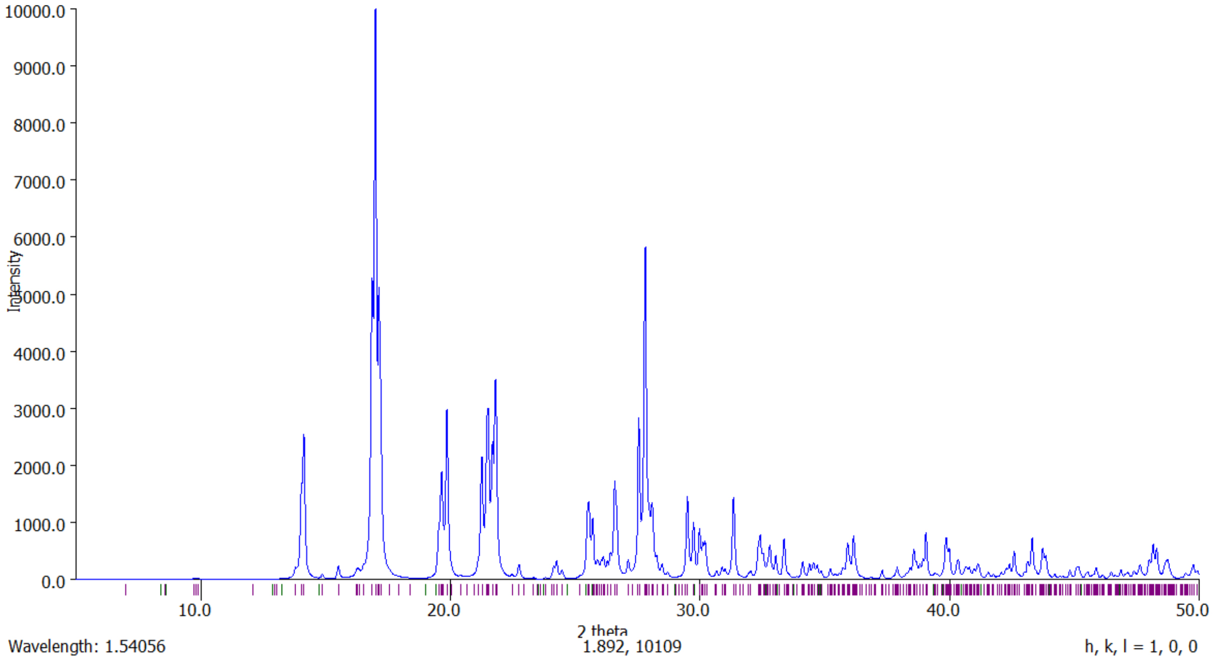

ESI Figure S4. Single crystal XRD experiment on compound **1**. Calculated powder pattern.

ESI Table S5. Hydrogen bonds in the crystal structure of compound **1**.

D-H···A	d(D-H)/Å	d(H-A)/Å	d(D-A)/Å	D-H-A/°	sym op
**Intramolecular H-bond**					
N12-H12A..O2	0.88	2.19	2.937(7)	142.3	
N4-H4A..O6	0.88	2.20	2.920(7)	139.0	
N8-H8A..O10	0.88	2.18	2.915(7)	140.6	
N24-H24A..O14	0.88	2.20	2.952(7)	142.5	
N16-H16A..O18	0.88	2.21	2.940(7)	140.0	
N20-H20A..O22	0.88	2.22	2.966(7)	142.6	
**Intermolecular, urea 1**					
N4-H4A..O38A	0.88	2.49	3.146(7)	131.7	*+x,1/2-y,1/2+z*
N4-H4B..O29	0.88	2.61	3.322(7)	138.9	*+x,+y,1+z*
N4-H4B..O34	0.88	2.63	3.222(7)	125.5	
N4-H4B..O40	0.88	2.37	3.015(7)	130.0	*+x,1/2-y,1/2+z*
N5-H5A..O28	0.88	2.12	2.946(7)	154.9	*+x,3/2-y,1/2+z*
N5-H5A..O27B	0.88	2.55	3.27(4)	139.2	*+x,3/2-y,1/2+z*
N5-H5B..O29	0.88	2.29	3.077(7)	148.8	*+x,+y,1+z*
N5-H5B..O30	0.88	2.42	2.951(7)	118.7	*+x,+y,1+z*
**Intermolecular, urea 2**					
N8-H8A..O27A	0.88	2.64	3.292(8)	132.1	*-x,1-y,-z*
N8-H8B..O39	0.88	2.27	2.989(7)	138.2	
N8-H8B..O28	0.88	2.62	3.283(8)	132.5	*-x,-1/2+y,1/2-z*
N8-H8B..O30	0.88	2.48	3.058(7)	123.5	*-x,1-y,-z*
N9-H9A..O39	0.88	2.66	3.319(8)	132.9	*+x,1/2-y,1/2+z*
N9-H9A..O38A	0.88	2.21	3.039(8)	157.5	*+x,1/2-y,1/2+z*
N9-H9B..O27A	0.88	2.50	3.337(8)	158.7	*-x,-1/2+y,1/2-z*
N9-H9B..O38B	0.88	2.17	2.96(4)	149.7	
**Intermolecular, urea 3**					
N12-H12B..O39	0.88	2.15	2.980(8)	156.7	*-x,1/2+y,1/2-z*
N12-H12B..O30	0.88	2.43	2.876(7)	111.9	*+x,3/2-y,1/2+z*
N13-H13A..O29	0.88	2.50	3.102(7)	126.5	*-x,1-y,-z*
N13-H13A..O27A	0.88	2.26	3.098(8)	159.8	*-x,1-y,-z*
N13-H13B..O39	0.88	2.57	3.298(8)	140.5	*-x,1/2+y,1/2-z*
N13-H13B..O40	0.88	2.38	3.021(8)	129.5	*-x,1/2+y,1/2-z*
N13-H13B..O27A	0.88	2.65	3.364(8)	138.5	
**Intermolecular, urea 4**					
N16-H16A..O32A	0.88	2.61	3.252(8)	131.0	*+x,3/2-y,-1/2+z*
N16-H16B..O33	0.88	2.33	3.056(7)	139.8	
N16-H16B..O35	0.88	2.51	3.100(7)	124.7	*+x,3/2-y,-1/2+z*
N17-H17A..O34	0.88	2.21	3.037(8)	155.7	*1-x,1-y,2-z*
N17-H17A..O32B	0.88	2.55	3.25(4)	137.7	*1-x,1-y,2-z*
N17-H17B..O33	0.88	2.65	3.306(7)	132.3	
N17-H17B..O37	0.88	2.59	3.275(8)	135.3	*1-x,1/2+y,3/2-z*
N17-H17B..O32B	0.88	2.29	3.04(4)	143.4	
**Intermolecular, urea 5**					
N20-H20B..O29	0.88	2.39	3.079(7)	135.9	
N20-H20B..O34	0.88	2.44	3.143(8)	137.1	*+x,+y,-1+z*
N20-H20B..O40	0.88	2.50	2.918(7)	110.1	*+x,1/2-y,-1/2+z*
N21-H21A..O32A	0.88	2.22	3.063(8)	161.3	*+x,3/2-y,-1/2+z*
N21-H21B..O32A	0.88	2.49	3.339(8)	163.1	*+x,+y,-1+z*
N21-H21B..O27B	0.88	2.21	3.04(4)	158.1	
**Intermolecular, urea 6**					
N24-H24B..O33	0.88	2.26	3.062(7)	151.8	*1-x,-1/2+y,3/2-z*
N24-H24B..O35	0.88	2.42	2.865(7)	111.7	*1-x,1-y,2-z*
N25-H25A..O37	0.88	2.17	2.926(8)	143.2	*+x,1/2-y,-1/2+z*
N25-H25A..O38B	0.88	2.37	3.17(4)	150.4	*+x,1/2-y,-1/2+z*
N25-H25B..O33	0.88	2.48	3.229(7)	143.7	*1-x,-1/2+y,3/2-z*
N25-H25B..O35	0.88	2.38	3.009(8)	129.1	*1-x,-1/2+y,3/2-z*

ESI Table S6. Single crystal XRD experiment on compound **1**. Atomic coordinates (x 10^4) and equivalent isotropic displacement parameters (Å^2 x 10^3). U(eq) is defined as one third of the trace of the orthogonalized U_{ij} tensor.

Atom	***x***	***y***	***z***	***U(eq)***
Fe1	2545.7(7)	5014.3(5)	4932.7(8)	21.0(3)
O6	2076(4)	4185(2)	5747(4)	25.2(9)
O14	3907(4)	5041(2)	6488(4)	27.3(10)
O18	3030(4)	5873(2)	4200(4)	27.1(10)
O10	1206(4)	4939(2)	3371(4)	28.7(10)
O2	1876(4)	5749(2)	5703(4)	23.9(9)
O22	3183(4)	4292(2)	4100(4)	27.5(10)
O33	4675(4)	7080(2)	9409(4)	29.8(10)
O29	1363(4)	6170(2)	-357(4)	28.5(10)
O37	3278(4)	1794(3)	5938(4)	37.8(12)
O35	4909(4)	7096(3)	11807(4)	35.4(11)
O34	3864(4)	6001(2)	10320(5)	36.3(11)
O39	1442(4)	1763(2)	3951(4)	34.4(11)
O28	589(4)	7213(3)	669(5)	40.0(12)
O30	415(4)	7328(3)	-1712(4)	37.7(12)
N25	3555(5)	3151(3)	3612(6)	35.8(14)
N9	1744(5)	3035(3)	6221(6)	37.0(14)
O40	2344(4)	509(3)	5060(5)	38.8(12)
N4	2242(5)	5289(3)	7691(5)	31.9(13)
N16	4194(5)	6279(3)	6884(6)	34.7(13)
N8	1160(5)	3399(3)	4133(6)	35.5(14)
N12	454(5)	6082(3)	3067(6)	43.1(17)
N5	1718(5)	6502(3)	7194(5)	33.2(13)
N21	2860(5)	6738(3)	2709(5)	35.8(14)
N20	2828(5)	5502(3)	2213(5)	38.6(15)
N13	-12(5)	5238(3)	1460(6)	38.0(14)
N24	4177(5)	3524(3)	5684(6)	41.8(16)
N17	5028(5)	5418(3)	8408(6)	39.7(15)
C19	2904(5)	6030(4)	3059(6)	27.9(14)
C3	1938(5)	5837(3)	6844(6)	26.1(13)
C23	3635(5)	3669(3)	4463(6)	29.9(14)
C11	564(5)	5416(3)	2654(6)	28.5(14)
C15	4359(5)	5578(4)	7243(6)	30.1(14)
C7	1661(5)	3553(3)	5360(6)	29.8(14)
Mn2A	4143.7(9)	6862.6(5)	10393.3(9)	22.2(3)
Mn1A	443.5(9)	6779.1(6)	-613.9(10)	24.2(3)
Mn3A	2549.5(9)	1341.6(6)	4697.3(11)	25.6(3)
O32A	3054(4)	7331(3)	10037(5)	33.4(11)
O27A	-692(4)	6351(3)	-1104(5)	35.2(11)
O38A	3174(4)	1284(3)	3745(5)	37.9(12)
Mn1B	1077(13)	7014(7)	-314(11)	24.2(3)
Mn2B	4755(12)	6620(7)	10628(10)	22.2(3)
Mn3B	2246(11)	1357(6)	5113(14)	25.6(3)
O27B	2200(20)	7473(19)	110(30)	35.2(11)
O32B	5870(20)	6152(18)	11020(30)	33.4(11)
O38B	1600(30)	1390(20)	6110(30)	37.9(12)

ESI Table S7. Single crystal XRD experiment on compound **1**. Hydrogen coordinates (x 10^4) and isotropic displacement parameters (Å^2 x 10^3).

Atom	***x***	***y***	***z***	***U(iso)***
H25A	3190.46	3238.44	2798.39	43
H25B	3866.79	2718.7	3858.71	43
H9A	2079.24	3133.14	7036.42	44
H9B	1463.68	2593.03	5976.75	44
H4A	2407.44	4852.17	7473.26	38
H4B	2278.73	5362.21	8469.2	38
H16A	3761.55	6390.62	6103.02	42
H16B	4516.97	6634.2	7426.19	42
H8A	1106.95	3739.33	3558.25	43
H8B	881.39	2956.33	3894.12	43
H12A	831.38	6208.32	3860.96	52
H12B	3.06	6400.2	2548.49	52
H5A	1534.21	6870.52	6644.16	40
H5B	1756.44	6571.76	7973.55	40
H21A	2917.6	7093.06	3262.86	43
H21B	2773.08	6851.75	1924.99	43
H20A	2864.49	5031.38	2434.92	46
H20B	2741.1	5622.62	1431.18	46
H13A	51.46	4795.62	1170.98	46
H13B	-459.78	5562.42	952.81	46
H24A	4229.43	3861.96	6261.85	50
H24B	4484.68	3088.81	5917.04	50
H17A	5154.84	4952.01	8650.64	48
H17B	5347.03	5777.81	8942.24	48

ESI Table S8. Anisotropic displacement parameters (Å^2 x 10^3). The anisotropic displacement factor exponent takes the form: $-2\pi^2(h^2a^{*2}U_{11} + ... + 2hka^*b^*U_{12})$

Atom	U_{11}	U_{22}	U_{33}	U_{23}	U_{13}	U_{12}
Fe1	31.9(6)	15.2(4)	14.7(4)	-0.7(3)	7.8(4)	1.4(4)
O6	35(3)	17.7(19)	19(2)	-3.7(16)	7.2(18)	-5.2(17)
O14	34(3)	22(2)	20(2)	-4.6(17)	3.9(18)	-2.6(18)
O18	42(3)	21(2)	18(2)	0.3(16)	10.8(19)	-0.9(18)
O10	38(3)	21(2)	17(2)	0.9(16)	0.3(18)	5.6(18)
O2	34(2)	21.2(19)	17.0(19)	0.1(16)	10.3(18)	0.7(17)
O22	37(3)	20(2)	23(2)	0.4(17)	10.2(19)	9.4(18)
O33	41(3)	26(2)	28(2)	-1.6(18)	19(2)	-4.3(19)
O29	39(3)	23(2)	25(2)	6.0(17)	13.2(19)	7.0(19)
O37	44(3)	36(3)	29(2)	-1(2)	9(2)	1(2)
O35	45(3)	44(3)	18(2)	-3(2)	13(2)	-8(2)
O34	46(3)	27(2)	37(3)	1(2)	17(2)	-9(2)
O39	39(3)	25(2)	31(2)	0.8(19)	5(2)	2(2)
O28	45(3)	43(3)	32(3)	-13(2)	15(2)	3(2)
O30	54(3)	32(2)	29(3)	7(2)	18(2)	11(2)
N25	41(4)	29(3)	32(3)	-6(2)	8(3)	8(2)
N9	49(4)	24(3)	36(3)	1(2)	14(3)	-7(3)
O40	50(3)	20(2)	40(3)	7.6(19)	10(2)	2(2)
N4	50(4)	24(3)	23(3)	3(2)	16(3)	-2(2)
N16	45(4)	23(3)	30(3)	-4(2)	8(3)	-9(2)
N8	45(4)	22(3)	29(3)	-1(2)	3(3)	-7(2)
N12	56(4)	25(3)	30(3)	-4(2)	-3(3)	12(3)
N5	48(4)	27(3)	26(3)	-4(2)	16(3)	7(2)
N21	56(4)	27(3)	25(3)	4(2)	16(3)	-5(3)
N20	72(5)	26(3)	24(3)	0(2)	24(3)	7(3)
N13	39(4)	32(3)	29(3)	1(2)	-2(3)	2(3)
N24	59(4)	28(3)	27(3)	1(2)	5(3)	17(3)
N17	39(3)	30(3)	34(3)	-6(2)	-5(3)	-2(3)
C19	35(4)	30(3)	18(3)	1(2)	10(3)	2(3)
C3	29(3)	25(3)	24(3)	-4(2)	9(3)	-1(2)
C23	31(4)	25(3)	32(3)	-2(3)	10(3)	3(3)
C11	33(4)	23(3)	26(3)	1(2)	8(3)	3(3)
C15	25(3)	29(3)	30(3)	-2(3)	3(3)	-6(3)
C7	32(4)	21(3)	32(3)	-2(3)	9(3)	-2(3)
Mn2A	28.8(6)	20.2(5)	17.9(5)	0.2(4)	9.4(4)	-5.0(4)
Mn1A	30.5(6)	22.9(5)	19.2(5)	-0.8(4)	9.7(4)	4.6(4)
Mn3A	26.2(6)	20.1(5)	26.6(6)	-0.7(4)	6.3(4)	0.0(4)
O32A	36(3)	31(2)	34(3)	0(2)	14(2)	-2(2)
O27A	37(3)	39(3)	33(3)	-3(2)	17(2)	-3(2)
O38A	37(3)	42(3)	39(3)	-10(2)	19(2)	-11(2)
Mn1B	30.5(6)	22.9(5)	19.2(5)	-0.8(4)	9.7(4)	4.6(4)
Mn2B	28.8(6)	20.2(5)	17.9(5)	0.2(4)	9.4(4)	-5.0(4)
Mn3B	26.2(6)	20.1(5)	26.6(6)	-0.7(4)	6.3(4)	0.0(4)
O27B	37(3)	39(3)	33(3)	-3(2)	17(2)	-3(2)
O32B	36(3)	31(2)	34(3)	0(2)	14(2)	-2(2)
O38B	37(3)	42(3)	39(3)	-10(2)	19(2)	-11(2)

ESI Table S9. Bond lengths (Å) in compound **1**.

Atom	Atom	Bond Lentgh	Atom	Atom	Bond Length
Fe1	O2	1.998(4)	O28	Mn1A	1.601(5)
Fe1	O6	1.993(4)	O28	Mn1B	1.555(11)
Fe1	O10	2.002(4)	O29	Mn1A	1.608(4)
Fe1	O14	2.012(4)	O29	Mn1B	1.574(11)
Fe1	O18	1.991(4)	O30	Mn1A	1.585(5)
Fe1	O22	2.001(4)	O30	Mn1B	1.600(11)
O2	C3	1.282(7)	O40	Mn3A	1.610(5)
O6	C7	1.273(7)	O40	Mn3B	1.537(11)
O10	C11	1.273(7)	Mn1A	O27A	1.627(5)
O14	C15	1.282(8)	Mn1B	O27B	1.644(18)
O18	C19	1.277(7)	O33	Mn2A	1.607(4)
O22	C23	1.271(7)	O33	Mn2B	1.586(11)
N4	C3	1.329(8)	O34	Mn2A	1.592(4)
N5	C3	1.334(8)	O34	Mn2B	1.588(11)
N8	C7	1.325(9)	O35	Mn2A	1.600(4)
N9	C7	1.328(9)	O35	Mn2B	1.538(11)
N12	C11	1.318(8)	Mn2A	O32A	1.622(5)
N13	C11	1.320(8)	Mn2B	O32B	1.646(18)
N16	C15	1.319(9)	O37	Mn3A	1.600(5)
N17	C15	1.318(9)	O37	Mn3B	1.570(11)
N20	C19	1.331(8)	O39	Mn3A	1.611(5)
N21	C19	1.330(8)	O39	Mn3B	1.538(11)
N24	C23	1.324(9)	Mn3A	O38A	1.628(5)
N25	C23	1.320(9)	Mn3B	O38B	1.696(19)

ESI Table S10. Bond angles (°) in compound **1**.

Atom	Atom	Atom	Bond angle	Atom	Atom	Atom	Bond angle
O6	Fe1	O2	90.19(17)	N25	C23	N24	119.0(6)
O2	Fe1	O10	91.00(18)	O28	Mn1A	O29	110.9(2)
O2	Fe1	O14	91.30(18)	O30	Mn1A	O28	112.0(3)
O6	Fe1	O10	90.81(18)	O30	Mn1A	O29	110.2(2)
O6	Fe1	O14	87.90(18)	O28	Mn1A	O27A	107.8(3)
O18	Fe1	O10	91.05(19)	O29	Mn1A	O27A	108.4(2)
O18	Fe1	O14	90.33(18)	O30	Mn1A	O27A	107.4(3)
O18	Fe1	O22	91.53(18)	O28	Mn1B	O29	115.3(8)
O18	Fe1	O2	87.47(18)	O28	Mn1B	O30	113.7(8)
O6	Fe1	O22	90.87(18)	O29	Mn1B	O30	111.2(7)
O22	Fe1	O10	87.22(19)	O28	Mn1B	O27B	106.6(15)
O22	Fe1	O14	90.51(18)	O29	Mn1B	O27B	106.2(15)
O2	Fe1	O22	177.94(17)	O30	Mn1B	O27B	102.6(15)
O18	Fe1	O6	177.03(17)	O34	Mn2A	O33	111.5(2)
O10	Fe1	O14	177.38(18)	O35	Mn2A	O33	110.1(3)
C3	O2	Fe1	132.0(4)	O34	Mn2A	O35	111.0(3)
C7	O6	Fe1	133.6(4)	O33	Mn2A	O32A	108.7(2)
C11	O10	Fe1	133.7(4)	O34	Mn2A	O32A	108.3(3)
C15	O14	Fe1	130.5(4)	O35	Mn2A	O32A	107.0(3)
C19	O18	Fe1	131.7(4)	O33	Mn2B	O34	112.9(7)
C23	O22	Fe1	131.8(4)	O35	Mn2B	O33	114.6(8)
O2	C3	N4	121.6(6)	O35	Mn2B	O34	114.6(8)
O2	C3	N5	118.8(6)	O33	Mn2B	O32B	104.0(15)
O6	C7	N8	122.1(6)	O35	Mn2B	O32B	104.7(15)
O6	C7	N9	118.3(6)	O37	Mn3A	O39	111.2(3)
O10	C11	N12	122.0(6)	O37	Mn3A	O40	111.0(3)
O10	C11	N13	119.0(6)	O40	Mn3A	O39	110.6(3)
O14	C15	N16	122.2(6)	O37	Mn3A	O38A	108.3(3)
O14	C15	N17	118.4(6)	O39	Mn3A	O38A	108.0(3)
O18	C19	N21	119.4(6)	O40	Mn3A	O38A	107.6(3)
O18	C19	N20	121.5(6)	O39	Mn3B	O37	116.9(8)
O22	C23	N25	119.6(6)	O40	Mn3B	O37	116.7(8)
O22	C23	N24	121.3(6)	O40	Mn3B	O39	118.8(8)
N4	C3	N5	119.5(6)	O37	Mn3B	O38B	99.4(14)
N8	C7	N9	119.6(6)	O39	Mn3B	O38B	100.0(15)
N12	C11	N13	119.0(6)	O40	Mn3B	O38B	98.2(14)
N17	C15	N16	119.3(6)				
N21	C19	N20	119.1(6)				

ESI Table S11. Torsion angles (°) in compound **1**.

Atom	Atom	Atom	Atom	Torsion angle
Fe1	O6	C7	N9	158.9(5)
Fe1	O2	C3	N5	157.1(5)
Fe1	O10	C11	N13	154.5(5)
Fe1	O14	C15	N17	156.9(5)
Fe1	O18	C19	N21	149.5(5)
Fe1	O22	C23	N25	148.6(5)
Fe1	O6	C7	N8	-20.7(10)
Fe1	O2	C3	N4	-21.1(9)
Fe1	O10	C11	N12	-25.6(11)
Fe1	O14	C15	N16	-24.6(10)
Fe1	O18	C19	N20	-31.3(10)
Fe1	O22	C23	N24	-30.6(10)

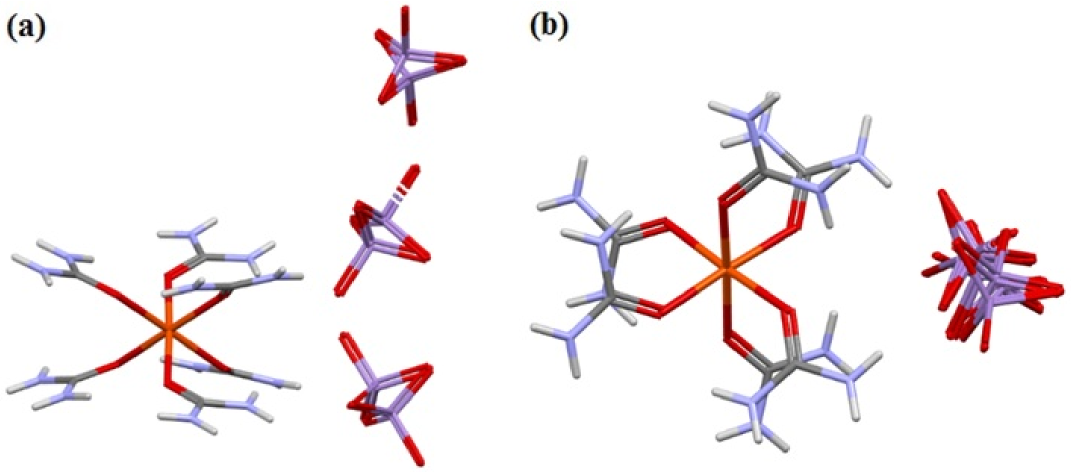

ESI Figure S5. Coordination geometry of the [hexakis(urea-O)iron(III)] tripermanganate. (**a**) shows alternate orientations of the three permanganate ions, referred to as 'up’ and 'down’. In the figure, the major orientation ‘A’ is pointing upward. (**b**) is a view from an approximate orientation of the pseudo $\bar{3}$ axes parallel to axis *a*.

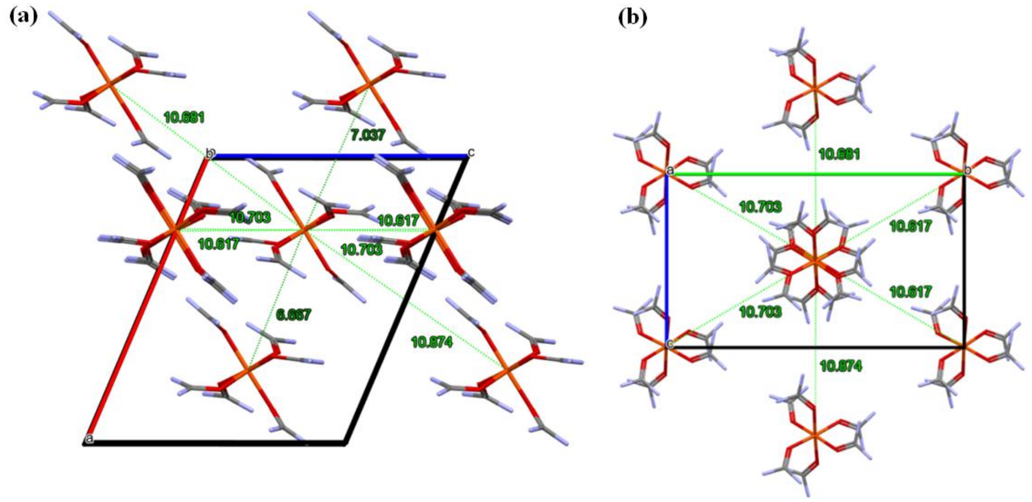

ESI Figure S6. The Fe···Fe distances in the structure. **a)** View along unit cell axis *b*. The vectors between the closest Fe-Fe atom pairs are parallel with unit cell axis *a*. **b)** The view along unit cell axis *a* shows the distances for pseudo pseudo $\bar{3}$ axis related complexes surrounding the central complex. (The permanganate ions are not shown.)

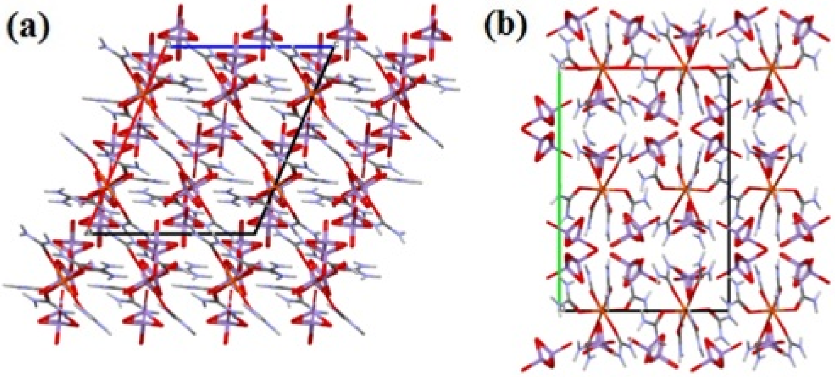

ESI Figure S7. Crystal packing of the complex. View along unit cell **a)** axis *b*, and **b)** axis *c*.

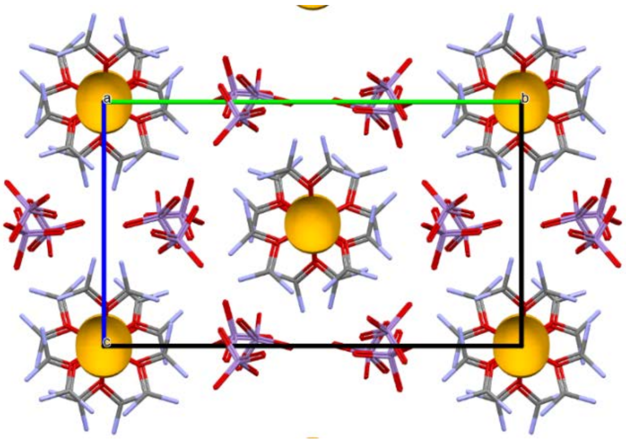

ESI Figure S8. Potential solvent-accessible voids in the unit cell of the complex. (The voids were calculated for the unit cell containing only the major orientation of the permanganate ions).

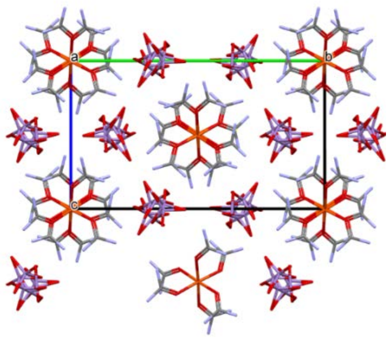

hexakis(urea)-iron(III)) tris(permanganate) (compound 1)

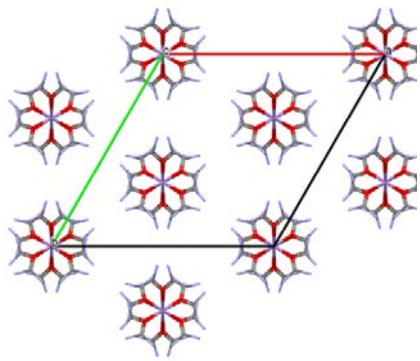

hexakis(urea)-manganese(III) triperchlorate (CSD id: BOPWEI, space group: $R\bar{3}c$)

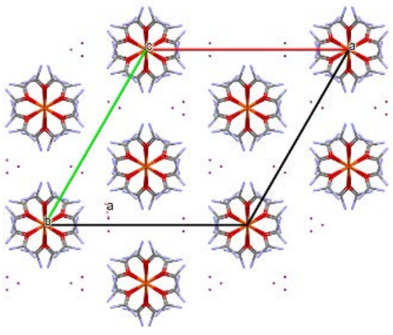

hexakis(urea)-iron(III) di-iodide (CSD id: WITQEV, space group: $R\bar{3}$)

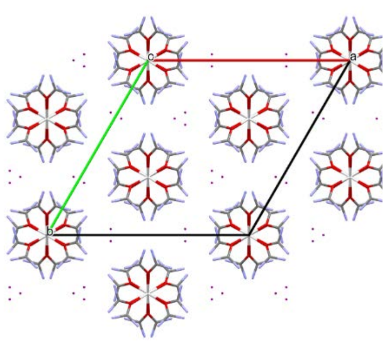

hexakis(urea)-scandium(III) tris(iodide) (CSD id: LIVVIY , space group: $R\bar{3}c$)

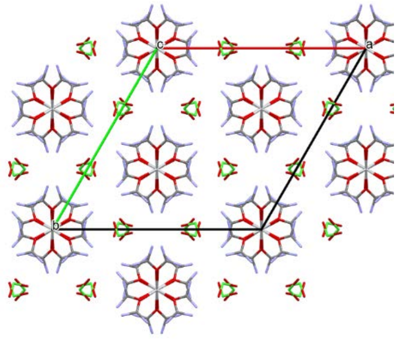

hexakis(urea)-titanium(III) triperchlorate (CSD id: TIUERA, space group: $R\bar{3}c$)

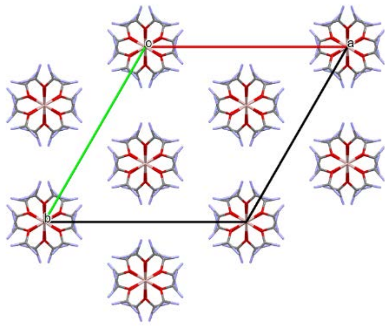

hexakis(urea-O)-aluminium perchlorate (CSD id: URALPC, space group: $R\bar{3}c$)

ESI Figure S9. Crystal packing of compound **1** compared to some structures with similar packing topologies. Space group symmetries and CSD [30] reference codes are listed.

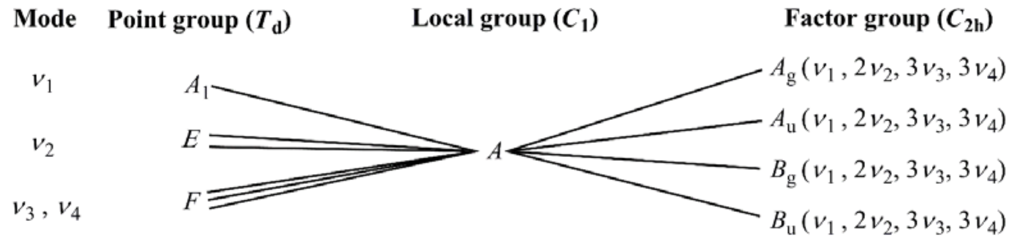

ESI Figure S10. The four normal modes of the tetrahedral permanganate anions in compound **1**

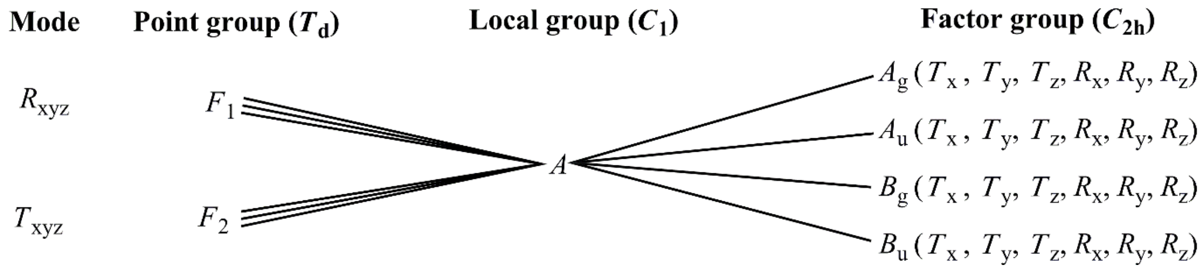

ESI Figure S11. The external modes of permanganate ion in compound **1**.

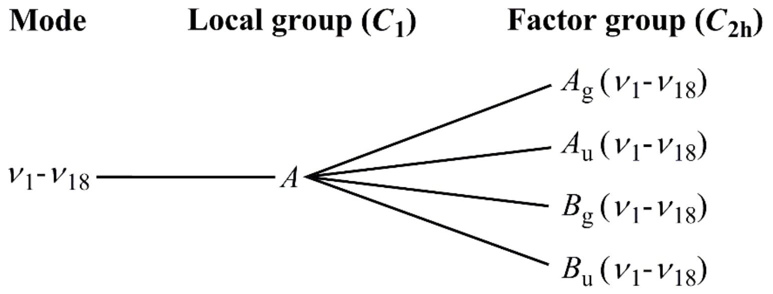

ESI Figure S12. The normal modes of urea in compound **1**.

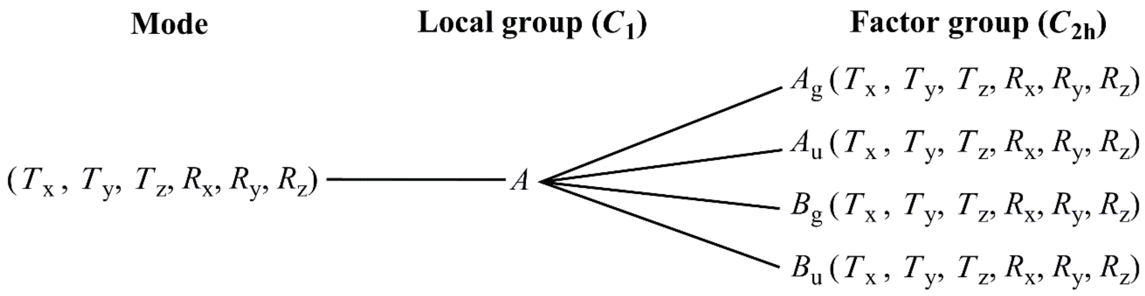

ESI Figure S13. The external modes of urea in compound 1.

ESI Table S12. The assignments of the vibrational modes of Compound **1.**

$[Fe(urea\text{-}O)_6](MnO_4)_3$				Solid urea [37]	Gaseous urea [37]	Assignment
IR, 298 K	IR, 100 K	Raman, 298 K	Raman, 123 K	IR	IR	
3469s, 3455sh	3472s, 3443m, 3415s	-	3400vw	3450,3444	3548, 3548	$\nu_{as}(NH_2)$
3364m, 3348m	3367m, 3341m	3340w	3339w	3349,3341	3442,3437	$\nu_s(NH_2)$
3231w, 3182vw, 3129vw	3232w, 3147vw, 3124vw					
<1682*	<1681*	1679m	1673m	1683	1592	$\delta_s(NH_2)$
1628vs	1626vs	1622vw	1622vw	1625	1603	$\delta_{as}(NH_2)$
1549s,1533s, 1503m	1550s, 1532s, 1509m	1545w, 1501w	1545vw,1501vw	1601	1735	$\nu(C=O)$
1465vw,1446w,	1476vw,1465w,			1466	1388	$\nu_{as}(C\text{-}N)$
1406w,1361vw	1403w	1388w,1352w	1387,1358			Two-phonon bands, $\nu_s(CN)+\omega_{as}(NH_2)$; $\rho_{as}(NH_2)+\omega_{as}(NH_2)$
1143m	1145m	1138vw	1138vw	1153	1155	$\rho_s(NH_2)$
1036m	1039m	1068w, 1041w	1070w,1040w	1057	1029	$\rho_{as}(NH_2)$
-	-	-	-	1003	936	$\nu_s(CN)$
772vs,760vw	777vs,760vw	774s, 760s	774s,759s	789	786	$\pi(CO)$

680vw	711vw,689vw	680sh	680sh	721	517	$\tau_{as}(NH)$
611w, 572vw	611w, 581vw	612s,588m	614vs	569	568	$\delta(C=O)$
537w	545w	540sh,480w	538sh, 481w	530	467	$\delta(C-N)$
476sh, 468w, 441vw, 430sh, 407sh, 395w	440vw, 418vw,414vw			509	423	$\omega_{as}(NH_2)$
317w, 285sh. 247w, 189vw. 168vw, 124vw, 110vw	307w, 240w, 209sh, 190vw, 160vw, 131vw	307w, 211sh, 197sh, 160sh, 120w	306w, 213sh, 198sh, 165sh, 119m			

vs-very strong; s-strong; m-medium; w-weak; vw-very weak; sh-shoulder

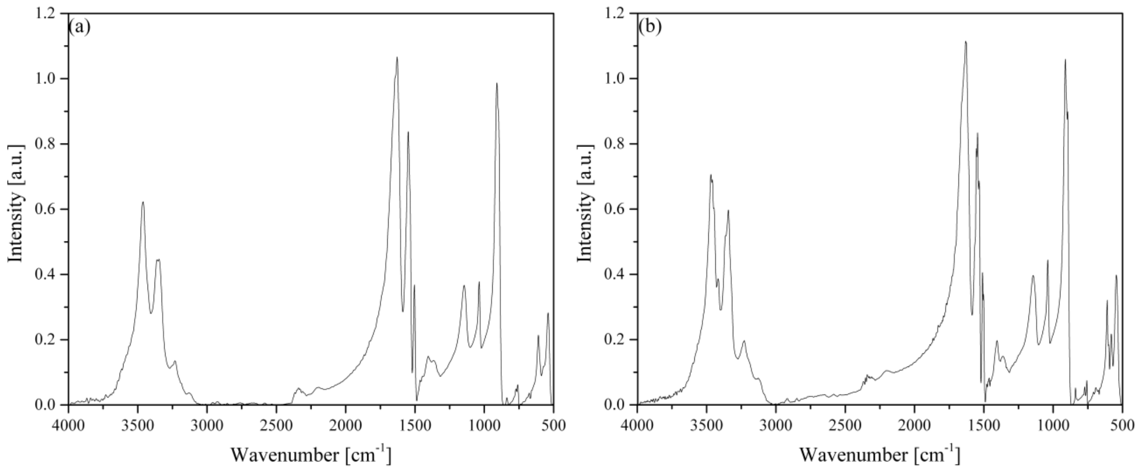

ESI Figure 14. The (a) room temperature and (b) liquid nitrogen temperature IR spectra of compound **1.**

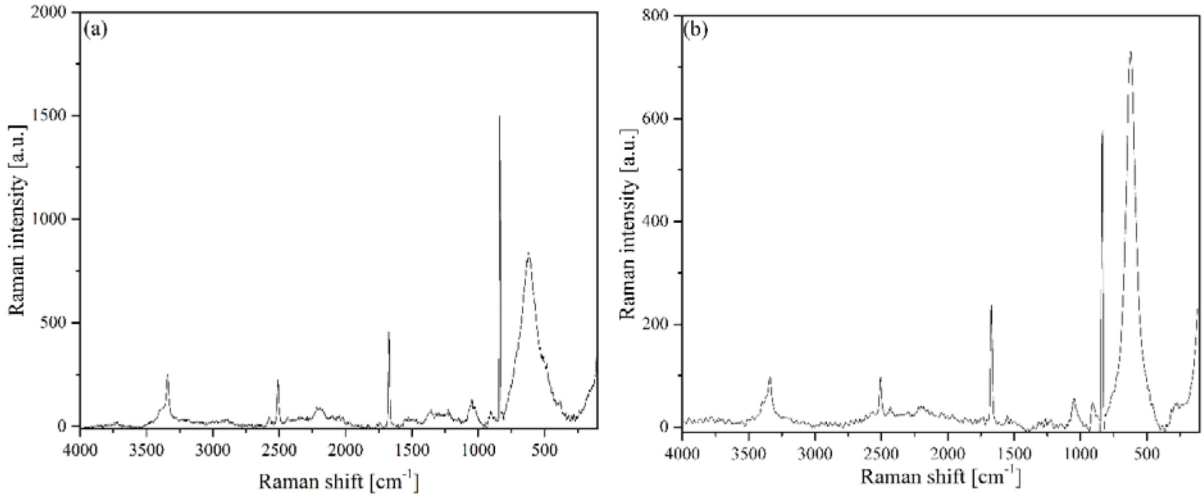

ESI Figure 15. The (a) room temperature and (b) liquid nitrogen temperature Raman spectra of compound **1.**

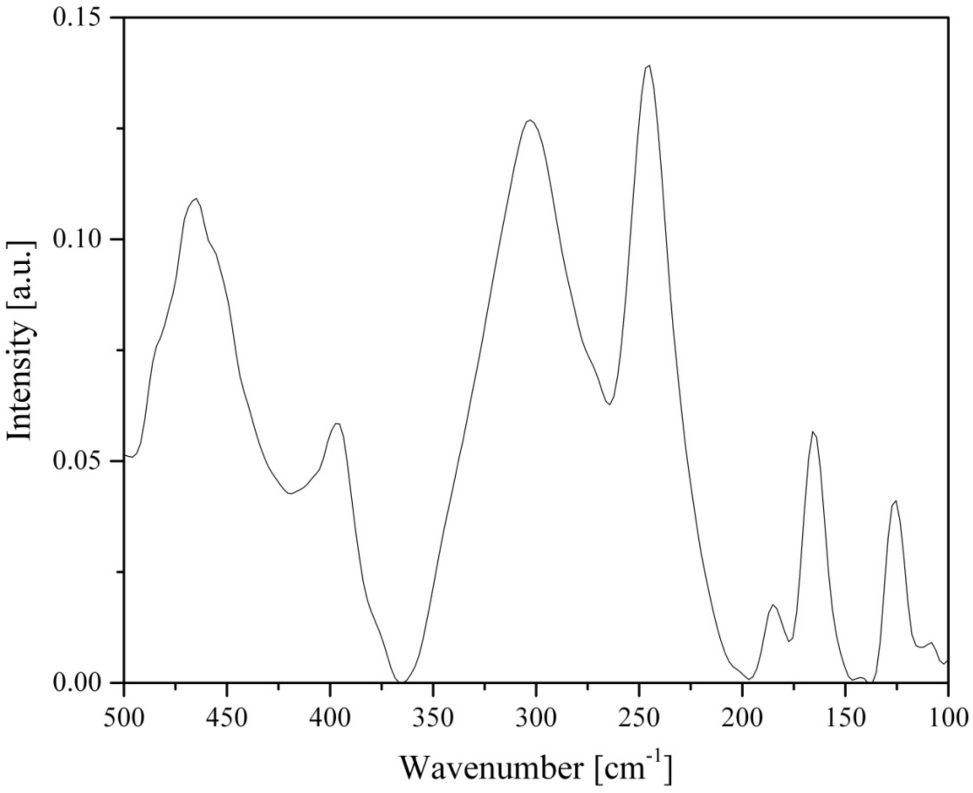

ESI Figure S16. The far-IR spectrum of compound **1** at room temperature.

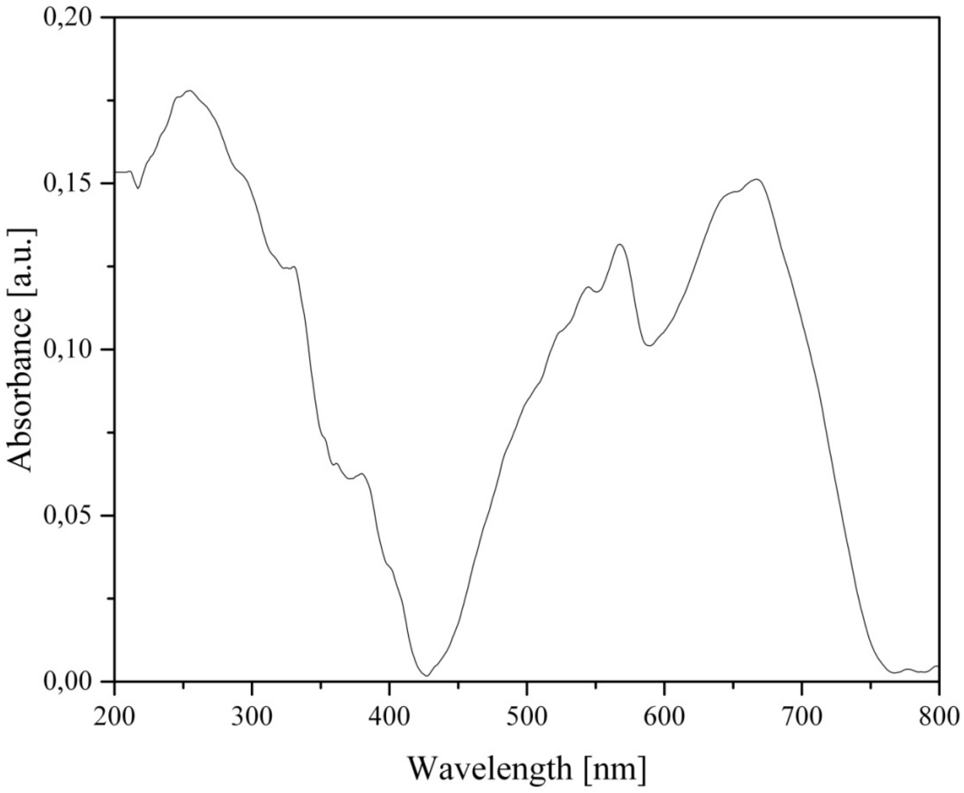

ESI Figure S17. The UV-VIS spectrum of compound **1**.

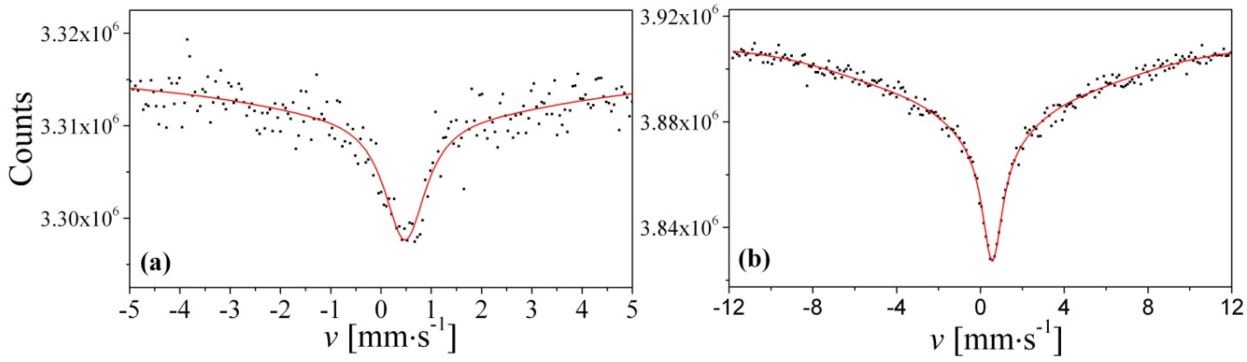

ESI Figure S18. The (a) room temperature and (b) liquid nitrogen temperature Mössbauer spectra of compound **1**.

ESI Table S13. The DFT-optimized structure of the [hexakis(urea-O)iron(III)] cation in Cartesian coordinates (Å).

Coordinates from ORCA-job Fe3-urea6-BP86-opt-CPCM			
Fe	0.000000000	0.00000000	0.000000000
O	0,1501592	1,688254	1,1282739
N	-0,4893589	3,7807622	1,7056743
H	0,4314895	4,11429	1,4425539
H	-1,1563015	4,4456425	2,0849273
N	-1,9947976	2,0137439	1,8555892
H	-2,1593912	1,0075772	1,7751994
H	-2,729803	2,6158073	2,2122068
C	-0,7760933	2,4795998	1,5590944
O	-1,5294606	-0,712546	1,1327544
N	-3,0264372	-2,3086598	1,7073403
H	-3,7735338	-1,6787962	1,435906
H	-3,2719103	-3,2216052	2,0768989
N	-0,7450765	-2,7364822	1,8561546
H	0,2097908	-2,3788912	1,7792939
H	-0,9018713	-3,6738619	2,2122156
C	-1,7548529	-1,9103182	1,5606227
O	1,3841122	-0,9677778	1,1310949
N	3,5152294	-1,4640144	1,7053047
H	3,3482633	-2,4229523	1,4201549
H	4,4284851	-1,2195555	2,0751194
N	2,7389297	0,7231968	1,8650017
H	1,9483999	1,3680418	1,7925622
H	3,6262416	1,0559286	2,22864
C	2,5328354	-0,5633154	1,562215
O	-0,1506676	-1,6882608	-1,1278224
N	0,4885096	-3,7810914	-1,7045898
H	-0,4335259	-4,1138192	-1,4446554
H	1,1559681	-4,4464261	-2,0821433
N	1,9952812	-2,0149747	-1,851668
H	2,1594615	-1,0086858	-1,7721778
H	2,7293577	-2,616608	-2,2109439
C	0,7756316	-2,4801486	-1,5574916
O	1,5286504	0,7128971	-1,132443
N	3,0253527	2,3082649	-1,7098952
H	3,7728419	1,6762838	-1,4445765
H	3,270551	3,2195472	-2,0837301
N	0,7439647	2,7369711	-1,8552964
H	-0,2108813	2,3793432	-1,7778872
H	0,9007269	3,6730442	-2,2148415
C	1,753856	1,910324	-1,5615506
O	-1,3848977	0,9678629	-1,1301427
N	-3,5155171	1,4644733	-1,7057986
H	-3,3467945	2,4246323	-1,4258616
H	-4,4284487	1,2202363	-2,076561
N	-2,7407531	-0,7235488	-1,8613457
H	-1,9504402	-1,3686325	-1,7886443
H	-3,6272654	-1,0555024	-2,227681
C	-2,5336768	0,5634809	-1,5611084

ESI Table S14. The Mössbauer parameters of compound **1** obtained with the Blume-Tjon model.

	$[Fe(urea)_6](MnO_4)_3$	
***T* (K)**	**298**	**80**
δ (mm·s^{-1})	0.412 (± 0.024)	0.501 (± 0.023)
***B* (T)**	45 (± 7.1)	49 (± 2.5)
V_{zz} (·10^{21} v·m^{-2})*	-0.762	-0.762
ETA*	0.232	0.232
Jump up rate	8.40 (± 0.15)	8.57 (± 0.03)
Γ (mm·s^{-1})	0.626 (± 0.091)	0.875 (± 0.034)

*** Fixed parameters**

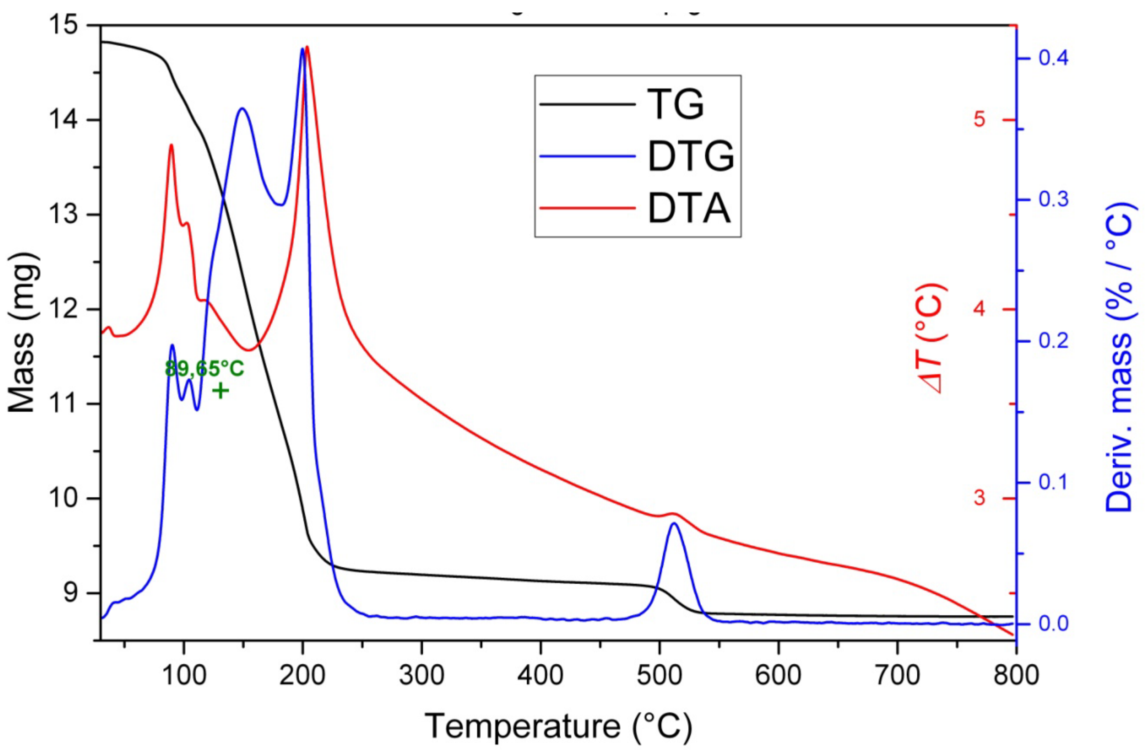

ESI figure S19. TG-DTG curve of compound **1** in air.

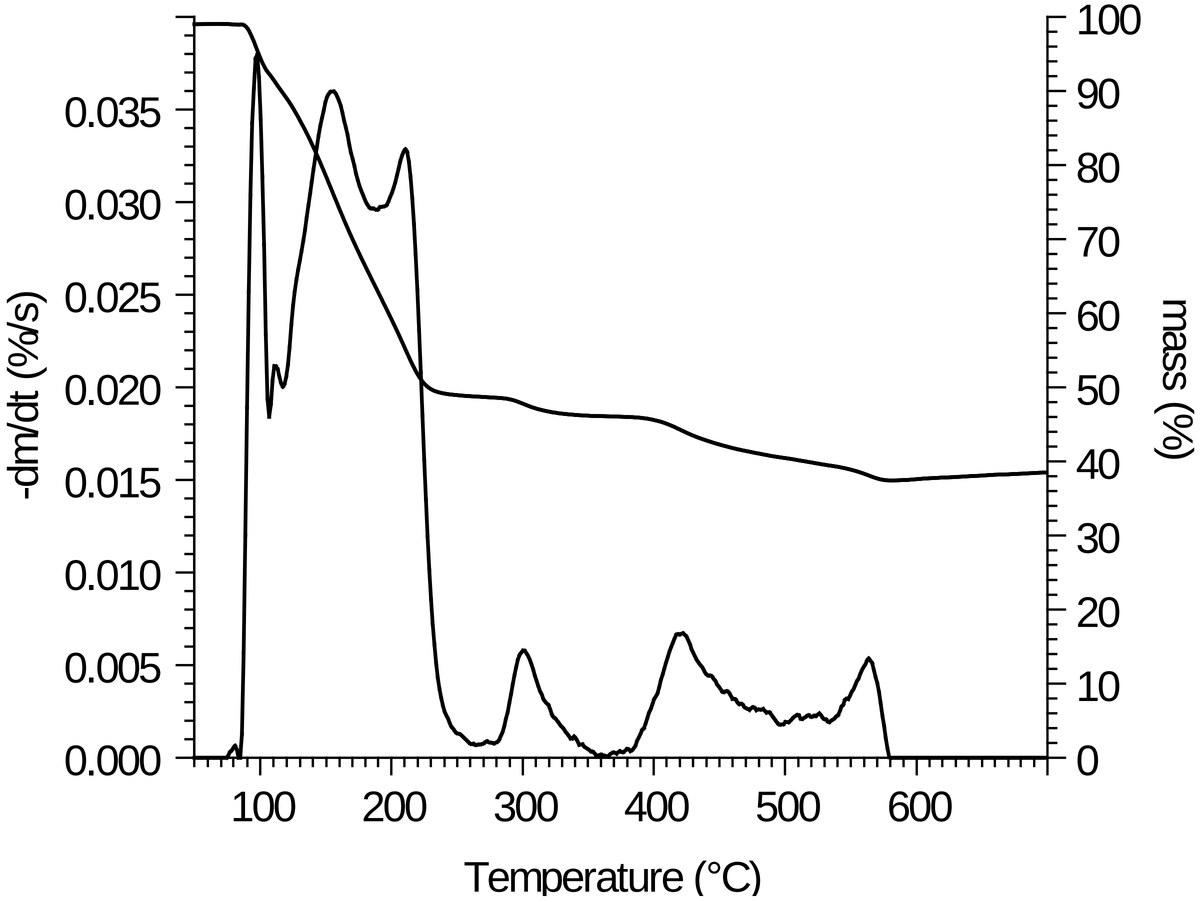

ESI figure S20. TG-DTG curve of compound **1** in Ar.

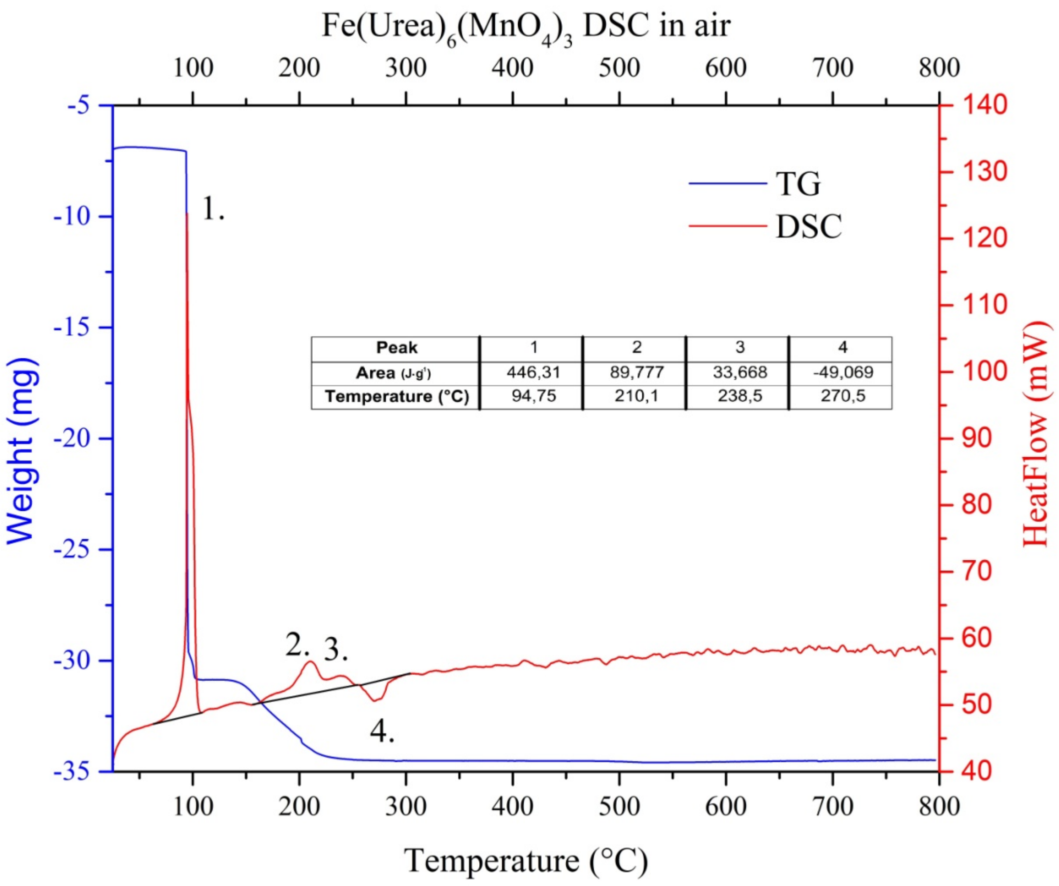

ESI figure S21. DSC curve of compound **1** in air.

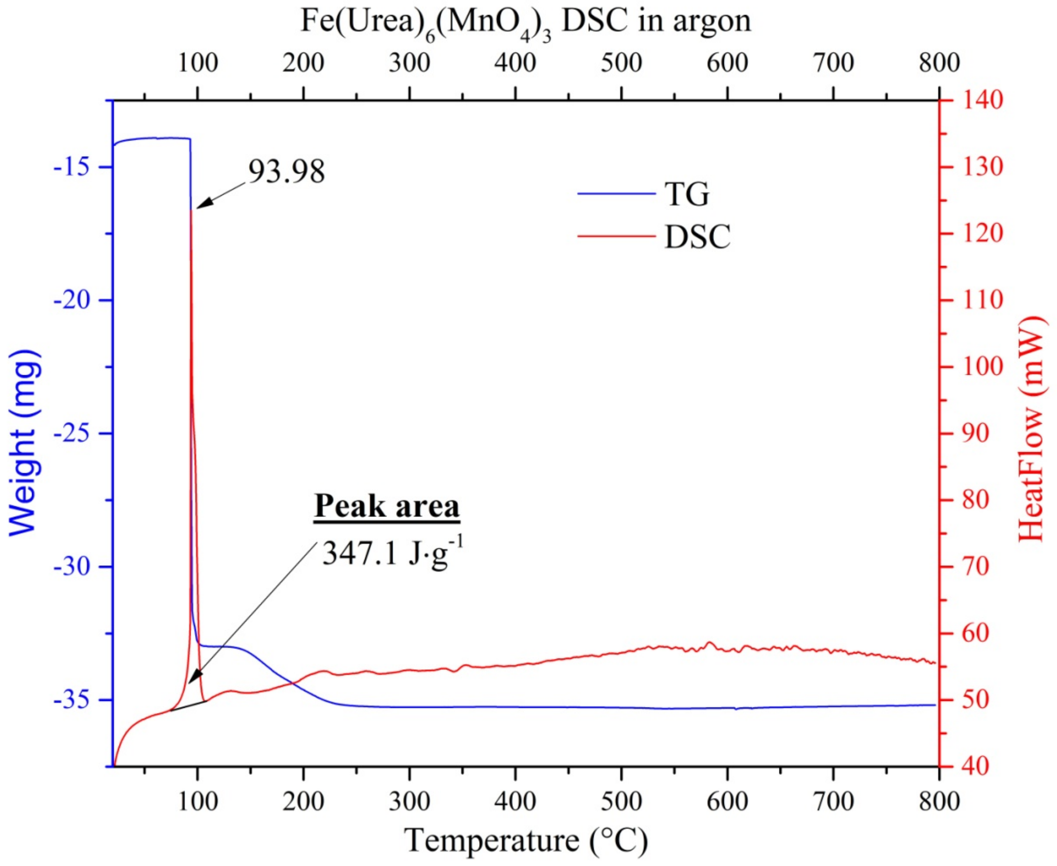

ESI figure S22. DSC curve of compound **1** in argon.

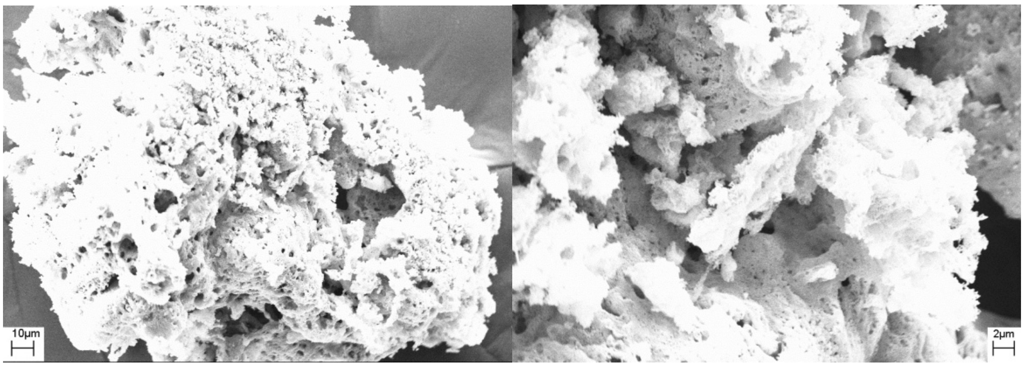

ESI Figure S23. The SEM image of decomposition product formed from Compound **1** after heating at 800 °C in air.

ESI Table S15. The Mössbauer parameters of the intermediate phases formed at 550 °C (30 min), 800 °C (30 min) and 800 °C (24 h) in air.

	550 °C				800 °C – 30 min				800 °C – 24 h			
Component	**Bixbyite I.**		**Bixbyite II.**		**Bixbyite I.**		**Bixbyite II.**		**Bixbyite I.**		**Bixbyite II.**	
Doublet	I.	II.	I.	II.	I.	II.	I.	II.	I.	II.	I.	II.
Relative area (%)	27.41	72.59	---	---	37.16	49.64	6.19	7.01	17.41	26,47	20.99	35.13
$\boldsymbol{\delta}$ (mm·s^{-1})	0.349 (±0.003)	0.368 (±0.001)	---	---	0.350 (±0.002)	0.378 (±0.001)	0.363 (±0.009)	0.373 (±0.003)	0.357 (±0.003)	0.376 (±0.003)	0.358 (±0.003)	0.3745 (±0.002)
$\boldsymbol{\Delta}$ (mm·s^{-1})	0.578 (±0.011)	1.153 (±0.006)	---	---	0.453 (±0.023)	1.151 (±0.014)	0.687 (±0.107)	1.418 (±0.018)	0.365 (±0.068)	1.018 (±0.047)	0.564 (±0.085)	1.296 (±0.034)
$\boldsymbol{\Gamma}$ (mm·s^{-1})	0.350 (±0.014)	0.418 (±0.007)	---	---	0.347 (±0.012)	0.368 (±0.010)	0.316 (±0.187)	0.219 (±0.032)	0.243 (±0.055)	0.296 (±0.085)	0.263 (±0.101)	0.297 (±0.027)

ESI table S16. The Mössbauer parameters of the decomposition product formed from compound **1** at 800 °C in inert atmosphere.

Component	**Doublet I.**	**Doublet II.**	**Doublet III.**	**Doublet IV.**
Relative area (%)	45.25	23.57	23.66	7.52
δ ($mm \cdot s^{-1}$)	1.077 (±0.004)	1.096 (±0.051)	0.177 (±0.064)	0.441 (±0.034)
Δ ($mm \cdot s^{-1}$)	0.415 (±0.019)	0.817 (±0.125)	0.397 (±0.038)	0.517 (±0.104)
Γ ($mm \cdot s^{-1}$)	0.382 (±0.023)	0.543 (±0.049)	0.638 (±0.051)	0.338 (±0.144)

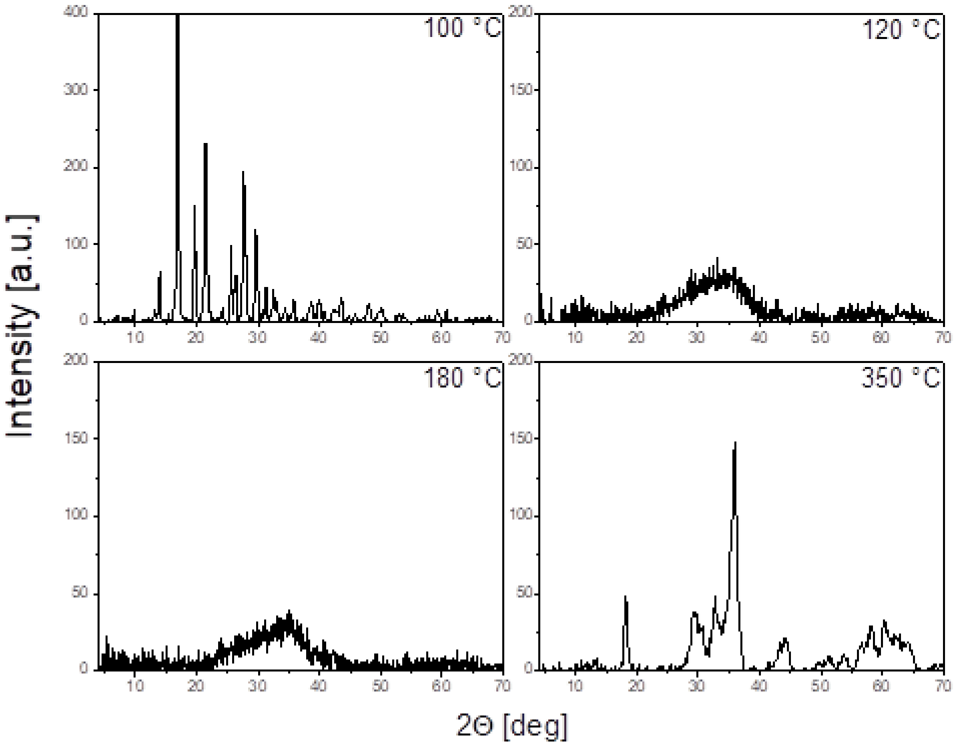

ESI figure S24. XRD patterns of the decomposition products of compound **1** in air between 120 °C and 350 °C.

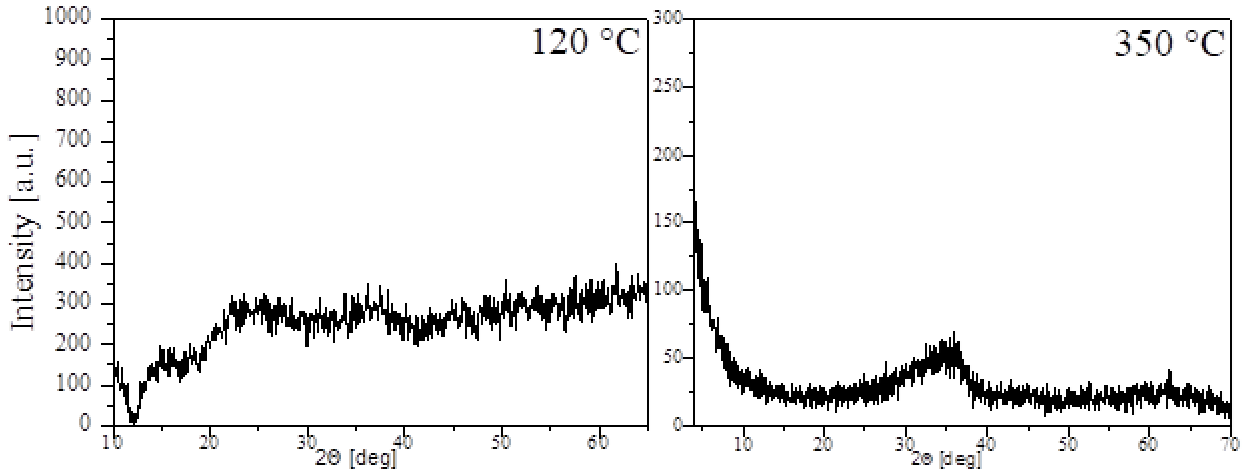

ESI figure S25. XRD patterns of the decomposition products of compound **1** in inert atmosphere at 120 °C and 350 °C.

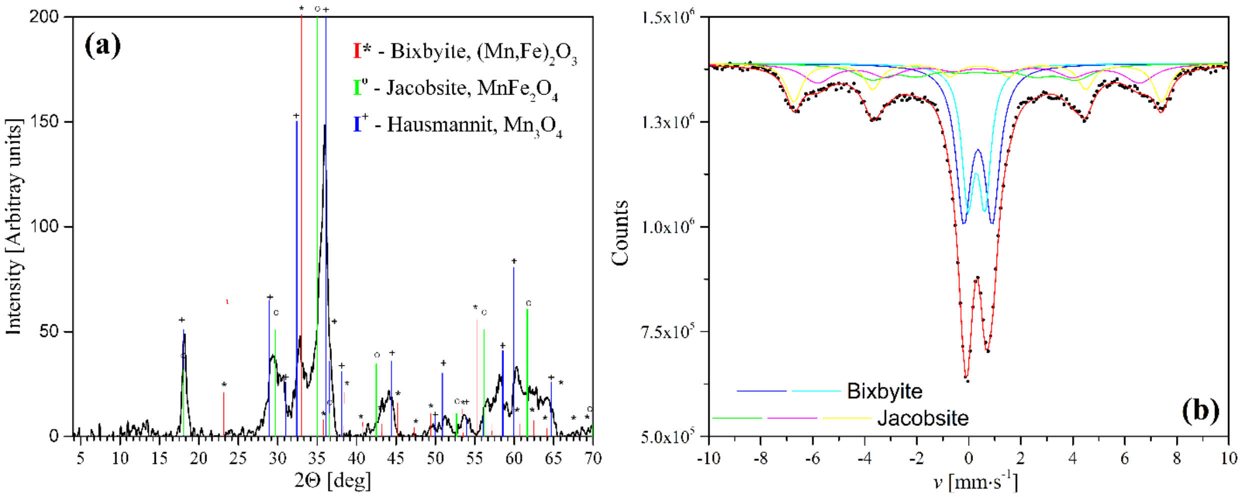

ESI S26. The (a) powder XRD and (b) Mössbauer spectrum of the decomposition product formed from compound **1** after heating at 350 °C in air.

ESI table S17. Mössbauer parameters of the intermediary phases formed at 350 °C in air.

Component	**Doublet I.**	**Doublet II.**	**Magnetic I.**	**Magnetic II.**	**Magnetic III.**
Relative area (%)	31.25	21.99	13.85	15.69	16.89
$\boldsymbol{\delta}$ (mm·s^{-1})	0.352 (±0.001)	0.292 (±0.001)	0.249 (±0.009)	0.385 (±0.006)	0.374 (±0.002)
$\boldsymbol{\Delta}$ (mm·s^{-1})	1.099 (±0.005)	0.658 (±0.003)	-0.075 (±0.017)	-0.024 (±0.012)	-0.059 (±0.004)
$\boldsymbol{\Gamma}$ (mm·s^{-1})	0.702 (±0.003)	0.554 (±0.004)	1.58 (±0.04)	1.38 (±0.03)	0.727 (±0.009)
B (T)	---	---	24.4 (±0.07)	38.5(±0.07)	44.0 (±0.02)

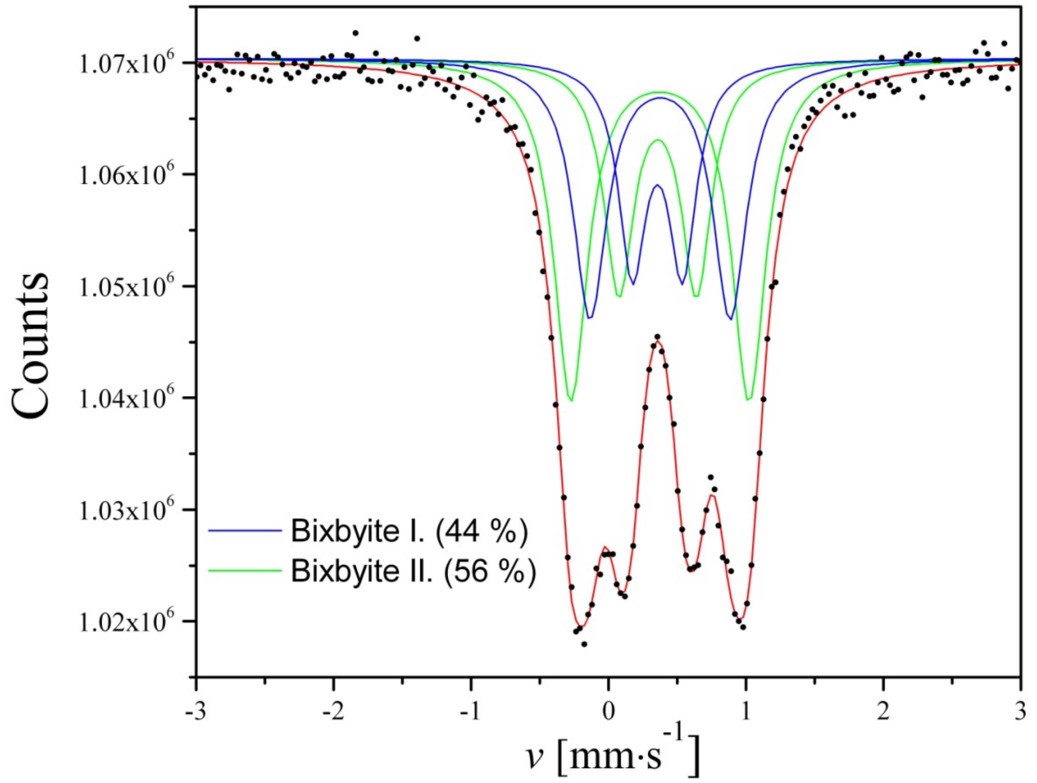

ESI Figure S27. Room temperature Mössbauer spectrum of the material that formed upon heat treatment at 800 °C in air for 24 h.

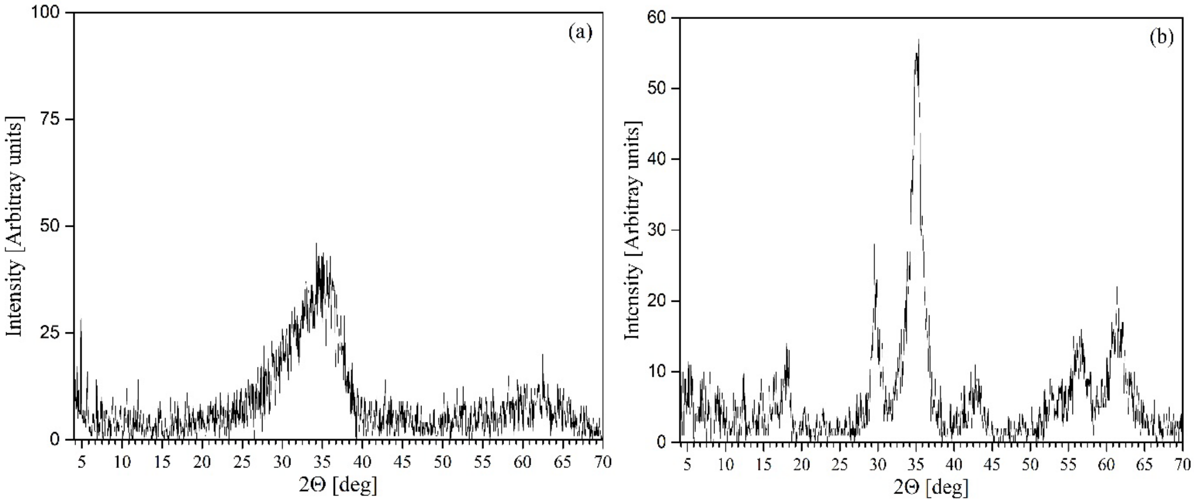

ESI Figure S28. Powder XRD of the (a) freshly prepared an (b) one week old (stored at room temperature in air) intermediary phases formed at 350 °C in inert atmosphere.

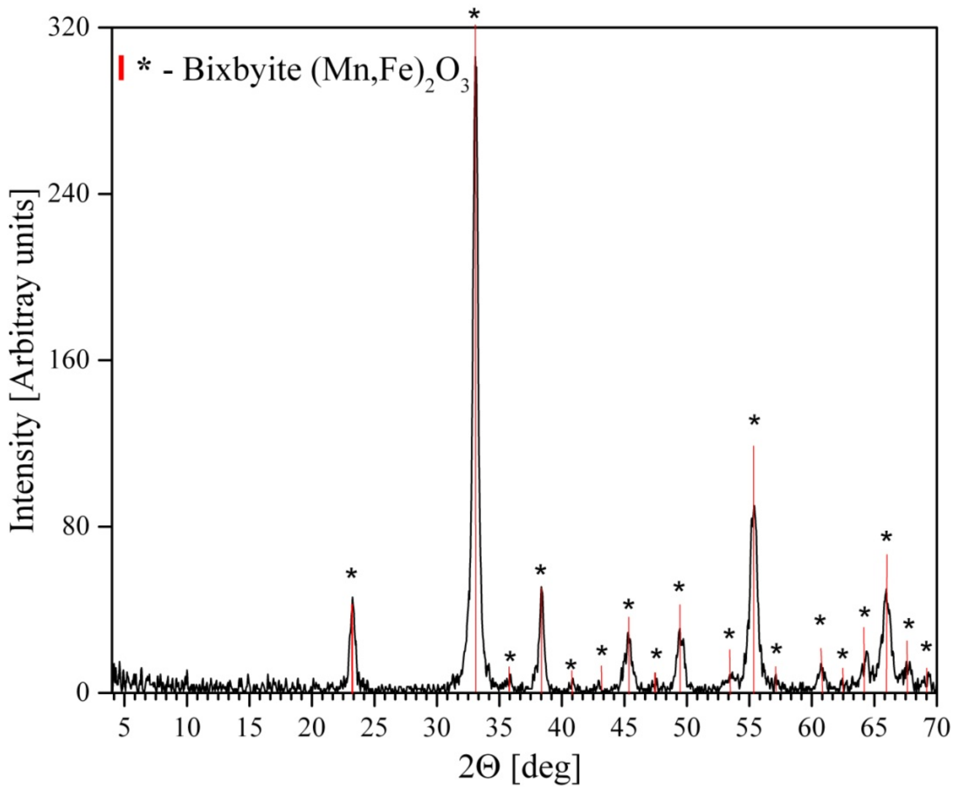

ESI Figure S29. Powder XRD of the intermediary phases formed at 550 °C in air.

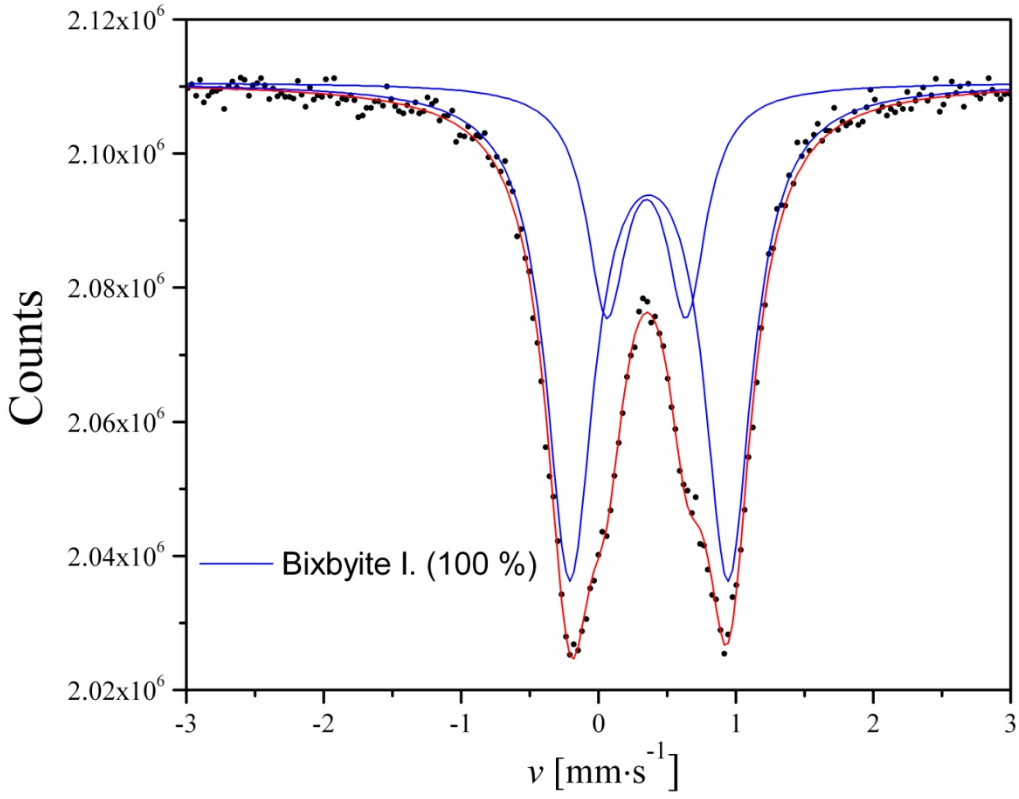

ESI Figure S30. Room temperature Mössbauer spectrum of the intermediary phases formed upon heat treatment at 550 °C in air.

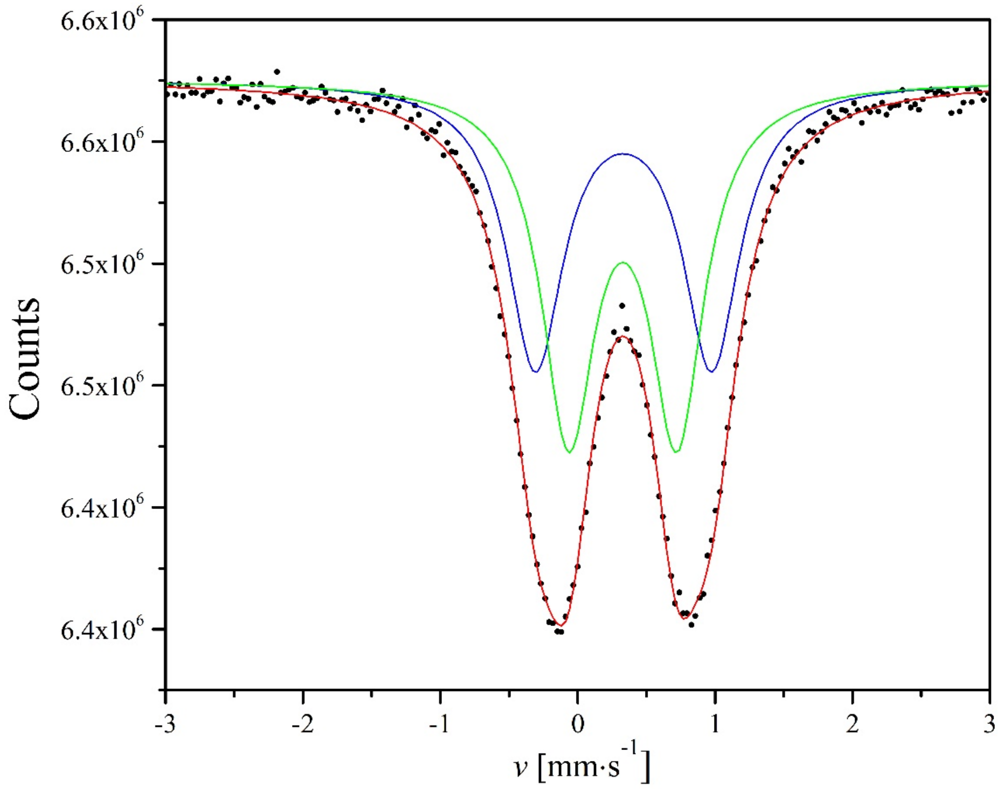

ESI Figure S31. Room temperature Mössbauer spectrum of the intermediary phases formed at 350 °C in inert atmosphere.

ESI Table S18. The Mössbauer parameters of the of the intermediary phases formed at 350 °C in inert atmosphere.

Component	**Doublet I.**	**Doublet II.**
Relative area (%)	46.29	53.71
$\boldsymbol{\delta}$ (mm·s^{-1})	0.333 (±0.002)	0.328 (±0.002)
$\boldsymbol{\Delta}$ (mm·s^{-1})	1.283 (±0.020)	0.789 (±0.002)
$\boldsymbol{\Gamma}$ (mm·s^{-1})	0.498 (±0.015)	0.473 (±0.012)

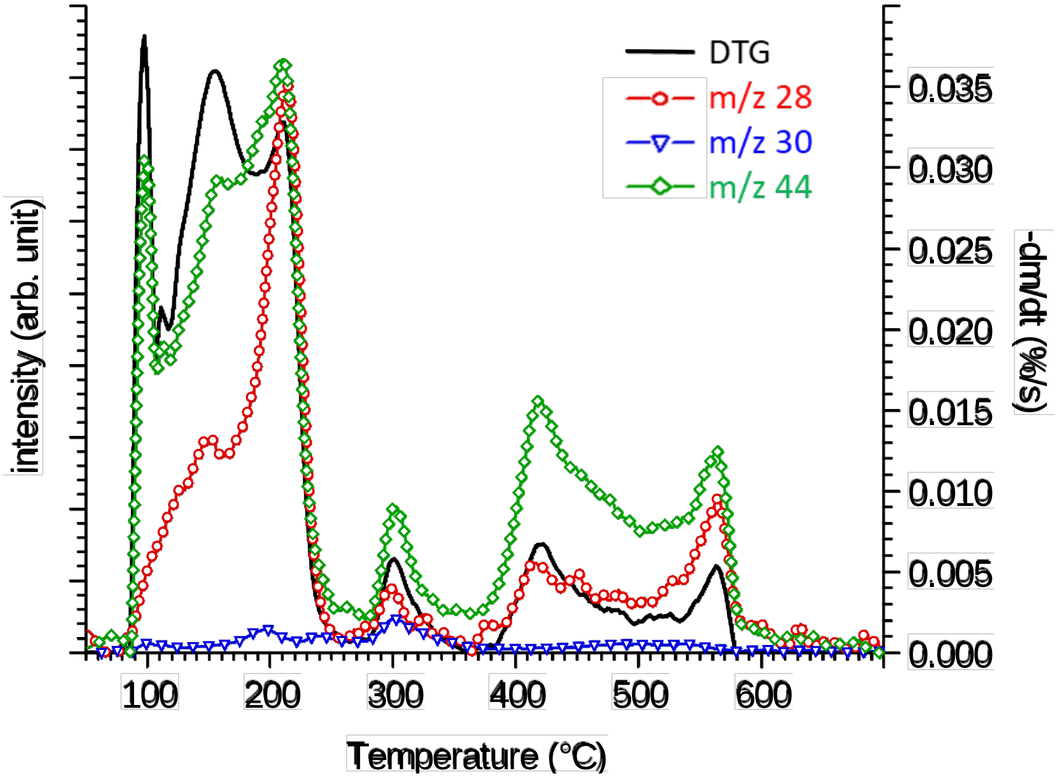

ESI Figure S32. The TG-MS ion curves showing *m*/*z* = 28 (CO, N_2), *m*/*z* = 30 (NO) and *m*/*z* = 44 (CO_2).

ESI Table S19. Elemental composition of the decomposition product formed from compound **1** after heating at 800 °C in inert atmosphere (given in atomic %).

**Fe (%)**	**Mn (%)**	**O (%)**	**C (%)**
14,8	27,1	54,6	3,5

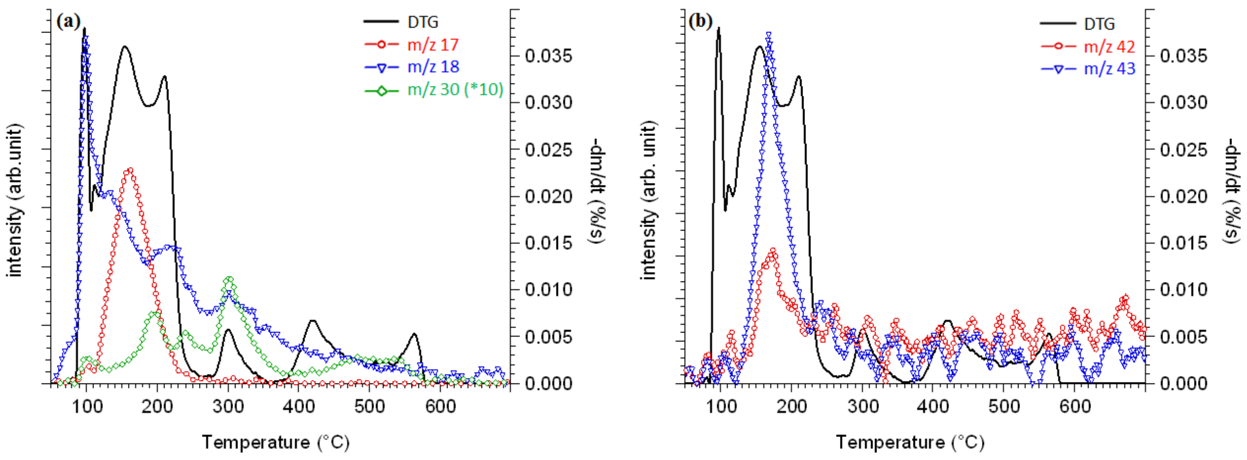

ESI Figure S33. TG/MS ion intensity curves of oxidation (**(a)** H_2O, *m*/*z*=18 and NO, *m*/*z*=30) and ligand decomposition products (**(a)** NH_3, *m*/*z*=17; **(b)** HNCO/NCO *m*/*z*=43/42) formed during the thermal decomposition of compound **1** in Ar atmosphere.

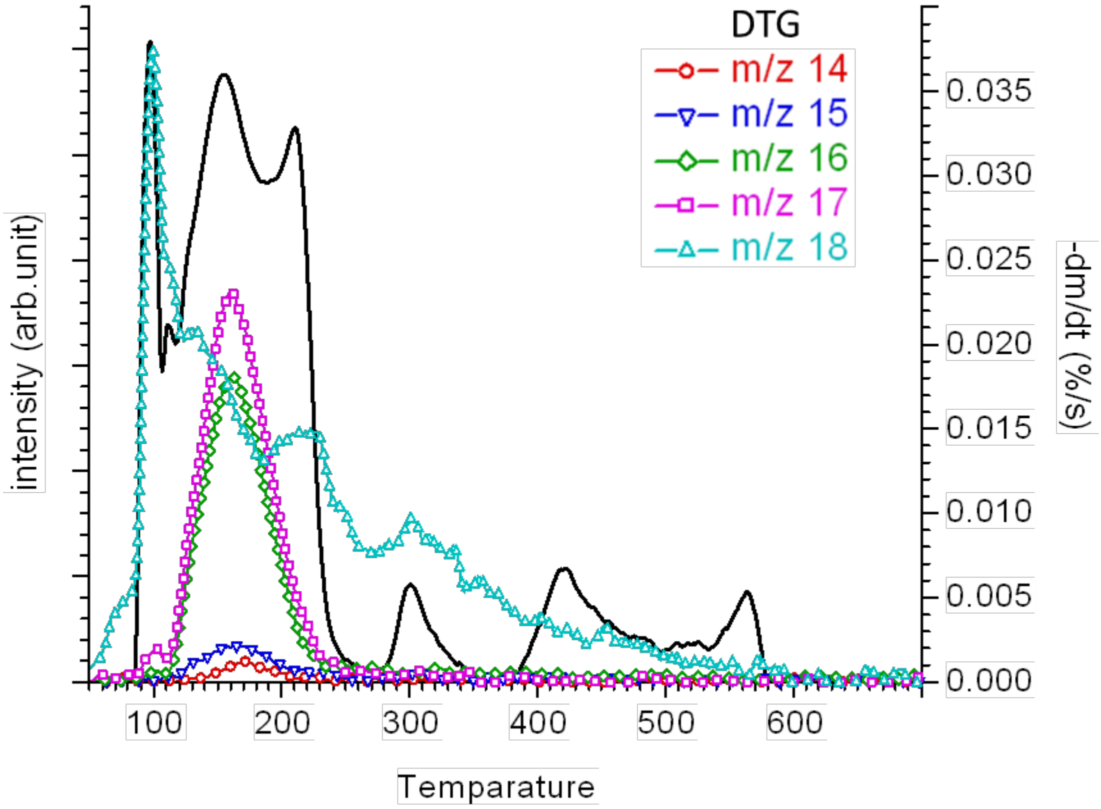

ESI Figure S34. The TG-MS ion curves showing *m*/*z* =14,16,17,18.

ESI Table S20. *Catalytic effect of the thermal decomposition products of compound **1** in CO_2 hydrogenation.*

Sample ID	Heat treatment condition	T_{transf} (K)	Max. Conversion (%)	CO (%)	CH_4 (%)	C_2H_6 (%)	C_3H_8 (%)
BK2001	100 °C (Air)	373	52.2	73.4	25	1.6	---
BK2002	120 °C (Air)	393	51.8	67.8	29.8	2.4	---
BK2003	180 °C (Air)	453	50.3	60.6	34.3	4.2	0.86
BK2004	350 °C (Air)	623	57.6	84.8	12.1	3.1	---
BK2006	550 °C (Air)	723	54.6	92.1	7.9	---	---

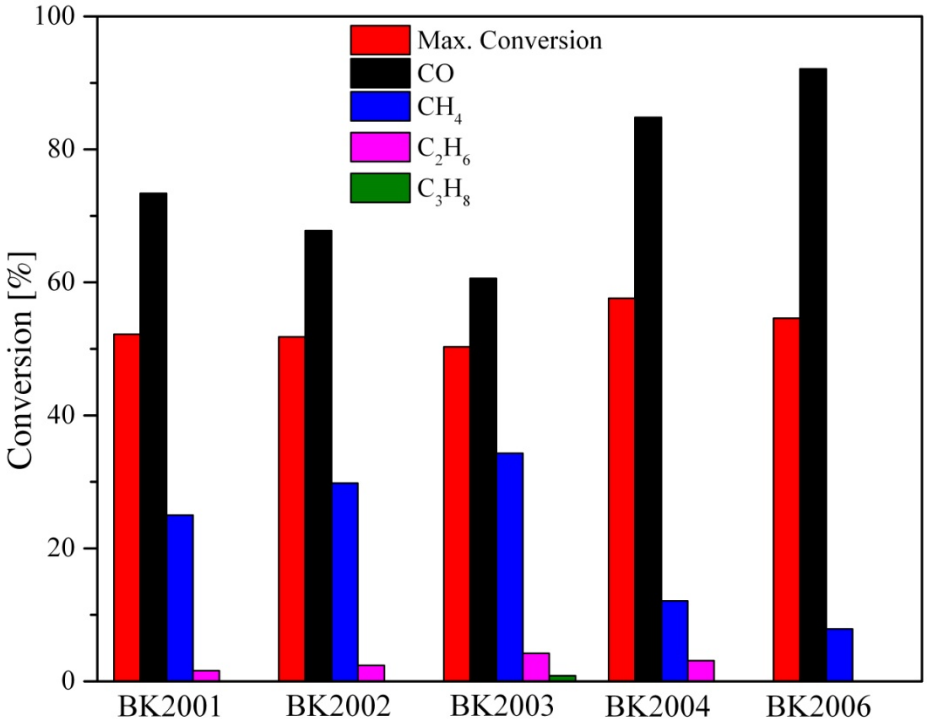

ESI Figure S35. Catalytic effect of the thermal decomposition products of compound **1** in CO_2 hydrogenation.

ESI Text S1

Permanganate bands assignments

The symmetric stretching modes of permanganate ion in compound **1**, ν_1(A or B) are singlets (3x1 A_u +3x1 B_u, 3x1 A_g + 3x1 B_g), the antisymmetric stretching modes ν_3 (2F_2) are triplets (3x2x3=18A_u, 3x2x3=18B_u, 3x2x3=18A_u and 3x3x3=18B_u bands). The symmetric deformation modes ν_2 (2E) are doublets (3x2x2=12 IR (A_u, B_u) and 3x2x2=12 Raman (A_g, B_g) and antisymmetric deformation modes ν_4 (2F_2) are triplets (3x2x3=18A_u, 3x2x3=18B_u, 3x2x3=18A_u and 3x3x3=18B_u bands). The above-mentioned doublets and triplets originate from the splitting of the site group (the symmetry of all permanganate anions is the lowest possible, i.e. C_1).

Among the four normal modes of a permanganate ion, the stretching modes are in the analytical IR range, whereas the two deformation modes are basically in the far-IR region. All four normal modes could easily be assigned both in the IR and Raman spectra of compound **1**. Among the two stretching modes, the ν_{as}(Mn-O) (n_3) is of the highest intensity band in the IR at 909 and 899 cm^{-1}, whereas ν_s(Mn-O) is a weak intensity band at 838 cm^{-1}. The ν_{as}(Mn-O) band splits into three components (913, 903, and 895 cm^{-1}) on cooling to liquid nitrogen temperature, the ν_s(Mn-O) (ν_1) band, however, hardly changes (839 cm^{-1}). The δ_{as} deformation mode of permanganate ion appears as a wide band at 375 cm^{-1} with a shoulder at 362 cm^{-1}. The wide bands of δ_{as}(Mn-O) and the (Fe-O) at 307 cm^{-1} completely overlap with the weak δ_s(Mn-O).

In the Raman spectra of compound **1**, the ν_s(Mn-O) is the highest intensity seemingly singlet band at 840 cm^{-1} (ESI Figure S12). This singlet of compound **1** in the Raman spectrum indicates that at room temperature, one could not distinguish the three crystallographically different permanganate anions (if that were possible, three singlets would appear). A doublet of low-intensity ν_{as}(Mn-O) appears at 922 and 905 cm^{-1}, whereas the deformation modes appear as a doublet at 392/381 cm^{-1} (δ_{as}(Mn-O) (n_4)), and as a wide band at 352 cm^{-1} (δ_s(Mn-O), n_2)). On cooling to 100 K, the ν_{as}(Mn-O) decomposes into four bands and 2 shoulders (917,911, 905, 898, 893sh, and 889sh). Similarly, the δ_s(Mn-O) shows two band groups with 382sh, 380, and 378sh, or 372, 369sh), whereas δ_{as}(Mn-O) decomposed into two bands (338 and 335 cm^{-1}).

Urea vibrational modes

Two strong groups of bands were assigned to the NH_2 stretching modes in the IR spectra of compound **1** recorded at 298 and 100 K (ESI Figure S11 and ESI Table S12). The band group above 3400 cm^{-1} could tentatively be assigned to the antisymmetric, whereas the band group between 3400 and 3300 cm^{-1} to the symmetric N-H modes. The wavenumber of N-H stretching modes in the IR spectra of compound **1** is higher than those in the IR spectra of gaseous urea. The latter phenomenon is interesting by itself and deserves more attention to be paid in the future. Due to the presence of six different crystallographic types of urea molecules (four non-equivalent equatorial and 2 non-equivalent axial ligands), some shoulders and (at 100 K), a band splitting could be observed. The three weak bands in the region of 3330 and 3230 cm^{-1} are most probably second-order transitions of the δ_s(N-H) and the ν(C=O) modes [37]. The Raman bands of N-H stretching modes could be observed as low intensity and wide both at 298 and 123 K (ESI Figure S12).

The N-H deformation, C=O stretching, and C-N stretching modes of compound **1** are located in the 1700-1400 cm^{-1} spectral range. The assignment of the $\delta_{as}(NH_2)$ and δ_{as}(CN) modes in the room temperature IR spectrum are unambiguous, and these appear at 1628 and as a doublet at 1465 and 1446 cm^{-1}, respectively. No splitting on cooling to 100 K could be observed.

The asymmetric and symmetric $\rho(NH_2)$ bands appear at 1143 and 1036 cm^{-1} in the room temperature IR and at 1138 or 1068 cm^{-1} in the room temperature Raman spectra of compound **1**, respectively. The weak ν(CN) band could not be found in the spectra of compound **1**. This band appears at 936 and 1003 cm^{-1} in the IR spectra of gaseous and solid urea. The δ(C=O) and δ(C-N) deformation and the out-of-plane modes of urea in compound **1** were also assigned and given in ESI Table S12. The presence and strength of hydrogen bonds influence the positions of the NH_2 rocking mode as well. The band positions shift to a lower frequency compared to the case of solid urea [37]. However, the $\rho_{as}(NH_2)$ shifts to lower, whereas $\rho_s(NH_2)$ shifts to higher wavenumbers towards the appropriate bands of gaseous urea. The NH_2 and C=O deformation modes shifted to a higher wavenumber compared to the gaseous urea as a consequence of hydrogen bonding.
